# Prevention of violence against women and girls: A cost-effectiveness study across 6 low- and middle-income countries

**DOI:** 10.1371/journal.pmed.1003827

**Published:** 2022-03-24

**Authors:** Giulia Ferrari, Sergio Torres-Rueda, Esnat Chirwa, Andrew Gibbs, Stacey Orangi, Edwine Barasa, Theresa Tawiah, Rebecca Kyerewaa Dwommoh Prah, Regis Hitimana, Emmanuelle Daviaud, Eleonah Kapapa, Kristin Dunkle, Lori Heise, Erin Stern, Sangeeta Chatterji, Benjamin Omondi, Deda Ogum Alangea, Rozina Karmaliani, Hussain Maqbool Ahmed Khuwaja, Rachel Jewkes, Charlotte Watts, Anna Vassall

**Affiliations:** 1 London School of Economics and Political Science, London, United Kingdom; 2 London School of Hygiene & Tropical Medicine, London, United Kingdom; 3 University of Bristol, Bristol Medical School, Bristol, United Kingdom; 4 Gender and Health Research Unit, South African Medical Research Council, Cape Town, South Africa; 5 Health Economics Research Unit, KEMRI-Wellcome Trust Research Programme, Nairobi, Kenya; 6 Nuffield Department of Medicine, University of Oxford, Oxford, United Kingdom; 7 Kintampo Health Research Centre, Kintampo, Ghana; 8 School of Public Health, University of Rwanda, Kigali, Rwanda; 9 Health Systems Research Unit, South African Medical Research Council, Cape Town, South Africa; 10 National Institute of Public Administration, Lusaka, Zambia; 11 Johns Hopkins Bloomberg School of Public Health, Baltimore, Maryland, United States of America; 12 Ujamaa Africa, Nairobi, Kenya; 13 Department of Population Family & Reproductive Health, School of Public Health, College of Health Sciences, University of Ghana, Legon, Ghana; 14 Department of Community Health Science, Aga Khan University, Karachi, Pakistan; 15 School of Nursing & Midwifery, Aga Khan University, Karachi, Pakistan; The University of Edinburgh Usher Institute of Population Health Sciences and Informatics, UNITED KINGDOM

## Abstract

**Background:**

Violence against women and girls (VAWG) is a human rights violation with social, economic, and health consequences for survivors, perpetrators, and society. Robust evidence on economic, social, and health impact, plus the cost of delivery of VAWG prevention, is critical to making the case for investment, particularly in low- and middle-income countries (LMICs) where health sector resources are highly constrained. We report on the costs and health impact of VAWG prevention in 6 countries.

**Methods and findings:**

We conducted a trial-based cost-effectiveness analysis of VAWG prevention interventions using primary data from 5 randomised controlled trials (RCTs) in sub-Saharan Africa and 1 in South Asia. We evaluated 2 school-based interventions aimed at adolescents (11 to 14 years old) and 2 workshop-based (small group or one to one) interventions, 1 community-based intervention, and 1 combined small group and community-based programme all aimed at adult men and women (18+ years old). All interventions were delivered between 2015 and 2018 and were compared to a do-nothing scenario, except for one of the school-based interventions (government-mandated programme) and for the combined intervention (access to financial services in small groups). We computed the health burden from VAWG with disability-adjusted life year (DALY). We estimated per capita DALYs averted using statistical models that reflect each trial’s design and any baseline imbalances. We report cost-effectiveness as cost per DALY averted and characterise uncertainty in the estimates with probabilistic sensitivity analysis (PSA) and cost-effectiveness acceptability curves (CEACs), which show the probability of cost-effectiveness at different thresholds. We report a subgroup analysis of the small group component of the combined intervention and no other subgroup analysis. We also report an impact inventory to illustrate interventions’ socioeconomic impact beyond health. We use a 3% discount rate for investment costs and a 1-year time horizon, assuming no effects post the intervention period. From a health sector perspective, the cost per DALY averted varies between US$222 (2018), for an established gender attitudes and harmful social norms change community-based intervention in Ghana, to US$17,548 (2018) for a livelihoods intervention in South Africa. Taking a societal perspective and including wider economic impact improves the cost-effectiveness of some interventions but reduces others. For example, interventions with positive economic impacts, often those with explicit economic goals, offset implementation costs and achieve more favourable cost-effectiveness ratios. Results are robust to sensitivity analyses. Our DALYs include a subset of the health consequences of VAWG exposure; we assume no mortality impact from any of the health consequences included in the DALYs calculations. In both cases, we may be underestimating overall health impact. We also do not report on participants’ health costs.

**Conclusions:**

We demonstrate that investment in established community-based VAWG prevention interventions can improve population health in LMICs, even within highly constrained health budgets. However, several VAWG prevention interventions require further modification to achieve affordability and cost-effectiveness at scale. Broadening the range of social, health, and economic outcomes captured in future cost-effectiveness assessments remains critical to justifying the investment urgently required to prevent VAWG globally.

## Abstract 2

In a cost-effectiveness study, Dr. Giulia Ferrari and colleagues examine the costs and health impact of prevention of violence against women and girls in six low- and middle-income countries.

## Author summary

**Why was this study done?:**

- Governments are increasing funding for the elimination of violence against women and girls (VAWG) by 2030 as part of sustainable development goal five (SDG5).
- The evidence to inform investment in this area is extremely limited, including from low- and middle-income countries (LMICs), presenting a major obstacle to scaling up violence against women prevention programming.
- Investigating the potential cost-effectiveness, health, and nonhealth impacts of prevention helps those working in violence against women prevention justify funding from the health sector or other sectors interested in health improvement and women’s well-being.

**What did the researchers do and find?:**

- We report trial-based cost-effectiveness estimates for 6 interventions designed to prevent VAWG in 6 countries: Ghana, Kenya, Pakistan, Rwanda, South Africa, and Zambia.
- We find that some interventions are likely to improve population health, even within current health budgets in each country. Interventions are more likely to be cost-effective at preventing women’s exposure to violence, rather than men’s perpetration. One-to-one psychosocial support interventions for secondary prevention, while impactful, are likely to be less cost-effective than primary prevention interventions in low-resource settings. Community- and school-based interventions are more likely to be cost-effective from a health sector perspective.
- Considering all effects, interventions that improve participants’ livelihood skills, including their financial management skills, can be cost saving, while also reducing perpetration of violence from men, even if they do not reduce experience of violence among women and girls in the short term.

**What do these findings mean?:**

- The evidence suggests that established community-based interventions to prevent violence against women warrant consideration for immediate scale-up.
- However, to reach all populations in need with appropriate interventions, more investment is required to further develop and refine a range of prevention delivery models that are impactful and contain costs, while developing the human resource expertise in LMICs.
- Research funding is required to continue to enable rigorous impact, process, and economic evaluation of VAWG prevention that captures impact not only on violence exposure and perpetration, but also broader health and nonhealth impacts to ensure that the strongest case for investment in the prevention of VAWG continues to be made globally.

## Introduction

Twenty-seven percent (uncertainty interval 23% to 31%) of women and girls aged 15 years or older have experienced either physical or sexual intimate partner violence (IPV) or nonpartner sexual violence globally [1]. Sustainable development goal five (SDG5) includes the elimination of all violence against women and girls (VAWG) by 2030. To support the achievement of this goal in low- and middle-income countries (LMICs), official development assistance (ODA) to end VAWG have been steadily increasing since 2016 [2]. A critical challenge is to develop feasible and impactful prevention interventions that can be rapidly scaled up and sustained within the fiscal limits of LMIC governments and their development partners.

Funded by the Department for International Development (DFID) in the United Kingdom, What Works to Prevent Violence is the first coordinated programme to conduct evaluation across multiple countries using randomised controlled trials (RCTs) to assess both the impact and cost-effectiveness of VAWG prevention, including IPV, in LMICs. Evidence from What Works [3] and other programmes suggests that preventing VAWG is feasible within tight programmatic timelines across a number of settings and platforms. Community-based interventions have shown promise [4,5] but, depending on the setting, only certain modes of delivery may be effective [6]. School-based interventions have reduced corporal punishment [7] and sexual assault against girls [8]: In Uganda, an RCT of an intervention that trained teachers to avoid using corporal punishment found a reduction in teachers’ physical violence against students at 18-month follow-up (odds ratio 0.40, 95% CI (0.26 to 0.64), *p* < 0.0001) [7]. Likewise, a cluster randomised matched pairs parallel trial of a behaviour-based intervention in Nairobi’s informal settlements similar to the one in our study reported a reduction in the risk difference of exposure to rape equal to 3.7% for schools in the intervention arm compared to those in the control arm (95% CI (0.4% to 8.0%), *p* < 0.03) [8]. There is little evidence of the impact on violence from curriculum-based interventions in schools [9]. However, a recent systematic review found that 50% of adolescent dating violence programmes are effective in both high- and middle-income countries [10].

For adults, workshop-based interventions have also been found to be highly effective [11,12], particularly with specific subpopulations [12]. Workshop-based interventions have shown promise in South Africa, among poor women eligible for access to microfinance services in peri-urban areas and among unemployed youth [13]. Some prevention interventions that aim to improve broad economic well-being have demonstrated social and financial benefits for participants [14,15], but may not reduce exposure to IPV in the short run [16] and exacerbate it at times [17]. Recent reviews of the IPV impact of economic interventions show that in some cases, these interventions have no effect on IPV exposure [18], and, in some cases, they may increase exposure to IPV, especially in terms of controlling behaviour and economic violence [17]. Finally, evidence from high-income settings suggests that VAWG prevention reduces the costs of police and criminal justice system, property damage, and clinical health [19].

Despite the emerging evidence and high-level policy and public commitment to reducing VAWG, funding for VAWG prevention remains scarce due to fiscal constraints on health, education, and social service provision in LMICs. While the moral arguments for the prevention of VAWG are clear, evidence of the cost-effectiveness of VAWG prevention is often a prerequisite for significant increases in both domestic and development assistance funding. However, to date, the evidence on cost-effectiveness of VAWG prevention is inconclusive, with only 3 published cost-effectiveness studies of VAWG prevention available. The Intervention with Microfinance for AIDS and Gender Equity (IMAGE) offered women access to microcredit services and life skills training/community mobilisation techniques [11]. IMAGE is cost-effective against the 1xGDP threshold, but not against the health sector opportunity cost threshold, but the intervention’s cost-effectiveness was low compared to other basic health services in LMICs [20]. The SASA! community mobilisation intervention in Uganda reported a 52% nonstatistically significant reduction in past year experience of physical IPV [4], but as the study did not measure cost per disability-adjusted life year (DALY) averted, a standardised measure of health outcomes, the intervention cannot be compared to investment in other health interventions. SASA! reported a cost per year free from physical IPV of 2011 US$460 [21]. Unite for a Better Life (UBL), a combined workshop-based and community mobilisation in Ethiopia, reported a cost per year free from physical and/or sexual IPV of 2015 US$194 at the community level and 2015 US$2,726 for workshop participants [22]. Cost per year free from IPV among UBL workshop participants are in line with IMAGE’s (2004 US$710) [23]; as expected, community-level results are more favourable. However, they cannot be compared to a cost-effectiveness threshold, as they are not expressed in terms of cost per DALY averted. Moreover, UBL’s estimate includes women only, men only, and couple’s interventions; SASA! only refers to physical IPV and IMAGE reports on physical and/or sexual IPV of a women-only intervention. Hence, we recommend caution when comparing the cost per year free from IPV of these 2 interventions.

This paper presents the first standardised multicountry cost-effectiveness analysis, to our knowledge, of interventions for the prevention of VAWG, employing methods commonly used to justify investment in the health sector to assess costs, impact on burden of disease, and cost-effectiveness [24]. It reports cost and cost-effectiveness in research settings and at scale, using routine implementation scenarios derived in a previous paper [25]. We only report observed direct effects, neither accounting for future direct effects nor for any indirect effects, except for economic impact where this is available. Our estimates may thus underestimate total intervention effect. However, direct effects are estimated within RCT settings, usually characterised by higher effectiveness than routine implementation, potentially leading to an overestimate of interventions’ direct impact on IPV prevention.

These findings will be of interest to researchers in the field of women’s empowerment and violence prevention, to health and social policy makers and to implementers.

## Methods

### Overview

We report on the cost-effectiveness, health outcomes, and broader societal impact of 6 VAWG prevention interventions in Ghana, Kenya, Pakistan, Rwanda, South Africa, and Zambia, using a trial-based analysis. These 6 interventions were selected out of the 10 interventions evaluated using RCTs for the What Works programme [3]. We selected the interventions that were comparatively more established, as they had a higher likelihood of being considered for adoption by policy makers if effective. We also selected them to be representative of delivery platforms, target mechanisms, populations (adults, children; females and males), types of violence addressed, and geographies (Southern Africa and South Asia) available within this group. Full details of all What Works studies are available on www.whatworks.co.za. It was not our aim to compare one intervention across sites or to compare interventions’ cost-effectiveness across sites as each context is different. Rather, we synthesise findings on the cost-effectiveness of 6 cutting-edge implementation approaches in this field conducted as part of one cohesive global programme of research.

We assessed cost-effectiveness using a standardised methodology we developed for VAWG prevention and women’s empowerment interventions [26], by adapting state-of-the-art methods for economic evaluation in health and medicine [24,27,28] to the characteristics of behavioural interventions for the empowerment of women and girls and the prevention of VAWG: This analysis meets the requirement of the reference case set out by the Second Panel on Cost-Effectiveness in Health and Medicine [29].

Our primary outcome measure is cost per DALY averted. We report cost per DALY averted from both a provider and societal perspective, the former examining health sector costs and outcomes only, and the latter including economic impact. A societal perspective is particularly relevant for IPV prevention, because they are designed to impact recipients’ well-being beyond health domains. We estimate health and IPV effects for the economic evaluation using the same statistical models used to estimate interventions’ primary outcomes, as described in the analysis section below and in Table 1. We also present an inventory of broad social (nonhealth) impact [24]. We compared 6 VAWG prevention interventions to the status quo (control groups) in each country, for trial populations over the time period of the trials (Table 1). We then estimated potential cost-effectiveness should VAWG prevention be delivered to the entire eligible population for each country, assuming impact can be maintained at scale.

**Table 1:** Intervention descriptions—Research setting.

	RRS	IMpower	RTP	Indashyikirwa	SSCF	VATU
**Key study design characteristics and processes**						
**Setting**	Ghana (1), rural and urban	Kenya, urban (informal settlements)	Pakistan, urban	Rwanda, rural	South Africa, urban (informal settlements)	Zambia, urban (high population density, low-income compounds)
**Location**	Central region (2 districts)	Nairobi	Hyderabad (Sindh Province)	Eastern, Northern, Western provinces (7 districts)	Durban	Lusaka
**Intervention sites**	20 communities	52 schools	20 schools	14 sectors	17 sites	3 sites (123 families, 65 of which also included 1 child in the study)
**Target population**	Female (18 to 49 years) and male (> = 18 years) adults who usually live in the household and have lived in the community for at least 1 year	Female children in primary schools (11 to 14 years old (2), in grades 5 to 8)	Schools: single sex, public middle schools in Hyderabad with playground or indoor space that can host 35 or more students for games. Students: male and female children in primary schools (grades 6 to 8)	Adults (18 to 49 years old) resident in the community for at least 6 months; married or living with current partner for at least 6 months, not participating in the Indashyikirwa couples’ intervention	Not formally employed female and male adults (18 to 30) who normally reside in informal settlement cluster	Families living in the study compounds in Lusaka with at least 1 female and 1 male adult (18+), and 1 child between 8 and 17 years old identified by the mother as the most affected by the violence. The adult female must report (i) at least moderate violence within the family as defined above; and (ii) hazardous alcohol use by the adult male in the household. The latter must be confirmed in the adult male’s screening
**Number of potential participants**	73,759	24,055	15,968	141,733	677	246
**Control group**	Do nothing	Ministry of Education mandated “life skills course,” a one 2-hour session on sexual and reproductive health and general life skills. The session was delivered by Ujamaa facilitators in control schools	Do nothing	VSLA only (VSLA alone)	Do nothing	Safety checks
**Time horizon**	One year; 24 months postbaseline	One year; 24 months postbaseline	One year; 24 months postbaseline	One year; 24 months postbaseline	One year; 24 months postbaseline	One year; 12 months postbaseline
**Cost data collection**	July 2017 to September 2018	October 2017 to September 2018	September to October 2019	September 2017 to December 2018	May 2018 to September 2018	May 2017 to September 2018 (preliminary interviews and financial data: 2016)
**Intervention characteristics**						
**Implementing organisation(s)**	Gender Studies and Human Rights Documentation Centre	Ujamaa	RTP Pakistan	CARE Rwanda, RWN and RWAMREC	Project Empower	SHARPZ, Johns Hopkins University
**Approach**	Addressing harmful social norms on gender and violence	Empowerment and self-defense	Play based	Addressing harmful social norms on gender and violence	Gender transformative and livelihoods strengthening	Psychotherapeutic support
**Platform of delivery**	Community based	School based to single-sex classes after/during school day	School based to classes during school	Community-based and small groups	Small groups	One-to-one sessions
**Number of implementation sessions or duration**	18 months	Six 2-hour sessions, plus 2 booster sessions, at 6 and 10 months, respectively	One-hundred twenty 35-minute sessions over 2 years, conducted separately for boys and girls	30 months	Twenty-one 3-hour sessions, delivered twice a week to single sex groups of approximately 20 over 4 to 6 months per group	12 one-to-one weekly sessions over a period of 12 weeks
**Year(s) intervention developed**	2002	2009 to 2011 (3)	2008 to 2014	2013 to 2016	2011 to 2013	2010
**Start-up phase**	January to December 2016	October 2009 to March 2016	January 2015 to February 2018	October 2015 to May 2016	December 2011 to December 2015	September 2015 to May 2016
**Implementation phase**	December 2016 to December 2017	January to September 2016	November 2015 to February 2018	September 2016 to July 2018	January 2016 to March 2017	June 2016 to December 2017
**Outcomes**						
**Primary violence outcomes**	Past year incidence of IPV (perpetration of physical and/or sexual IPV for men and experience for women)	Sexual assault within past 12 months	Peer violence victimisation in the past 4 weeks; peer violence perpetration in the past 4 weeks	IPV (sexual or physical; experience and perpetration)	Any past year physical IPV perpetration (men) and experience (women); any past year sexual IPV perpetration (men) and experience (women); past year severe sexual and/or physical IPV perpetration (men) and experience (women); controlling behaviours	Change in violence against women as measured by SVAWS
**Other violence outcomes**	Institutional assessment of violence against women cases [time frame: 3 years]; reported cases of violence against women IPV (emotional violence; economic violence); nonpartner violence	Recurrent physical and/or sexual IPV; forced or coerced sex with main partner; physical IPV, emotional IPV; help seeking among survivors of IPV; children in household witnessing IPV, emotional violence	Corporal punishment in the past 4 weeks; physical punishment at home in past 4 weeks	Recurrent physical and/or sexual IPV; forced or coerced sex with main partner; physical IPV, emotional IPV; sources of information on IPV and number of times heard; help seeking among survivors of IPV; economic abuse with main partner; children in household witnessing IPV; change in strategies used to address IPV	IPV (emotional violence; economic violence); nonpartner violence	Change in child abuse as measured by the Youth Victimization Scale; change in psychological violence as measured by Index of Psychological Abuse
**Other health outcomes**	Hazardous alcohol use, drug use, depression, abortion	Alcohol and drug use; PTSD; depression and anxiety at 24 months; self-efficacy and well-being	Depression	Hazardous alcohol use	Hazardous alcohol use, drug use, depression, suicidal ideation, life circumstances, last sexual partner, transactional sex past year, stress about lack of work	Change in alcohol abuse as measured by AUDIT; Change in depression symptoms as measured by the CES-D; Change in PTSD symptoms (adult) as measured by the HTQ; Change in substance use as measured by ASSIST
**Nonhealth outcomes**	Income, gender attitudes	Gender norms	School attendance, school performance, early marriage, gender attitudes	Income; gender attitudes; support for women working outside the home	Earnings in past month; gender attitudes, consumption and savings, life circumstances, shame about lack of work, mobilisation of money in an emergency, stealing because of hunger in past month	Change in belief about gender norms as measured by the GEMS
**Primary outcome for the economic evaluation**	Past year incidence of IPV (perpetration of physical and/or sexual IPV for men and experience for women) captured by WHO measures	Past year incidence of IPV (experience of physical IPV for female children) captured by WHO measures	Incidence of peer violence victimisation in the past 4 weeks as measured with the PVS	Past year incidence of IPV (perpetration of physical and/or sexual IPV for men and experience for women) captured by WHO measures	Past year incidence of IPV (perpetration of physical and/or sexual IPV for men and experience for women) captured by WHO measures	Past year incidence of IPV (perpetration of physical and/or sexual IPV for men and experience for women) captured by WHO measures
**Study-level statistical analysis for intervention effect estimates**						
**Statistical model**	Difference in differences	Generalised linear mixed model for change	Generalised linear mixed model for change	Generalised linear mixed model for change	Generalised linear model first difference	Generalised linear mixed model for change
**Level of analysis**	Village summaries	Student	Student	Individual	Individual	Individual
**Link function**	N/A	Binary outcomes: Logit (continuous outcomes: Gaussian)	Binary outcomes: Logit (continuous outcomes: Gaussian)	Binary outcomes: Logit (continuous outcomes: Gaussian)	Binary outcomes: Logit (continuous outcomes: Gaussian)	Binary outcomes: Logit (continuous outcomes: Gaussian)
**Random effects (level)**	No	Yes (cohort, cluster, individual)	Yes (school)	Yes (sector)	No	Yes (counsellor, couple)
**Robust standard errors**	No	Yes	No	No	Yes, clustered at settlement level	Yes
**Effect estimate**	Difference in differences	Adjusted change in odds ratios (differences)	Adjusted odds ratios (differences) at 24 months	Adjusted change in odds ratios (differences)	Adjusted odds ratios (differences) at 24 months	Adjusted change in odds ratios (differences)

ASSIST, Alcohol, Smoking, and Substance Involvement Screening Test; AUDIT, Alcohol Use Disorders Identification Test; CES-D, Center for Epidemiological Studies Depression Scale; GEMS, Gender Equitable Men’s Scale; HTQ, Harvard Trauma Questionnaire; IPV, intimate partner violence; PTSD, post-traumatic stress disorder; PVS, Peer Victimization Scale; RRS, Rural Response Systems; RTP, Right To Play; RWAMREC, Rwanda Men’s Resource Centre; RWN, Rwanda Women’s Network; SSCF, Stepping Stones and Creating Futures; SVAWS, Severity of Violence Against Women Scale; VATU, Violence and Alcohol Treatment; VSLA, village savings and loan association; WHO, World Health Organization.

We estimated the incremental cost of all interventions using primary data collected during the trial period. We mapped the processes and costed the resources used in developing, adapting, setting up, and implementing the interventions; see Torres-Rueda and colleagues [25] for more details. Health and other outcomes were estimated directly from the end line surveys for all RCTs, with DALYs calculated using trial participants’ directly reported health outcomes. We also report interventions’ impact on past year exposure and perpetration of violence, and a cost per year free of violence. We do not include potential posttrial health benefits, conservatively assuming no sustained costs nor health, economic, or social impact.

### Study setting and interventions

Selected interventions employed 3 types of delivery platforms: classes within schools, community mobilisation, and one-on-one or small group workshops (including counselling) and were delivered in urban (Kenya, Pakistan, South Africa, and Zambia) and rural settings (Ghana and Rwanda). The interventions targeted different impact mechanisms defined using theories of change: economic empowerment alongside a gender empowerment component (South Africa and Rwanda); psychological empowerment (Kenya and Pakistan), including self-defense (Kenya) and psychotherapeutic (Zambia); gender attitudes and behaviours (South Africa, Ghana, Pakistan, and Rwanda), and social norms change (Ghana and Rwanda) (Table 1). Published protocols [30] and impact evaluations [6,31–36] contain more details on each intervention.

The interventions address the needs of different population groups experiencing violence (Table 1). Interventions in Ghana, Rwanda, and South Africa targeted adult populations (18+); for the Zambian intervention, we only report results for adults (18+) because the study was not powered to report on children. The intervention in Kenya targeted 11- to 14 year-old school-going girls, and the study was powered to detect the intervention’s effect on girls’ exposure to sexual assault. The study collected data on boys as an exploratory outcome, but these data were not available at the time of writing. The Right To Play (RTP) intervention in Pakistan sought to reduce the perpetration and experience of peer-to-peer violence among 11- to 14-year-old school-going children [30]. All but the Pakistani and Kenyan interventions measured sexual and/or physical IPV among adults [5,6,16,31,32] as their primary outcome. All studies except Violence and Alcohol Treatment (VATU, Zambia) used the World Health Organization (WHO) IPV measure. VATU used WHO measure as a secondary outcome and the Conflict Tactics Scale (CTS) as primary [31]. Comparator interventions (the status quo) (i.e., control arm) were mainly “do nothing” except for Kenya and Rwanda (Table 1).

### Outcomes

To estimate incremental DALYS averted by each intervention compared to the status quo, we estimated the DALYs attributable to the health sequelae, i.e., health consequences, found for each intervention using trial data (Table 2). Several studies identify a number of physical and psychological health consequences of IPV. The link between the health sequelae we include and IPV is well established [37]. See Fig A in S1 Appendix for a more detailed list. We also report cost per year free of violence in the Appendix (Table G in S1 Appendix). This information can be useful for VAWG prevention programming. The outcomes for each RCT were defined differently by each RCT research team so health sequelae measured varied by each RCT (Table A in S1 Appendix). For community interventions, study participants were a randomly selected representative sample of the total population receiving the intervention (Table 1). To arrive at cost per participant, we divide total costs by the number of recipients. The health sequelae we included when estimating DALYs for the adult populations are the following: having a moderately high level of depression (Center for Epidemiological Studies Depression Scale [CES-D] 20 ≥22), past year drug use, and hazardous alcohol use (The Alcohol Use Disorders Identification Test Consumption questions [AUDIT-C] ≥4 for males, AUDIT-C ≥3 for females) (Table A in S1 Appendix). The sequelae were measured with the same instruments across interventions targeting the adults, except the Rwandan RCT that measured alcohol use (AUDIT-C) only. Pakistan measured depression among children using the Child Depression Inventory II^32^ to identify cases of depression (CDI ≥65 points). The Kenyan study measured anxiety and depression in children using the Beck Youth Depression and Anxiety Inventories (BDI-Y and BAI-Y, respectively) (Table A in S1 Appendix). Cutoff thresholds were not publicly available for the Youth Self-Report (YSR), the measure of depression used for children in the Zambia intervention. Moreover, the study was not powered to report the intervention’s impact on children, and they were not included in our DALY estimates. We measure peer violence in Pakistan with the Peer Victimization Scale (PVS) [38], the study’s primary outcome. For all other interventions, we measure exposure to physical or sexual IPV using the WHO measure.

### Cost estimation

We measured providers’ resource use and economic costs with a bottom-up microcosting approach [26]. We identified the activities necessary to develop, adapt, set up, and implement the interventions alongside the RCTs (research setting) by conducting semistructured interviews or direct observation, where possible, and reviewing financial documents, monitoring and evaluations records, and other log books. We entered the data in standardised costing workbooks [39] to estimate the cost per participant reached, using an intention-to-treat approach. For more details on the estimation and analysis of providers’ costs, see Torres Rueda and colleagues [25] and the S1 Appendix (p. 1–4).

We report costs for 2 settings: costs of “implementation in a research setting” and “implementation in a routine setting.” The costs of implementation in a research setting were the costs measured in the trials, but excluded all direct costs related to research (e.g., monitoring and evaluation activities only required for study purposes). The costs of implementation in a routine setting were then estimated for 2 national scale-up scenarios, using assumptions about the level of coverage and size of the organisations delivering interventions at scale. Details on the process used to derive relevant assumptions are published elsewhere [25] (S1 Appendix, p. 3–4).

We collected information on adult participants’ economic outcomes as part of the RCTs. We used past year consumption as a proxy for disposable income to measure the economic (productivity) impact of an intervention. Consumption is a preferable measure to report income for productivity, given the low proportion on waged earners in LMICs [40] and a low propensity to save in poor households [41]. We estimated the intervention’s incremental impact on adult participants’ productivity compared to controls with the same statistical models used for the RCTs health outcomes. We then subtracted any estimated per capita productivity gain from the per capita cost of the intervention to obtain the net societal cost of the intervention for the incremental cost-effectiveness ratio (ICER) from a societal perspective, following current cost-effectiveness analysis guidelines [24].

### Analysis

#### Effect estimates

We estimate impact using the statistical model used to generate the RCT primary and secondary results, in line with previously published results, reporting estimates for females only and for females and males jointly (all). For Ghana, impact is estimated using a linear difference-in-differences model on village summaries [5]. Kenya, Pakistan, Rwanda, and Zambia estimate the difference in differences (or ratio of risk ratios) using generalised mixed models that reflect trial design and adjust for any baseline imbalances [6,31,32]. South Africa estimates impact at 24 months adjusting only for each baseline outcome variable of interest (first difference), having found no baseline imbalances in other predictors [36]. For Kenya, we do not adjust for baseline scores of the outcome of interest because depression and anxiety were only measured at end line.

We first report interventions’ impact on IPV and on the health outcomes used in our DALY estimation from the RCTs. To compute the total number of IPV-free (or peer violence) years gained, we first estimated the adjusted difference in absolute risk of IPV (or peer violence) exposure or perpetration between intervention and control group; we then multiplied the adjusted risk difference by the total number of participants randomised to the treatment arm at baseline (equivalent to an intention-to-treat approach) to arrive at the total number of IPV-free (or peer violence) years gained. To estimate incremental DALYs averted, we computed the adjusted risk difference in DALYs averted between intervention and control group.

#### Cost-effectiveness estimates

We assessed cost-effectiveness for females and males (all) and for females only. All analyses were conducted in USD 2018 prices, and provider costs were discounted using a rate of 3% (further details on exchange rates and other conversions can be found in S1 Appendix, p. 2). DALYs were not discounted because we assume no effect beyond the trial period. We assessed cost-effectiveness by estimating the probability that the incremental cost per DALY averted (or ICER) is cost-effective using 2 different thresholds to define when an intervention is “declared” as cost-effective employed by Ministries of Health/public funders. The first compares cost per DALY averted from a provider perspective to each country’s “opportunity cost” threshold [20]. Opportunity cost thresholds assess whether an intervention performs better than the least efficient in the health sector. As such, they measure when funding a new intervention will improve population health if funded within current government health sector budgets. The second threshold is applied to the societal perspective cost-effectiveness ratios. Here, the probability that each intervention is cost-effective the “societal” ICER is compared against each country’s per capita GDP in 2018. This provides an example of threshold from the perspective of a societal decision-maker who may consider funding beyond the health sector. We also present cost-effectiveness acceptability curves (CEACs) for those adopting different thresholds.

To account for the uncertainty in ICER estimates, we performed a probabilistic sensitivity analysis (PSA) to generate 10,000 incremental cost and DALY pairs assuming gamma distributions on provider costs and normal distributions on DALYs and net costs. We parametrised the distributions on health and economic outcomes using the within-trial estimates and the distributions for provider costs with values of alpha and beta such that their product yielded the mean cost per participant that emerged from our cost analysis [25]. We tested distributional fit with standard tests (Tables C and D in S1 Appendix, p. 8). We used the results of the PSA to plot CEACs, which show the proportion of simulations that are cost-effective at the two thresholds stated above.

We conducted several deterministic sensitivity and scenario analyses. First, we explored how our results would be influenced if we replaced our direct measurement of health outcomes with an indirect measure of DALYs averted per IPV or peer victimisation case averted. Prevention interventions act on health sequelae in different ways, not necessarily related to IPV (e.g., a participant’s depression may also be due to other factors) and not all RCTs measured all possible health outcomes (Fig A and p. 6 in S1 Appendix). We therefore also estimated IPV-only DALYs using estimates of population health outcomes associated with VAWG (used by the Institute of Health Metrics and Evaluation (IHME) to estimate the global burden of disease) together with estimates of the incremental IPV cases averted by the intervention (S1 Appendix, p. 6–7 and 9–10).

Second, we estimated 2 scenarios to adjust the costs from the trial “implementation in a research setting” to those for scaled up “implementation in a routine setting.” This was done using several assumptions. Scenario 1 models the inputs/resources needed to achieve national scale of the intervention trialled. Scenario 2 also modifies delivery processes as discussed in consultations with implementers on how the intervention would likely be implemented at scale (details on the cost analysis are provided in Torres-Rueda and colleagues [25] and S1 Appendix, p. 1–4).

Third, we conducted a subpopulation analysis for Rwanda, where the intervention was divided into phases targeting specific subpopulations. The gender transformative programme in Rwanda was initially offered to village savings and loan associations (VSLAs) participants (male–female couples) in small groups (VSLA-plus). Some of the couples were then trained in community mobilisation using an approach derived from SASA! [4] to encourage shifts in beliefs, attitudes, norms, and behaviours among members of their wider communities. The complete intervention also included opinion leader training and community safe spaces for women. We report a subpopulation analysis of cost-effectiveness on the recipients of the VSLA-plus component, [32] because it was delivered through a distinct platform from the community model (S1 Appendix, p. 11–12).

We did not explore further subgroup analysis by age group, because it is unlikely that implementation would target only specific age groups and not others, as the interventions were designed for the groups they were tested on. We conducted the analysis in Stata 15.1 [42].

### Ethics approval and trials registrations

This study obtained ethics approval from the London School of Hygiene & Tropical Medicine (LSHTM) Ethics Committee (#12204) and all local and research partners’ ethics committees: the Noguchi Memorial Institute for Medical Research, University of Ghana (006/15-16), Kintampo Health Research Centre Institutional Ethics Review Committee IRB (#2017–15) and South Africa MRC (EC031-9/2015) (Ghana); KEMRI Scientific and Ethics Review Unit (KEMRI/RES/7/3/1) and Stanford IRB (#34706) (Kenya); Agha Khan University IRB (#2019-1544-4273) (Pakistan); Rwanda National Ethics Committee (#880/RNEC/2016) and South Africa MRC (EC033-10/2015) (Rwanda); University of Zambia Bioethics Research Ethics Committee (004-11-15) and Johns Hopkins University IRB (#6534) (Zambia); and University of Kwa-Zulu Natal, Biomedical Research Ethics Committee (BFC043/15) and South African MRC (EC006-2/2015) (South Africa). Trials’ registrations on ClinicalTrials.gov are the following: Ghana: NCT03237585; Kenya: NCT02771132; Pakistan: NCT03448523; Rwanda: NCT03477877; South Africa: NCT03022370; and Zambia: NCT02790827.

#### Role of the funding source

The study sponsor, U.K. Aid from the U.K. Government, played no part in study design; in the collection, analysis, and interpretation of data; in the writing of the report; and in the decision to submit the paper for publication.

## Results

Table 2 presents interventions’ impact on the violence and health outcomes we use in the cost-effectiveness analysis (for goodness of fit tests, see Table H in S1 Appendix). The South African intervention reduced VAWG, especially in terms of men’s perpetration. It also reduced depression, although this is imprecisely estimated and did not have an impact on alcohol consumption. The intervention in Ghana achieved moderate impact on VAWG and alcohol consumption and reduced depression (Table 2). The Zambia couples’ psychological intervention achieved substantial reduction of hazardous alcohol use by males, in addition to reducing women’s exposure to and men’s perpetration of VAWG (Table 2). In Rwanda, the community intervention had no impact on VAWG or alcohol misuse; the couples’ intervention reduced VAWG and depression, but records no impact on alcohol misuse (Table E in S1 Appendix). The Kenyan intervention delivered health benefits in the related domains of depression and anxiety for adolescents, but no effect on physical violence from an intimate partner (Table 2). In Pakistan, the intervention achieved large reductions in violence and depression, although both are imprecisely estimated. Each study reported separately on all predeclared primary and secondary outcomes [5,6,31,32,34–36].

**Table 2:** Outcomes at 24 months by intervention arm (24 months postbaseline, unless otherwise specified)^.

	Women and girls only						All (exposure and perpetration)^§^					
		IPV	Peer to peer victimisation	Depression	Hazardous alcohol use	Anxiety		IPV	Peer to peer victimisation	Depression	Hazardous alcohol use	Anxiety
**RRS** ^‡^	**Intervention percentage (*N* = 1,030 in 9 villages)**	0.12		0.20	0.08		**Intervention percentage (*N* = 1,942 in 20 villages)**	0.20		0.18	0.10	
	**Control percentage (*N* = 1,168 in 14 villages)**	0.14		0.31	0.07		**Control percentage (*N* = 2,088 in 24 villages)**	0.21		0.30	0.10	
	**Difference in differences (*N* = 2,198 in 46 villages)**	−0.03		−0.12	−0.01		**Difference in differences (*N* = 4,030 in 88 villages)**	−0.03		−0.05	−0.03	
	**(95% CIs)**	(−0.08 to 0.02)		(−0.18 to −0.05)	(−0.12 to 0.10)		**(95% CIs)**	(−0.08 to 0.01)		(−0.10 to −0.00)	(−0.13 to 0.07)	
**IMpower**	**Intervention percentage (*N* = 1,693)**	0.12		0.13		0.47						
	**Control percentage (*N* = 1,513)**	0.10		0.14		0.46						
	**Adjusted odds ratio (*N* = 3,206)**	1.17		0.90		1.06						
	**(95% CIs)**	(0.62 to 2.21)		(0.68 to 1.18)		(0.74 to 1.53)						
**RTP**	**Intervention percentage (*N* = 405)**		0.49	0.04			**Intervention percentage (*N* = 749)**		0.65	0.07		
	**Control percentage (*N* = 372)**		0.67	0.04			**Control percentage (*N* = 648)**		0.76	0.06		
	**Adjusted odds ratio (*N* = 777)**		0.47	0.62			**Adjusted odds ratio (*N* = 1,397)**		0.67	0.64		
	**(95% CIs)**		(0.15 to 1.51)	(0.18 to 2.16)			**(95% CIs)**		(0.20 to 2.30)	(0.27 to 1.48)		
**Indashyikirwa**	**Intervention percentage (*N* = 699)**	0.70			0.04		**Intervention percentage (*N* = 1,399)**	0.59			0.10	
	**Control percentage (*N* = 699)**	0.57			0.04		**Control percentage (*N* = 1,397)**	0.45			0.08	
	**Adjusted odds ratio (*N* = 1,396)**	1.24			0.73		**Adjusted odds ratio (*N* = 2,792)**	1.16			0.79	
	**(95% CIs)**	(0.90 to 1.70)			(0.36 to 1.47)		**(95% CIs)**	(0.94 to 1.44)			(0.54 to 1.15)	
**SSCF**	**Intervention percentage (*N* = 260)**	0.58		0.72	0.29		**Intervention percentage (*N* = 497)**	0.50		0.66	0.39	
	**Control percentage (*N* = 285)**	0.60		0.77	0.28		**Control percentage (*N* = 553)**	0.55		0.72	0.38	
	**Adjusted odds ratio (*N* = 545)**	0.93		0.72	1.03		**Adjusted odds ratio (*N* = 1,049)**	0.82		0.80	1.02	
	**(95% CIs)**	(0.66 to 1.31)		(0.49 to 1.07)	(0.66 to 1.62)		**(95% CIs)**	(0.67 to 1.00)		(0.60 to 1.05)	(0.83 to 1.27)	
**VATU** *	**Intervention percentage (*N* = 100)**	0.51		0.26	0.25		**Intervention percentage (*N* = 198)**	0.50		0.24	0.23	
	**Control percentage (*N* = 114)**	0.58		0.33	0.29		**Control percentage (*N* = 222)**	0.60		0.35	0.34	
	**Adjusted odds ratio (*N* = 216)**	0.30		0.49	0.72		**Adjusted odds ratio (*N* = 432)**	0.34		0.59	0.37	
	**(95% CIs)**	(0.14 to 0.62)		(0.19 to 1.26)	(0.39 to 1.32)		**(95% CIs)**	(0.15 to 0.79)		(0.30 to 1.15)	(0.22 to 0.64)	

^Sample sizes reported in this table refer to highest number of respondents across the analyses reported for each intervention.

^‡^For the RRS trial, analysis is at the village summary level. We also report number of study participants for consistency with other studies.

^§^No anxiety result is reported for the analysis on both men and women, because in no study was anxiety measured at both baseline and end line for both groups.

*The Indashyikirwa community study employed 2 repeated cross-section surveys of random household samples (6). We report the average number of unique individuals in each survey round for the adjusted odds ratio.

**Measured at 12 months postbaseline. The DMC at Johns Hopkins University’s IRB recommended the study be interrupted and the intervention delivered to study participants in the control arm, following evidence of effectiveness at 12 months.

DMC, Data Monitoring Committee; IPV, intimate partner violence; IRB, Institutional Review Board; RRS, Rural Response Systems; RTP, Right To Play; SSCF, Stepping Stones and Creating Futures; VATU, Violence and Alcohol Treatment.

Table 3 presents the summary costs for each intervention. Provider costs per participant range from $3.95 for the community intervention in Ghana to $1,324 for one-on-one counselling in Zambia in the implementation in a research setting.

**Table 3:** Annuitised intervention costs^±^ (2018 US$).

	RRS	IMpower^±^^§^	RTP^§^	Indashyikirwa	SSCF	VATU^±^
	Ghana	Kenya	Pakistan	Rwanda	South Africa	Zambia
***Provider perspective***						
***Research setting***						
**Total incremental cost**	$291,215	$130,065	$355,722	$2,905,087	$216,237	$325,626
**Potential recipients at baseline**	73,759	11,444	15,968	141,733	677	246
**Incremental cost per capita**	$4	$11	$22	$20	$319	$1,324
***Scale-up scenario 1***						
**Total incremental cost**	$33,736,232	$38,807,824	$175,185,264	$57,219,812	$108,667,408	**
**Potential number of participants**	12,210,626	1,832,742	4,057,000	4,563,077	490,350	**
**Incremental cost per capita**	$3	$21	$43	$13	$222	**
***Scale-up scenario 2***						
**Total incremental cost**	$37,729,012	$38,807,824	$175,564,656	$60,143,504	$118,814,576	**
**Potential number of participants**	12,210,626	1,832,742	4,057,000	4,563,077	490,350	**
**Incremental cost per capita**	$3	$21	$43	$13	$242	**
***Societal perspective***						
***Research setting***						
**Total incremental cost**	−$26,294,262	$130,065	$355,722	$2,905,440	$1,224,630	**
**Potential recipients at baseline**	73,759	11,444	15,968	141,733	677	**
**Incremental cost per capita**	−$356	$11	$22	$20	$1,809	**
***Scale-up scenario 1***						
**Total incremental cost**	−$4,367,426,048	$38,807,824	$175,185,264	$57,231,172	$839,044,672	**
**Potential number of participants**	12,210,626	1,832,742	4,057,000	4,563,077	490,350	**
**Incremental cost per capita**	−$358	$21	$43	$13	$1,711	**
***Scale-up scenario 2***						
**Total incremental cost**	−$4,363,433,472	$38,807,824	$175,564,656	$60,154,864	$849,191,872	**
**Potential number of participants**	12,210,626	1,832,742	4,057,000	4,563,077	490,350	**
**Incremental cost per capita**	−$357	$21	$43	$13	$1,732	**

Note: Table 3 reports annuitised costs for each intervention, i.e., equivalent annual costs obtained by spreading initial investment over the course of its useful life using standard tables (see Ferrari and colleagues [26] for methodological details). The provider perspective includes costs of adaptation and delivery only. Societal perspective also includes interventions’ economic impact for participants, where this is available (South Africa, Rwanda, and Ghana). Research setting report costs incurred during trial period. Scale-up scenarios report resource requirements for implementation at national scale, accounting for fixed and variable costs and intervention modifications. Scenario 1 includes changes in inputs (e.g., employing schools’ teachers to deliver the intervention, instead of specialised trainers) and modifications (e.g., reductions in number of sessions or training time for trainers); scenario 2 only includes changes in inputs, with no modification to intervention delivery model. Total incremental cost is the total annuitised cost of delivering the intervention at scale. Potential number of participants in the research setting is the number of participants enrolled in the intervention at baseline for group-based interventions and the number of pupils or community members for school-based or community-based interventions, respectively; in the scale-up scenarios, it is the number of individuals in the target population at the national level. Incremental cost per capita is the ratio of total incremental costs over the potential number of participants in each scenario. For more details, see Torres-Rueda and colleagues [25].

^±^Cost per participant is computed over total participants except for VATU and IMpower. For VATU, only costs and participant numbers for adults are considered, because the children were excluded from the main study. For IMpower, only girls are considered, because the boys were excluded from the main study. Total incremental costs of delivery to female participants are computed pro rata: Intervention delivery processes did not change by gender of participants. Incremental costs presented here from IMpower are net of the cost of delivering a government mandated session in control schools. These calculations imply that the incremental cost per capita for females differs slightly from per capita costs for the entire sample presented in the costing paper. For details on the cost analysis, see Torres-Rueda and colleagues [25].

**Data not provided by study.

^§^These interventions are offered to children, and no economic impact is measured on this population, given the 12-month time frame of the study. This explains why, for these interventions, provider and societal costs are the same.

RRS, Rural Response Systems; RTP, Right To Play; SSCF, Stepping Stones and Creating Futures; VATU, Violence and Alcohol Treatment.

**Fig 1:**
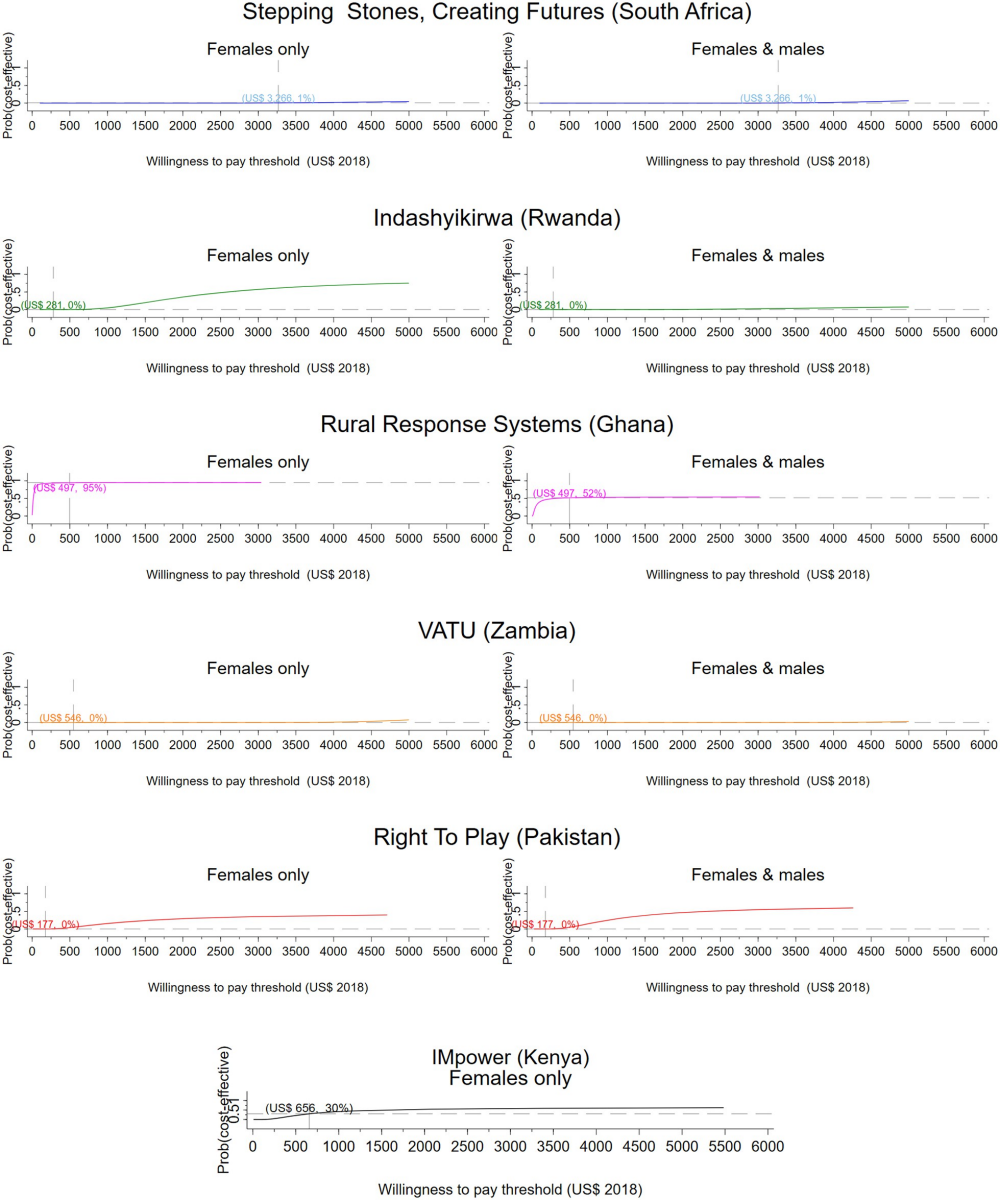
Research setting, provider perspective. CEACs illustrating the probability that the intervention is effective for a range of thresholds. Dashed vertical line: country-specific opportunity cost threshold; dashed horizontal line: probability that the intervention is cost-effective at the country-specific threshold, given the cost per DALY averted by the intervention. CEAC, cost-effectiveness acceptability curve; DALY, disability-adjusted life year; VATU, Violence and Alcohol Treatment.

**Fig 2:**
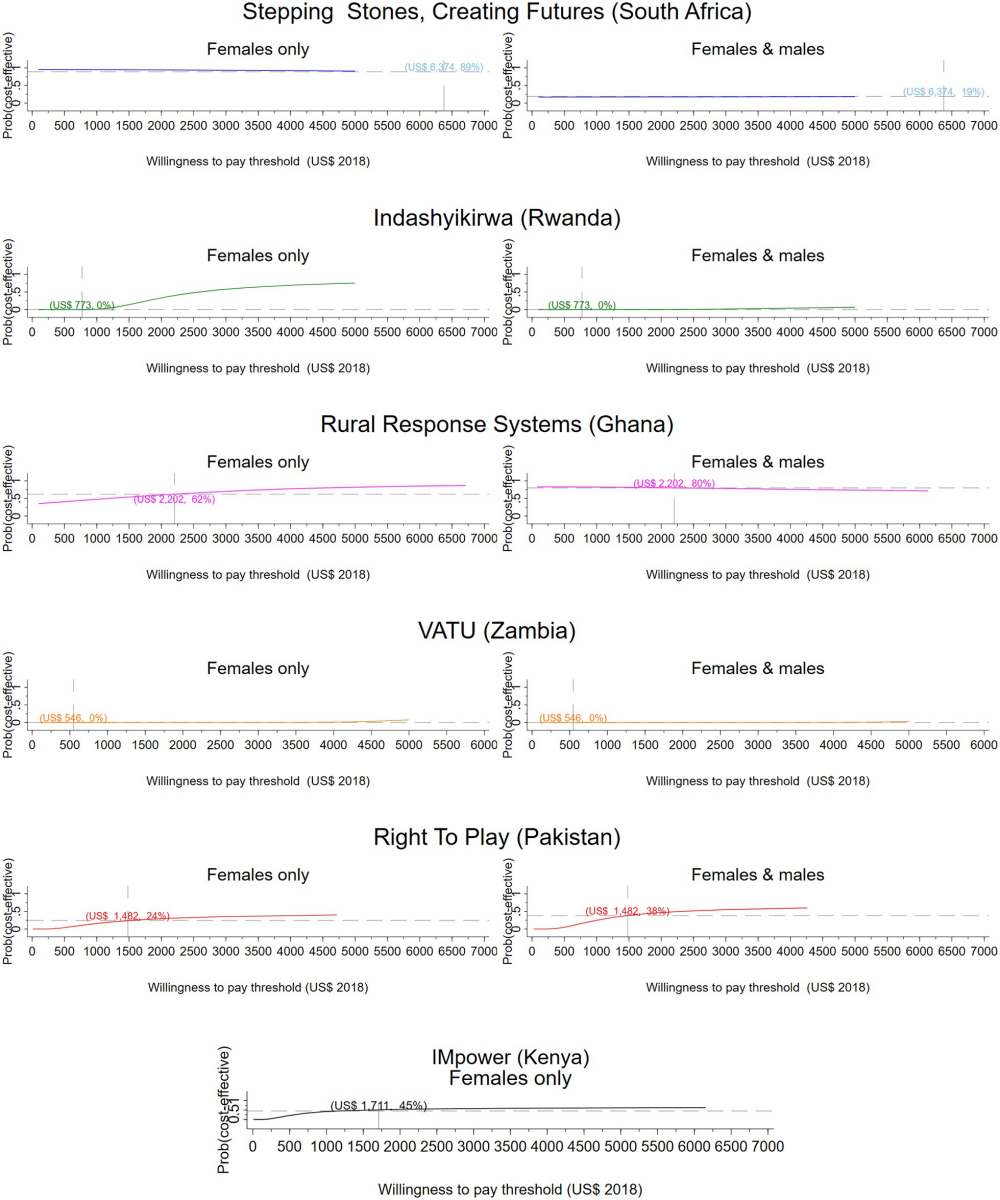
Research setting, societal perspective. CEACs illustrating the probability that the intervention is effective for a range of thresholds. Dashed vertical line: country-specific opportunity cost threshold; dashed horizontal line: probability that the intervention is cost-effective at the country-specific threshold, given the cost per DALY averted by the intervention. CEAC, cost-effectiveness acceptability curve; DALY, disability-adjusted life year; VATU, Violence and Alcohol Treatment.

From a societal perspective, per participant results range from a net saving of $356 in Ghana to a net cost of $1,809 in South Africa (Table 4). Table 4 presents the incremental cost per DALY averted for men and women and women only respectively. Figs 1 and 2 present CEACs showing the probability of the intervention being cost-effective under different decision rules (see Figs G and H in S1 Appendix for the corresponding bootstrapped cost and effect pairs).

**Table 4:** Cost-effectiveness*.

	RRS		IMpower^±^^§^		RTP^§^		Indashyikirwa		SSCF		VATU^±^	
	Ghana		Kenya		Pakistan		Rwanda		South Africa		Zambia	
	Estimate	95% CIs	Estimate	95% CIs	Estimate	95% CIs	Estimate	95% CIs	Estimate	95% CIs	Estimate	95% CIs
**All**												
***Provider perspective***												
**IPV-free person-years gained**	2,431.24	(−839.56 to 5,702.03)					−21,251.17	(−51,976.21 to 9,473.87)	31.42	(−0.34 to 63.17)	264.27	(59.05 to 469.49)
**Peer victimisation free person-years gained**					6,373.59	(−13,338.86 to 26,053.19)						
**DALYs averted during the study period**	809.59	(−14,027.17 to 15,646.34)			152.95	(−372.56 to 678.46)	−330.11	(−1,508.45 to 848.22)	11.86	(−14.20 to 37.91)	34.50	(6.43 to 62.58)
**DALYs averted per 1,000 participants**	10.98	(−190.18 to 212.13)			9.58	(−23.33 to 42.49)	−2.33	(−10.64 to 5.98)	17.51	(−20.97 to 56.00)	140.25	(26.13 to 254.37)
**DALYs averted per participant**	0.01	(−0.19 to 0.21)			0.01	(−0.02 to 0.04)	0.00	(−0.01 to 0.01)	0.02	(−0.02 to 0.06)	0.14	(0.03 to 0.25)
**Provider cost per capita**	$3.9				$22		$20		$319		$1,324	
**Incremental cost per DALY averted**	$360				$2,326		−$8,800		$18,239		$9,438	
**Opportunity cost threshold**	$497				$177		$281		$3,266		$546	
**Probability cost-effective**	52%				0%		0%		0%		0%	
***Societal perspective***												
**Economic impact per capita**	$360	(−369.81 to 1,090.69)					−$.0025	(−0.00 to −0.00)	−$1,490	(−5,013.90 to 2,034.89)	**	**
**Net cost**	−$356				$22		$20		$1,809		**	**
**Incremental cost per DALY averted**	−$32,479				$2,326		−$8,801		$103,292		**	**
**GDP per capita threshold**	$2,202				$1,482		$773		$6,374		**	**
**Probability cost-effective**	80%				38%		0%		17%		**	**
**Females only**												
***Provider perspective***												
**IPV-free person-years gained**	1,162.72	(−564.00 to 2,889.45)	−97.28	(−502.21 to 307.66)			−15,468.78	(−38,359.63 to 7,422.06)	5.79	(−21.01 to 32.58)	20.28	(9.00 to 31.57)
**Peer victimisation free person-years gained**					6,193.62	(−3,416.82 to 15,792.75)						
**DALYs averted during the study period**	2,724.71	(−456.48 to 5,905.90)	89.96	(−293.14 to 473.06)	−5.50	(−359.40 to 348.40)	562.40	(−199.52 to 1,324.32)	−1.38	(−21.43 to 18.68)	21.41	(7.70 to 35.13)
**DALYs averted per 1,000 participants**	76.14	(−12.76 to 165.04)	7.86	(−25.62 to 41.34)	−0.66	(−43.29 to 41.97)	7.80	(−2.77 to 18.37)	−4.06	(−63.21 to 55.09)	174.09	(62.59 to 285.59)
**DALYs averted per participant**	0.08	(−0.01 to 0.17)	0.01	(−0.03 to 0.04)	−0.00	(−0.04 to 0.04)	0.01	(−0.00 to 0.02)	−0.00	(−0.06 to 0.06)	0.17	(0.06 to 0.29)
**Provider cost per capita**	$3.9		$11		$22		$20		$319		$1,324	
**Incremental cost per DALY averted**	$52		$1,446		−$33,614		$2,629		−$78,710		$7,603	
**Opportunity cost threshold**	$497		$656		$177		$281		$3,266		$546	
**Probability cost-effective**	95%		30%		0%		0%		0%		0%	
***Societal perspective***												
**Economic impact per capita**	−$83	(−520.78 to 354.46)					−$.0054	(−0.01 to −0.00)	$615	(188.25 to 1,041.14)	**	**
**Net cost**	$87		$11		$22		$21		−$295		**	**
**Incremental cost per DALY averted**	$1,144		$1,446		−$33,614		$2,630		$72,767		**	**
**GDP per capita threshold**	$2,202		$1,711		$1,482		$773		$6,374		**	**
**Probability cost-effective**	62%		52%		24%		0%		82%		**	**

Note: Table 4 reports results for all intervention participants (females and males) and for females only. Intervention effects are reported in natural units, IPV or peer victimisation, and in DALYs averted. We also report DALYs averted during the study period to illustrate total health impact, according to available data. DALYs averted per 1,000 participants are a commonly used standardised statistic. Provider costs are the costs of delivering the intervention only. Societal costs include the economic impact interventions had on participants (not applicable to IMpower and RTP, which targeted children or early adolescents in schools). The opportunity cost threshold is the cost per DALY of the least cost-effective intervention offered by the healthcare system of each country or the system’s marginal productivity. The 1xGDP per capita threshold reflects WHO recommendations to determine cost-effectiveness. Probability cost-effective is the likelihood the intervention is cost-effective at the designated threshold. This likelihood is computed using a PSA, where costs and effects are made to vary simultaneously to test the robustness of the reported ICER.

*Intention-to-treat estimates: Totals are calculated with reference to all participants enrolled at baseline.

**Data not provided by study.

^§^See cost-effectiveness plane (Fig G in S1 Appendix).

DALY, disability-adjusted life year; GDP, gross domestic product; ICER, incremental cost-effectiveness ratio; IPV, intimate partner violence; PSA, probabilistic sensitivity analysis; RRS, Rural Response Systems; SSCF, Stepping Stones and Creating Futures; VATU, Violence and Alcohol Treatment.

From a health sector perspective, the Ghanaian intervention has a 52% probability of being cost-effective for men and women jointly and a 95% probability of being cost-effective for women only compared to Ghana’s opportunity cost threshold (Table 4, Fig 1). The intervention averts 1 DALY for US$52 for female participants and for US$360 when the health impact on men is also considered. The intervention has an 80% probability of cost-effectiveness when considering a societal perspective for both men and women. For women only, the Ghana intervention records a 95% probability of cost-effectiveness from a health sector perspective and a 62% probability from a societal perspective. The Kenyan intervention (IMpower) has 30% probability of being cost-effective, improving the mental health of school girls generally (Table 4, Fig 1). The intervention in Pakistan has a 0% probability of being cost-effective from a health sector perspective, but from a societal perspective, the intervention is 38% likely to be cost-effective. The South African intervention has no impact on DALYs for women but provides an economic benefit. When men are included, the economic impact on participants is lower than among controls, and DALYs averted are marginally larger. The intervention has a 17% probability of being cost-effective from a societal perspective. None of the analyses for the intervention in Zambia (VATU) found the intervention to be cost-effective from a provider perspective.

**Fig 3:**
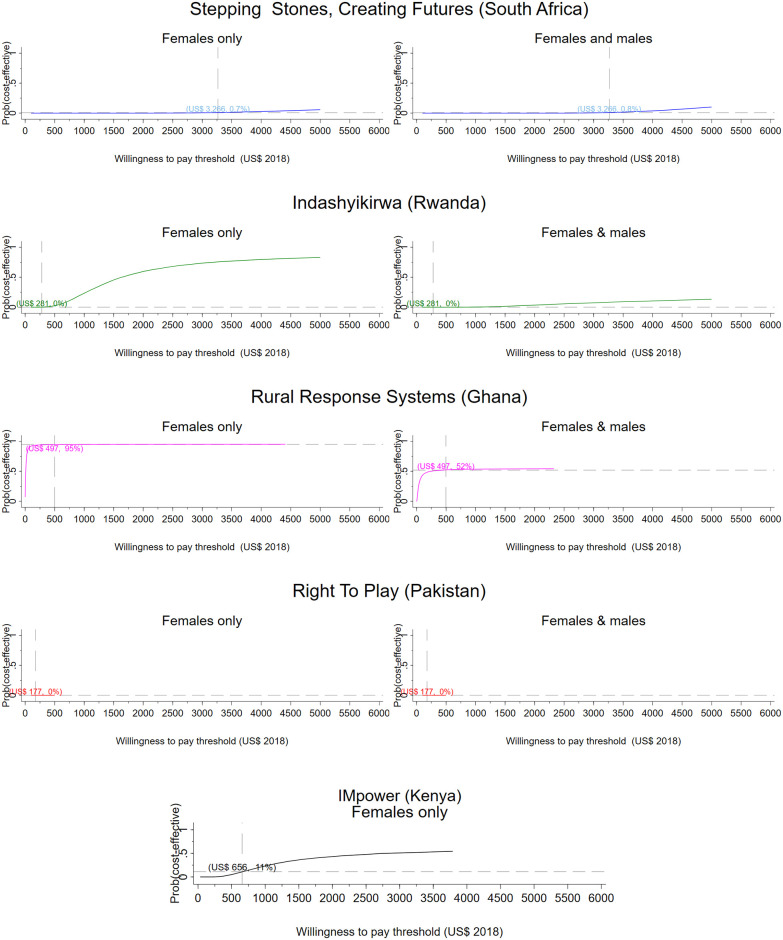
Scale-up scenario 1, provider perspective. CEACs illustrating the probability that the intervention is effective for a range of thresholds. Dashed vertical line: country-specific opportunity cost threshold; dashed horizontal line: probability that the intervention is cost-effective at the country-specific threshold, given the cost per DALY averted by the intervention. CEAC, cost-effectiveness acceptability curve; DALY, disability-adjusted life year.

**Fig 4:**
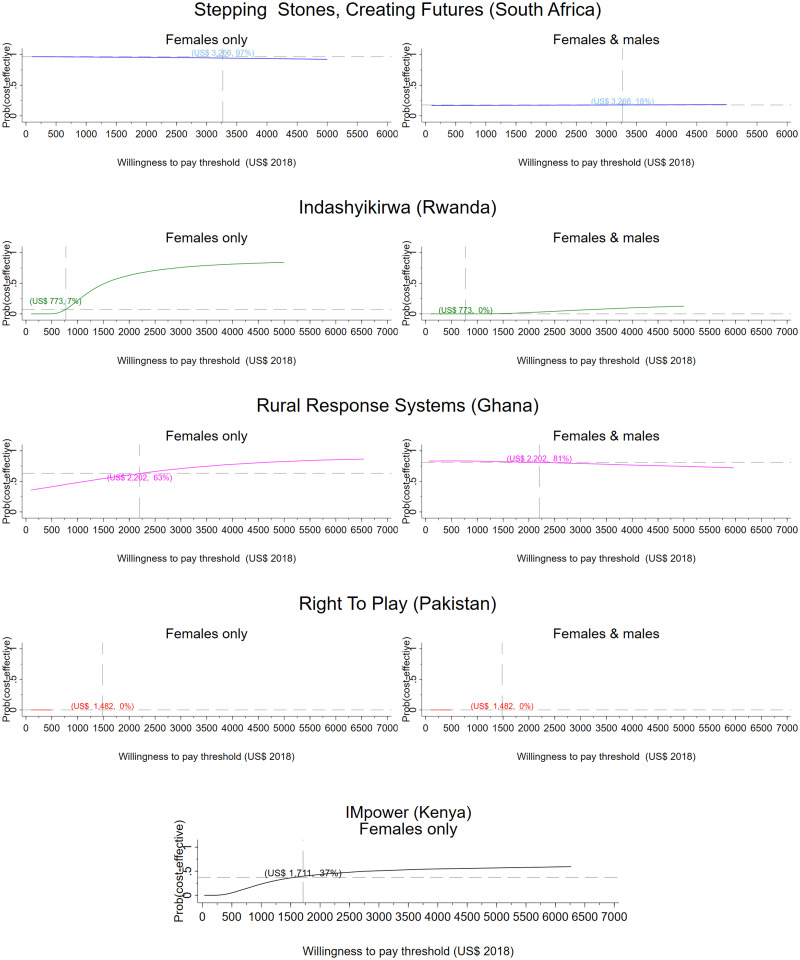
Scale-up scenario 1, societal perspective. CEACs illustrating the probability that the intervention is effective for a range of thresholds. Dashed vertical line: country-specific opportunity cost threshold; dashed horizontal line: probability that the intervention is cost-effective at the country-specific threshold, given the cost per DALY averted by the intervention. CEAC, cost-effectiveness acceptability curve; DALY, disability-adjusted life year.

**Fig 5:**
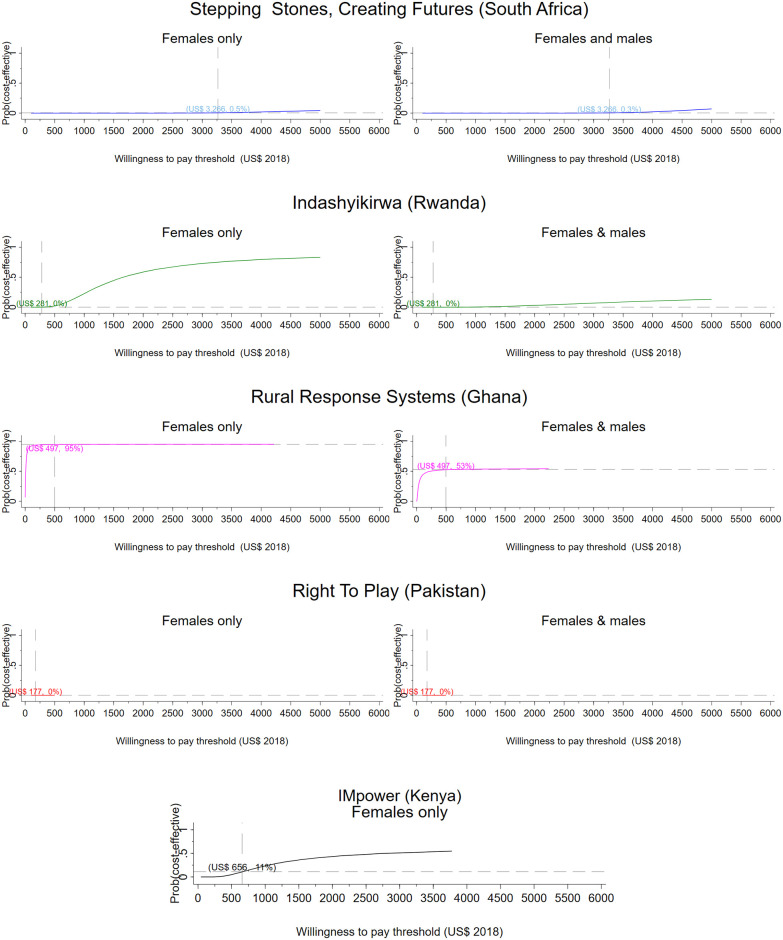
Scale-up scenario 2, provider perspective. CEACs illustrating the probability that the intervention is effective for a range of thresholds. Dashed vertical line: country-specific opportunity cost threshold; dashed horizontal line: probability that the intervention is cost-effective at the country-specific threshold, given the cost per DALY averted by the intervention. DALY, disability-adjusted life year.

**Fig 6:**
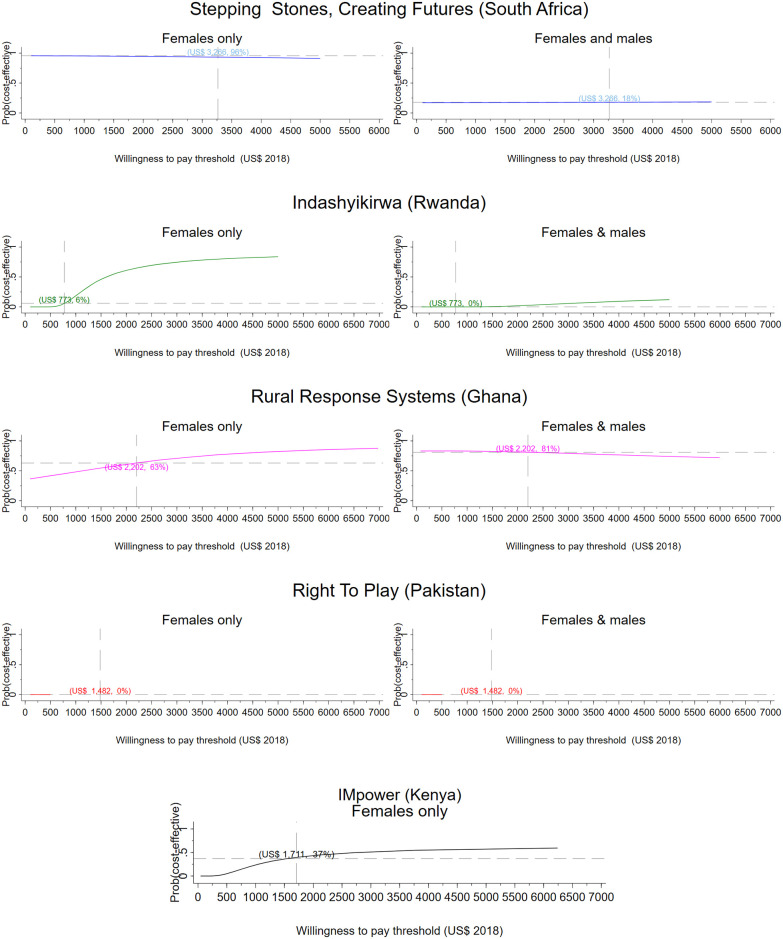
Scale-up scenario 2, societal perspective. CEACs illustrating the probability that the intervention is effective for a range of thresholds. Dashed vertical line: country-specific opportunity cost threshold; dashed horizontal line: probability that the intervention is cost-effective at the country-specific threshold, given the cost per DALY averted by the intervention. DALY, disability-adjusted life year.

In scale-up scenarios, patterns of cost-effectiveness remain similar compared to the research setting apart from VATU, for which we do not have scale-up data (Figs 3–6). In Ghana, probability of cost-effectiveness is unchanged between research setting and scale-up from a health sector perspective (95% for females; 52% for females and males) and from a societal perspective (females: 63%; females and males: 81%). In South Africa, too, probabilities of cost-effectiveness increase markedly for females from the societal perspective, compared to the health sector perspective (from 1% to 90% in scenario 1; from 1% to 89% in scenario 2). Patterns remain unchanged for Kenya, and Pakistan is not cost-effective in any of the scale-up scenarios. None of the analyses for the full intervention in Rwanda found it to be cost-effective, although both scale-up scenarios record a 6 to 7 percentage point increase in probability for females from a societal perspective (from 0% in research setting to 6% to 7% at scale-up).

For all interventions, our sensitivity analyses around IPV-attributable DALYs (Figs B–D in S1 Appendix) suggest that the interventions are less cost-effective from a health sector perspective than when using DALYs directly computed from health sequelae. The subpopulation analysis for the Rwanda Couples intervention finds a 53% and 100% probability of being cost-effective for a health sector and societal perspective respectively (Figs E and F in S1 Appendix).

Table G in S1 Appendix reports cost per year free from violence for all interventions. The impact inventories report on outcomes relevant to the labour and financial, social services and protection, education, and legal sectors of the economy for each intervention (Figs I–K in S1 Appendix).

## Discussion

We present here the first substantial multicountry body of evidence on the impact and cost-effectiveness of preventing VAWG, to our knowledge. We assess whether interventions are affordable, given their respective countries’ health budgets. We do this by establishing cost-effectiveness of each intervention against an opportunity cost threshold based on econometric estimates of actual health expenditure by each country’s health budget holders [20]. Comparing our cost per DALY averted estimates to these opportunity cost thresholds determines whether each intervention should be afforded within its country’s existing health budget by only finding an intervention cost-effective if it maximises population health within the observed budget. We find that nearly all the interventions evaluated demonstrate a positive impact on health and economic well-being (and other outcomes). For a limited number of prevention interventions, the evidence suggested that funding should be provided by the health sector in LMICs. For others, the case is more challenging, because the probability of cost-effectiveness remains below 50% in all scenarios. However, a health sector perspective is the narrowest of frames for justifying investment in violence prevention and preventing human rights violations. In this context, our findings suggest further investment in prevention intervention design, and exploring platforms for broader cofunding from other sectors. Specifically, we find that the probability that interventions are cost-effective for female beneficiaries increases for most interventions when the societal perspective is considered, supporting a cross-sectoral approach to VAWG prevention with a focus on women.

In terms of impact, interventions showed the strongest results in the areas for which they were primarily designed and this design choice fundamentally impacts their potential cost-effectiveness. The South African intervention, which focused on improving participants’ livelihood strategies, records the largest economic impact among female beneficiaries, in addition to reducing men’s perpetration, but may need a more direct emphasis on VAWG to reduce women’s exposure (Table 2). In Ghana, the larger economic benefits among male compared to female beneficiaries exposure (Table 4) may reflect the societal gendered economic patterns. More research is needed to confirm that this is an intervention effect, given that the intervention has no direct economic component. The intervention in Ghana was well established, focused on VAWG, low cost, and embedded in communities; it achieved moderate impact on VAWG [5] and DALYs averted. The Indashyikirwa Couple’s (Rwanda) (Table E in S1 Appendix) and VATU (Zambia) couples’ interventions are the 2 interventions that achieved a sizeable and statistically significant reduction in VAWG exposure and perpetration. The direct focus on VAWG in Rwanda’s VSLA+ component may in part explain the cost-effective approach to violence prevention. While the results are less clear, it should be noted that the cost-effectiveness of the VATU intervention is in line with other mental health interventions in sub-Saharan Africa [43]. One way of improving this outcome and help improve the cost-effectiveness of mental health services overall may be to further integrate mental health services, training, or support with other health services and other VAWG prevention platforms or interventions. The Kenyan intervention, focused on self-defense, delivered health benefits, but showed no effect on rape, the main study outcome (not reported here), nor on physical violence from an intimate partner (Table 2). However, this is an area with substantial measurement challenges, such as girls reporting having been raped but not having had sexual intercourse and the fact that there were fewer field staff at the third cohort interview than previously assisting with this self-completed questionnaire. Inconsistent reports of having had sex have been discussed by other authors from Kenya [44], and, hence, these results may be less conclusive than other results presented here.

When improving the design of less cost-effective interventions, implementers need to think about how delivery platforms or population characteristics may impact cost-effectiveness. Some populations may simply be more expensive to reach, and, here, cost-effectiveness needs to be traded off with equity considerations. For example, delivering interventions through schools has the advantage of reducing costs of reaching children. However, it limits prevention to school attendees, compared to a community intervention that may reach more vulnerable youth at a lower cost per participant. Likewise, microfinance-plus interventions may be cost-effective, but only reach actual microfinance clients, who often account for 10% to 36% of eligible microfinance clients in a village [45,46].

Our findings also suggest that long-established interventions may have performed better in terms of cost-effectiveness thanks to very low costs, even in the face of considerable uncertainty in impact. Newly introduced community-based interventions, although potentially cost-effective, need time to allow for local adaptation if they are to achieve impact at the population level. Two years of implementation was sufficient for established community-based interventions that have been refined over time, such as the intervention in Ghana, but the experience of Rwanda suggests that it may take 5 to 7 years to design, adapt, and introduce new prevention interventions [47].

Comparisons with the cost-effectiveness of similar interventions are limited, because of the dearth of evidence. The only direct comparison between Stepping Stones and Creating Futures (SSCF) and IMAGE [23] shows that the IMAGE intervention is more cost-effective from a provider perspective. However, community and school-based What Works interventions tend to compare favourably in terms of cost per year free from violence compared to previous interventions (Table G in S1 Appendix).

We find that accounting for economic impact alone can have substantial impact on intervention cost-effectiveness. Interventions with immediate economic impacts, but no VAWG reduction, may look less favourable in the short term, but may provide more sustained benefits if well designed and supported in the longer term. We therefore encourage practitioners to think carefully through the economic implications of their interventions for beneficiaries not only on account of the existing evidence of the links between economic outcomes and IPV, but also of the implications of economic outcomes for interventions’ cost-effectiveness. Likewise, where effective, interventions with adolescents may intuitively produce sustained behaviour change over many years.

We report a substantial variation in cost per participant of the different modes of VAWG prevention [25]. The costs of VAWG prevention are largely driven by the intensity of contact with participants and the types of human resource capacity required. While we found that one-to-one contact with well-trained counsellors can result in substantial health improvement in individuals, when compared to community focused or group interventions, this model was costlier. Future work should explore whether integrating VAWG support (and prevention) in general mental health services reduces costs at scale. School-based interventions, even if with a health benefit, are unlikely to be funded by the health sector, and further attention needs to be paid to models of cofinancing their costs within the education sector. This may also require demonstrating improvements in educational outcomes. Community interventions can be delivered at relatively low cost, and livelihood interventions, while costly to providers, can be justified based on their overall societal cost savings. For all interventions, we need better understanding of how costs may change at scale.

Results from our sensitivity analyses do not alter our general conclusions (see also S1 Appendix 1, p. 9–10). However, our study has several limitations. First, economic impact data from Zambia were unavailable, and no economic impact data or reliable educational data were collected for children. Second, we did not have the resources to collect data on study participants’ utilisation of health and social services. Third, some of our cost data could only be collected retrospectively [25]. Fourth, where men are committing perpetration outside of the female study population, we may be underestimating overall health impact.

Our DALY estimation has 5 main limitations. First, we assume no health effects beyond the trial period, because there are no estimates of long-term effects of VAWG prevention in the literature, making the modelling of such effects unreliable. Second, our effectiveness data were obtained from RCTs and may be an upper bound estimate of intervention efficacy, compared to real-world effectiveness in a nonresearch setting. However, it is also conceivable that the full impact of interventions that seek to change deep-seated gender norms and behaviours accrues over a time frame longer than 2 years, in which case our estimates could be lower bound estimates. Future studies should investigate the medium and long-term effects of IPV prevention interventions. Third, we assumed no mortality impact from any of the conditions included in the DALY measure. This is consistent with burden of disease estimations, but contradicts other findings [48], and may bias our estimates downward. Fourth, we could only compute binary indicators of cases for the health conditions included in the DALY. Distinguishing between severe, moderate, or mild conditions would allow the application of more accurate disability weights and reduce measurement bias. Fifth, our DALYs included only 2 to 3 of the 16 known potential health consequences of IPV exposure (Fig A in S1 Appendix), underestimating interventions’ health impact. Specifically, although several studies measured post-traumatic stress disorder (PTSD), there is no disability weight for PTSD hence our DALY estimates exclude this potentially important health impact.

When interpreting our findings, it is important to note that this was not a single study across multiple sites, and each RCT measured different sets of outcomes; it is therefore not appropriate to directly compare across the RCTs. Future research should compare the incremental cost-effectiveness of all approaches comparatively for single populations. To enable comparisons going forward, there is an urgent need for further standardisation of outcomes measurement in the VAWG prevention field. Identification and consistent measurement of health outcomes to be used to generate DALYs will allow for comparable and exhaustive estimates of health impact. Moreover, capturing the full range of impact in VAWG prevention RCTs, including economic and social impact, can also be used to value interventions for other sectors and would further strengthen any case for investment in VAWG prevention [49]. Finally, while these findings add to the emerging evidence on the cost-effectiveness of VAWG prevention, they should not be generalised beyond the populations targeted by the interventions. Further research on the context-specific drivers of both cost and impact across settings is required, with both cost and impact monitored as part of implementation.

## Conclusions

Preventing VAWG is a moral imperative. However, there is also an urgent need to demonstrate both its impact and cost-effectiveness given that competition for health and other funding is intense. Our study provides a major body of evidence on the cost-effectiveness of VAWG prevention in LMICs. We provide robust findings on the cost-effectiveness of different VAWG prevention interventions, highlighting the need for further intervention development and research into new interventions. Findings suggest that investment in VAWG can improve population health even in low-resource settings and even when only observed impact on IPV, rather than lifetime projections, is considered. Overall, interventions are more likely to be cost-effective for women and girls, although it should be borne in mind that these effects are likely a combination of direct effects on females and indirect effects through men’s participation. The policy implications of our findings are that IPV prevention is likely a good investment from a health sector perspective and is also likely to improve populations’ overall well-being from a societal perspective. The valuation of the full range of outcomes of these interventions is a priority for policy and research to obtain a comprehensive picture of the cost-effectiveness of IPV prevention. Our findings present a major step forward towards this aim and in justifying the scaled up and sustained response needed to meet SDG5, to more effectively address VAWG globally.

## Supporting information

S1 Appendix Detailed information on methods and additional analyses, including sensitivity analyses, cost per year free from violence, and impact inventories. (DOCX)

S1 Checklist Locates the manuscript sections containing the information required by the Second Panel on Cost-Effectiveness in Health and Medicine [29]. (DOCX)

S1 Translations Co-authors’ translations of paper abstract and author summary into the languages of interventions’ beneficiaries and their policy makers. (PDF)

## Abbreviations

BAI-Y: Beck Youth Anxiety Inventory
BDI-Y: Beck Youth Depression Inventory
CDI: Child Depression Inventory
CEAC: cost-effectiveness acceptability curve
CTS: Conflict Tactics Scale
DALY: disability-adjusted life year
DFID: Department for International Development
ICER: incremental cost-effectiveness ratio
IHME: Institute of Health Metrics and Evaluation
IMAGE: Intervention with Microfinance for AIDS and Gender Equity
IPV: intimate partner violence
LMIC: low- and middle-income country
LSHTM: London School of Hygiene & Tropical Medicine
ODA: official development assistance
PSA: probabilistic sensitivity analysis
PTSD: post-traumatic stress disorder
PVS: Peer Victimization Scale
RCT: randomised controlled trial
RRS: Rural Response Systems
RTP: Right To Play
RWAMREC: Rwanda Men’s Resource Centre
RWN: Rwanda Women’s Network
SDG5: sustainable development goal five
SSCF: Stepping Stones and Creating Futures
UBL: Unite for a Better Life
VATU: Violence and Alcohol Treatment
VAWG: violence against women and girls
VSLA: village savings and loan association
YSR: Youth Self-Report
