# Supplementary material for: Prevention of violence against women and girls: A cost-effectiveness study across 6 low- and middle-income countries
Source: PLoS Med. 2022 Mar 24;19(3):e1003827. doi: 10.1371/journal.pmed.1003827 (PMC8946747; doi:10.1371/journal.pmed.1003827)
Supplement: S1 Appendix — (DOCX) [file pmed.1003827.s001.docx]

Appendix

This appendix reports further details on methods, including intervention selection, cost data collection and analysis and distributional assumptions for the probabilistic sensitivity analysis to account for uncertainty across all analyses reported. It also reports results from the sensitivity analyses we did to reflect how results change at scale and how they are affected if we use IPV-based DALYs, instead of all-cause morbidity DALYs. It also reports cost-effectiveness for the initial phase of the Indashyikirwa intervention which was only delivered to VSLA clients in small groups, to test whether the intervention was cost-effective for this sub-group.

# Methods

## Costing

The paragraphs below under the heading 'Costing' are an excerpt from our cost-synthesis paper (1). They illustrate the methods we followed to determine the incremental costs of delivery for the main analysis from a provider perspective in a research setting, and all other scenarios. Please note that references’ numbering in the original article (1) has been modified, to comply with journal formatting standards.

To ensure comparability across intervention types, we developed a standardised methodology and a set of guidelines for the economic evaluation of complex (i.e. multi-component and/or multi-platform) programmes designed to prevent VAWG in LMICs (2,3) Our methodology follows best practice established in the Global Health Costing Consortium Reference Case (4) and is consistent with the Second US Panel on Cost-effectiveness in Health and Medicine (24) and the CHEERS guidelines (5), as well as with DfID’s ‘value for money’ framework for the assessment of economy, efficiency, effectiveness and equity of its programmes (6).
We estimated costs of the start-up and implementation phases. The start-up phase was divided into three sub-phases: (i) intervention development, defined as the period when the curriculum or manual of activities was initially designed, (ii) adaptation, defined as the process of making the curriculum or manual specific to the target population, and (iii) set up, defined as the period of community entry and training of frontline staff. The implementation phase spanned the time between when the first and last clients received the intervention.
All three sub-phases were costed for IMpower/SOS (Kenya), SSCF (South Africa) and VATU (Zambia). For RTP (Pakistan) and Indashyikirwa (Rwanda) only set-up and adaption costs were included. RTP’s development sub-phase started in 2008 and resource use data were not available; it therefore could not be costed. Indashyikirwa is a multiple-component intervention, partially based on SASA! and Journeys of Transformation (previously-developed interventions) but with some newly-developed components. Given the complexity of Indashyikirwa’s start-up phase, it was difficult to disaggregate resources used strictly to develop new components versus adaptation of existing components. For analytical simplicity we classified all costs as adaptation (vis-à-vis development) and tested this assumption in sensitivity analysis. For RRS (Ghana) only the set-up sub-phase was costed. The intervention was developed over 15 years prior to the pilot and no financial records were available. Further, RRS did not go through an adaptation phase before the implementation.
A full financial and economic micro-costing was carried out for each intervention. Data on resource utilisation were obtained through structured interviews, review of financial records, monitoring and evaluations data and travel logbooks. For financial costs, purchase prices were used, with replacement prices for capital goods, commonly available from project records. Costs were separated by phase and sub-phase. Most costs were calculated through a bottom-up approach (measuring quantities in a granular manner at the activity and sub-activity level). When unfeasible, a top-down approach (dividing overall costs by number of outputs) was used to calculate some administration and management costs.
Costs were broken down by input type (e.g. supplies, staff salaries, or utilities) and allocated to a number of activities common across interventions (management, administration, technical support, travel, maintenance/cleaning, and other) and intervention-specific core activities (e.g. community sensitisation or counselling). Research costs were excluded. Implementing organisation monitoring and evaluation costs were included as these would be expected to be incurred in routine service delivery. Allocations between activities and sub-activities were based on staff time use and financial and programmatic records (2,3)
Staff time use was collected through structured interviews, timesheet review and direct observation. When volunteers did not receive a stipend, their time was valued by applying a replacement value determined as the salary of the lowest-tier health worker in each setting (7). Cost data were disaggregated by delivery site where possible.
Costs were collected in the currency and year in which they were incurred. They were converted to 2016 values using the World Bank GDP deflator and to US Dollars using average annual exchange rates (8). Capital costs were annuitized over the expected life of each item and a standard 3% discount rate was applied. Start-up costs were treated as a capital item. The useful life of the different sub-phases of the start-up period was estimated to be 10 years for development, 10 years for adaptation, and 5 years for set up. The durations for the development and adaptation sub-phases were estimated by eliciting the expert opinion of senior staff members of the implementing organisations and tested in our sensitivity analysis. For the set up sub-phase we assumed the average length of the political cycle, as set up activities often require political buy-in from leaders in the community and therefore may need to be repeated at the start of each political cycle.

### Descriptive cost data analysis

We calculated a total cost, as well as cost per phase, and present results by input type. We calculated three common mean costs per outcome across interventions: cost per frontline worker trained, cost per session delivered and cost per beneficiary reached. Mean costs per outcome were calculated by obtaining the total cost of the core activity (e.g. total costs of training frontline staff), including both direct and indirect costs, and dividing by the total number of units (e.g. total number of volunteers trained). Other mean costs per outcome are available from the authors upon request.
The number of beneficiaries was determined with an intention to treat (ITT) approach, in line with methodological best practice for evaluations alongside trials. For workshop-based interventions (SSCF, VATU) this is the number of beneficiaries enrolled at baseline, irrespective of the number of sessions completed. For community- and school-based interventions (RTP, IMpower/SOS, RRS and Indashyikirwa), which were based on the model of social diffusion where community members (either villagers or pupils in school) were exposed directly or indirectly, the number of beneficiaries were defined as the total number of people within the relevant target population in each cluster (i.e. village or school). Indashyikirwa (Rwanda) had an initial workshop-based component with couples that fed into the community-based approach; however, in this paper, we focus on the beneficiaries at the community-level.

### Uncertainty Analysis

To account for uncertainty, we carried out a number of one-way deterministic sensitivity analyses. We examined the sensitivity of costs to the following parameters: staff salaries, replacement value of volunteer labour, working days per year, prices of fuel, useful life of adaptation and set-up costs, and discount rates.

There were discrepancies in perceptions of time use between those reported by staff and those reported by the research team in VATU (Zambia), over which full agreement could not be reached. Due to this extra uncertainty, we carried out an additional one-way deterministic sensitivity analysis varying the four parameters around which there was disagreement: percentage of time spent on research activities, percentage of time spent on management activities, amount of time devoted by supervisors to travel, and the inclusion of a one-day feedback session in the start-up period . Given the difficulties in separating out development and adaptation costs, we also carried out a one-way deterministic sensitivity analysis for Indashyikirwa (Rwanda) to test the effect on total costs of different assumptions on the distribution between development costs versus adaptation costs.

### Scale up Analysis

We modelled the costs at national scale in five out of the six interventions. We excluded VATU (Zambia) given the uncertainty about ‘implementation in a research setting’ costs and non-availability of information to model scale up assumptions.

Cost data from the pilots were used to estimate costs of full-scale intervention delivery at the national level (so called ‘scale up’ costs). Scale up can be conceptualised across different dimensions. The World Health Organization suggests scaling up can be applied to inputs, outputs, outcomes or impact (9). Whilst the ultimate goal of an intervention is to increase impact, this analysis focuses on the scale up of inputs required, which at scale may or may not sustain the effectiveness achieved in the pilots.
An increase in inputs at scale requires increased resources (i.e. increased total costs), but costs are not typically a linear function of the number of beneficiaries reached at scale. As interventions are scaled up some costs remain fixed (i.e. costs that remain constant regardless of the number of outputs) such as the costs of adapting an intervention to a new country, while others should be treated as variable (i.e. costs that vary according to the level of output). Some costs vary as a function of the number of beneficiaries reached (e.g. costs of printing education materials for each beneficiary), while others vary as a function of intermediate outputs, such the number of delivery sites (e.g. costs of training teachers on the intervention in a school).

Moreover, intervention modifications may be necessary when interventions are scaled up from pilot to national levels. Consequently, we consulted with senior members of each implementation team to elicit their expert opinion on the potential scale-up. Specifically, we elicited input on: 1) potential modifications in inputs at scale (e.g. implementer organisation staff delivering components of the intervention during pilot versus local teachers at scale), 2) potential intervention delivery modifications at scale (e.g. increase in field worker supervision), and 3) potential magnitude of scale up according to delivery platform (e.g. 20 middle schools in an urban area in the pilot versus all middle schools in the country). A final list of included modifications, and associated costs, was consequently reviewed and approved by implementers.
A large number of proposed modifications involved changing the staff used to deliver the intervention: in IMpower/SOS (Kenya) service delivery shifted from implementing organisation staff to local school teachers, in Indashyikirwa (Rwanda) training shifted from being led by the implementer organisation staff to community volunteers. There was also a reduction in stipends for frontline staff workers (South Africa, Rwanda and Pakistan), a decrease in supervisory activities (Ghana and South Africa), a reduction in the intensity of frontline worker training in RTP (Pakistan) and a shortening of the curriculum (Rwanda) between the pilot phase and the national scale up. We modelled two scenarios: the first with all suggestions incorporated, and a second, more cautious, scale-up scenario, where we calculated costs at national scale only accounting for potential changes in inputs.

It should be noted that the impact of modelled changes in inputs or intervention delivery on the interventions’ effectiveness is uncertain; they should therefore be considered exploratory and to be monitored carefully at scale.
We excluded intervention development costs at scale and adaptation costs were retained but treated as a fixed cost. Set-up costs, which included costs associated with community entry, stakeholder engagement and training of local frontline staff, would need to be incurred for every new delivery site so were assumed to vary as a function of the number of delivery sites.
Implementation costs were divided between those incurred in programme offices (headquarters or regional offices) and delivery sites. Costs of programme offices were divided into direct and indirect costs. Direct costs (related to core programme delivery, technical assistance and transport) were multiplied by the number of delivery sites at scale and indirect costs (related to management, administration, maintenance and cleaning, and other) were multiplied by the number of projected programme offices at scale. Average implementation costs per delivery site were multiplied by the estimated number of delivery sites at scale.

*Total costs at scale = Adaptation costs + (site set up costs * number of delivery sites) + (programme office indirect
costs * number of programme offices) + (programme office direct costs * number of delivery sites) + (site implementation costs * number of delivery sites)*

To determine the total number of delivery sites at scale for most interventions we divided the total number of beneficiaries at scale by the average number of beneficiaries per site in the pilot phase (in other words we assumed a fixed capacity per site). For IMpower/SOS and RTP, we assumed the number of sites was determined by the total number of public primary schools in Kenya and by the total number of public middle schools in Pakistan.

The level of expansion of the programme offices necessary at scale was determined by treating indirect costs as step costs (i.e. fixed cost until a threshold is crossed), and we assumed costs would remain fixed up to the point where the number of delivery sites tripled. After that point, a duplication in the resources needed for indirect activities was considered necessary, an assumption was tested in sensitivity analysis.
To calculate the number of beneficiaries at scale, we first defined the criteria of inclusion to the target population for each pilot interventions (e.g. age range or employment status). We then calculated the total number of people meeting said criteria and the national level. In the cases of community-based interventions in Ghana and Rwanda this meant all adults age 20-59 in the country: 12,210,626 and 4,976,600 people respectively(10,11). Although these two interventions targeted adults aged 18-60, our age criteria of beneficiaries at scale are different due to data availability. For IMpower/SOS we calculated the total number of children in primary schools in standard grades 5-8 across Kenya, estimated to be 3,311,555 students in 23,584 public primary schools (12). For RTP (Pakistan) we calculated the total number of children in middle schools to be 4,057,000 in 16,928 schools (13). In the case of SSCF, we estimated all unemployed men and women aged 18-30 in informal settlements across South Africa to be 490,350 people(14–16)

## Disability Adjusted Life Years (DALYs)

Our main analysis reports interventions' effect on all-cause morbidity DALYs, using all measured health sequelae in the trial datasets. To arrive at these estimates, we first identify all cases for each health sequela using standard thresholds for each of the mental health (17–20) and alcohol use (21) sequelae, and a 0/1 indicator for drug use. We then take the relevant DALY weight for each health sequela and multiply each case by the relevant DALY weight. DALY weights for all sequelae are added up for each study participant, and estimates of differences in DALYs computed using the main impact model also used to test interventions' effect on the study main outcome. Note that each RCT study collected a subset of these measures, with Ghana, South Africa and Zambia recording the largest amount of sequelae with the same measures (depression, hazardous alcohol use and drug use and the community interventions in Rwanda only recording hazardous alcohol use). We expect this variation in the availability of health measures across studies to reduce the comparability of DALY estimates mapped over sets of different health sequelae.
Table A reports the health consequences (sequelae) we used to compute the DALYs for each intervention. It specifies the measure used in each case, and the relevant threshold to identify a case for each condition and, where possible, the level of severity of the state of ill-health. It also includes a row for the couples sub-group in Rwanda, one of the sensitivity analyses reported below.

**Table A: Health sequelae for DALYs**

|  | **Anxiety** | **Depression** | **Hazardous alcohol use** | **Drug use** |
| --- | --- | --- | --- | --- |
| **Kenya** | Beck's Anxiety Inventory (mild/mod/sev) | Beck's Depression Inventory>=18 | .. | .. |
| **Pakistan** | .. | Child Depression Inventory II (TCDI>75) | .. | .. |
| **Ghana** | .. | CESD-20>=22 | AUDIT-C>=3 (females); AUDIT-C>=4 (males) | Any drug in past 12 months |
| **Rwanda** | .. | .. | AUDIT-C>=3 (females); AUDIT-C>=4 (males) | .. |
| **Rwanda (couples only)** |  | CESD-10>=16 | AUDIT-C>=3 (females); AUDIT-C>=4 (males) |  |
| **South Africa** | .. | CESD-20>=22 | AUDIT-C>=3 (females); AUDIT-C>=4 (males) | Any drug in past 12 months |
| **Zambia** | .. | CESD-20>=22 | AUDIT-C>=3 (females); AUDIT-C>=4 (males) | Any drug in past 12 months |

Most models account for study design and for baseline values of the outcome variable to increase the precision of the estimate. The only exceptions to this are our estimates for Kenya (all-cause morbidity DALYs) only), where we report the adjusted difference at endline only, as no baseline data for depression and anxiety is available; and Ghana, where the main estimation model is a difference-in-differences model, which reports the difference in the change in the health sequela (IPV-related DALY) between the intervention and control group. With the exception of Kenya's estimates, where we do not have baseline values for the health variables, the remainder of the estimates have a similar interpretation of differences in DALYs between intervention and control group at endline, controlling for time (Pakistan), or the baseline value of the outcome variable (South Africa); or of differences in change (Ghana, Rwanda and Zambia). Table B below reports the DALY weights we used to convert each health outcome into DALYs. We computed a median disability weight that we attributed to each case of anxiety, depression or substance use.

**Table B: Disability Weights**

| **Disability Weight** | **Value** | **Source** |
| --- | --- | --- |
| **Major depressive disorder - median** | .3 | Salomon et al. (2015) |
| **Drug consumption - median** | .4 | Salomon et al. (2015) |
| **Alcohol consumption - median** | .49 | Salomon et al. (2015) |
| **Anxiety, mild** | .03 | Salomon et al. (2015) |
| **Anxiety, moderate** | .13 | Salomon et al. (2015) |
| **Anxiety, severe** | .52 | Salomon et al. (2015) |

However, the list of the health sequelae of IPV is much longer, and there is a risk that the DALYs we computed only capture a small fraction of the overall health burden of relevance to exposure to and perpetration of IPV. The diagram below shows the health sequelae of IPV. See also Campbell’s review (2002) for more details (22).


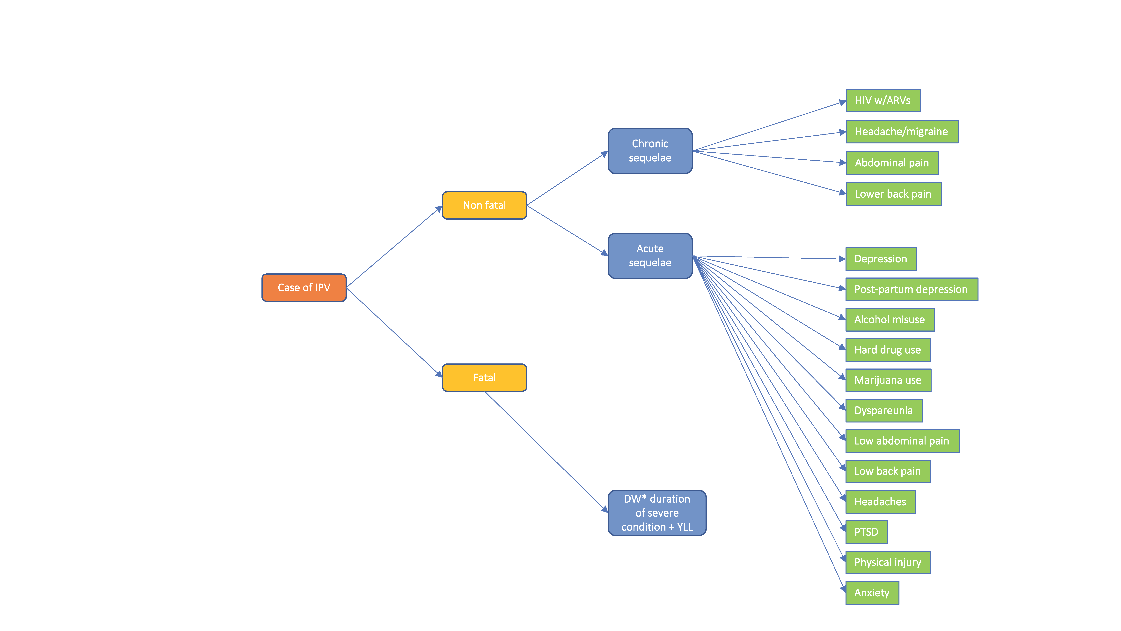


*Fig A: IPV health sequelae*

To address this limitation and to test whether the impact on IPV itself may have impacted health directly, we also compute three types of IPV-based DALYs. We call these IMAGE-benchmark DALYs, DALYs due to lifetime exposure, and DALYs due to past-year exposure. For all these calculations, we use the Institute for Health Metrics and Evaluation (IHME) IPV-based DALY computed since the Global Burden of Disease 2012 update(19). However this, too, only contains a very limited subset of the health sequelae listed above, as very few of these have been estimated in sufficiently robust studies. It is therefore likely that the IHME’s estimates of IPV-attributable DALYs are also an underestimate.

The IMAGE-benchmark DALYs use the global number of women between 15 and 49 years old and total IPV-related DALYs from the Institute of Health Metrix and Evaluation (IHME) dataset (23) with the IPV prevalence estimates for South Africa reported in Norman et al. (24) and used in Jan et al. (25) for the IMAGE study. This estimate provides a benchmark against the IMAGE calculations of Jan and co-authors. For ease of interpretation, we determine cost-effectiveness at the societal level, i.e. against the 1 x GDP per capita threshold used in the IMAGE study.

We then extract these data by age-group and country from the same dataset, and compute prevalence of lifetime and past-year IPV exposure by age-group and country using data from the most recent DHS waves: the IHME's DisMod-II model uses these datasets to estimate lifetime exposure to IPV when producing its estimates of total IPV-related DALYs we use in this analysis. We then divide the total number of DALYs by age-group and country by the number of cases of lifetime and past-year IPV exposure to obtain the DALYs per case of lifetime IPV exposure by age-group and location (IHME-lifetime), and DALYs per case of past year IPV exposure by age-group and location (IHME-past-year).
The IHME-past-year measure of health burden is likely to be closer to the amount of ill health averted in the past year by the interventions. However, in the absence of formal estimates of DALYs due to past-year IPV, we report all three of these estimates to account for the uncertainty in the DALYs attributable to past year exposure to IPV. In addition to the IMAGE-benchmark DALY, the IHME-lifetime DALY is likely to provide an upper-bound estimate, and the IHME-past-year DALY a lower bound, due to the higher likelihood of multiple episodes and cumulative health sequelae when the time period of reference is longer. However, it is also possible for the converse to be true, if the recent episodes are particularly acute.

For three separate DALY measures, we then attribute each IPV-based DALY to each case of past-year IPV exposure among study participants. We estimate the difference in IPV-related DALYs between the intervention and control group using the estimation models used to estimate each study's effect on its primary outcome. This ensures our estimates are consistent with study design and with the estimates of impact on primary outcomes reported by each study.
IPV prevalence and health burden are only available for women who are 15 years or older, so these sensitivity analyses are on adult women (15+) only. All children are dropped, and Kenya and Pakistan not included in this analysis. We also use the IHME data on women to proxy for IPV's health impact on men and produce our total (females and males) estimates for each study. This approach has limitations, as the health sequelae from IPV perpetration may in part differ from exposure-related sequelae. However, DALYs from IPV perpetration have not been computed, to our knowledge. Finally, we omit estimates of the health burden from peer-to-peer violence among children from this analysis, because the IHME repository only has data on bullying victimisation, rather than peer-to-peer violence

## Probabilistic sensitivity analysis - distributional assumptions and parameters

To estimate cost-effectiveness consistently with willingness to pay thresholds in the literature, we computed the cost per DALY averted by each intervention. This can then be compared with the cost-per DALY that either corresponds to policy makers’ revealed opportunity cost (26), or WHO’s GDP per capita threshold. Given the uncertainty in our estimates, we did a probabilistic sensitivity analysis (PSA) and reported the likelihood of cost-effectiveness for each intervention, given the policy maker's willingness to pay. The PSA rests on the following distributional assumptions and parametrization for costs and effects. DALYs and net costs, which are differences between groups and can assume negative values, follow Gaussian distributions with mean (µ) and variance (σ^2^) as found in trial-based estimates. For provider costs, we assume the data is generated by a Gamma distribution whose shape (α) and rate (β) parameters are such that their product is equal to the mean cost found in the cost analysis.

We first generate 10,000 independent random draws from each distribution and test that the resulting realisations are distributed as expected. We then use non-parametric bootstrapping to draw 10,000 cost and DALY pairs with replacement to populate the cost-effectiveness plane. The probability that the intervention is cost-effective is given by the percentage of cost-effect pairs found south and east of the ray through the origin with slope equal to the policy maker's willingness to pay in each country. Tables C and D below report distributional assumptions and parameter values used in the probabilistic sensitivity analysis (PSA).


**Table C: Females only - distributional assumptions and parameters for probabilistic sensitivity analysis**

|  | **DALYs** | | **Costs** | | | |
| --- | --- | --- | --- | --- | --- | --- |
|  |  |  | **Net** | | **Provider** | |
| **Distribution** | **Normal** | | **Normal** | | **Gamma** | |
|  | **µ** | **σ** | **µ** | **σ** | **α** | **β** |
| **Kenya** | 0.010 | 0.020 | .. | .. | 8.620 | 1.310 |
| **Pakistan** | 0.000 | 0.020 |  |  | 10.550 | 2.110 |
| **Ghana** | 0.080 | 0.050 | 87 | 223.29 | 3.930 | 1.000 |
| **Rwanda** | 0.010 | 0.010 | 21 | .02 | 12.770 | 1.590 |
| **South Africa** | 0.000 | 0.030 | -295 | 219.51 | 79.890 | 4.000 |
| **Zambia** | 0.170 | 0.060 | .. | .. | 81.440 | 16.290 |

**Table D: Females and males - distributional assumptions and parameters for probabilistic sensitivity analysis**

|  | **DALYs** | | **Costs** | | | |
| --- | --- | --- | --- | --- | --- | --- |
|  |  |  | **Net** | | **Provider** | |
| **Distribution** | **Normal** | | **Normal** | | **Gamma** | |
|  | **µ** | **σ** | **µ** | **σ** | **α** | **β** |
| **Kenya** | .. | .. | .. | .. | .. | .. |
| **Pakistan** | 0.010 | 0.020 | .. | .. | 10.550 | 2.110 |
| **Ghana** | 0.010 | 0.100 | -356 | 372.58 | 3.930 | 1.000 |
| **Rwanda** | 0.000 | 0.000 | 21 | .02 | 12.770 | 1.590 |
| **South Africa** | 0.020 | 0.020 | 1809 | 1799.53 | 79.890 | 4.000 |
| **Zambia** | 0.140 | 0.060 | .. | .. | 81.440 | 16.290 |

# Additional analyses

We report two additional sets of analyses.

The first is a set of sensitivity analyses reporting interventions' probability of being cost-effective in their respective research setting, using IPV-based DALYs computed on IHME and DHS data (see Figs B-D). Costs for these scenarios are given in Table 2, and more details can be found in Torres-Rueda et al. (1). We assume that the effects found in the research setting are sustained at scale.
The second is a set of sub-group analyses reporting the cost-effectiveness of the Indashyikirwa VSLA+ only intervention, focusing on a subset of beneficiaries in Rwanda in a research setting and in two scale-up scenarios. This analysis is important, because this group is distinct from the general population in that (i) it is a group of VSLA clients, who may differ from the general population in observable and non-observable ways and (ii) it is a group in receipt of a different intervention compared to the village communities in which it resides. The men and women included in this analysis received a gender transformative training in small workshop settings. Following this initial phase some of them also received some training in community mobilisation. This intensity of treatment sets them apart from other Indashyikirwa recipients and merits a separate investigation. We report this analysis in Figs E-F and tables B-C.

## Sensitivity analyses: IHME DALY from IPV

This section reports results of the probabilistic sensitivity analysis of interventions' cost-effectiveness from a societal perspective in a research setting using DALYs based on IHME and DHS data. Fig B below reports estimates for the IMAGE-benchmark DALY scenario. These results present similar patterns to the cost-effectiveness analysis based on all-cause morbidity for South Africa and Ghana. The Indashyikirwa intervention for VSLA participants is less likely to be cost-effective according to the IMAGE-benchmark DALY than the all-cause morbidity DALY (see below).


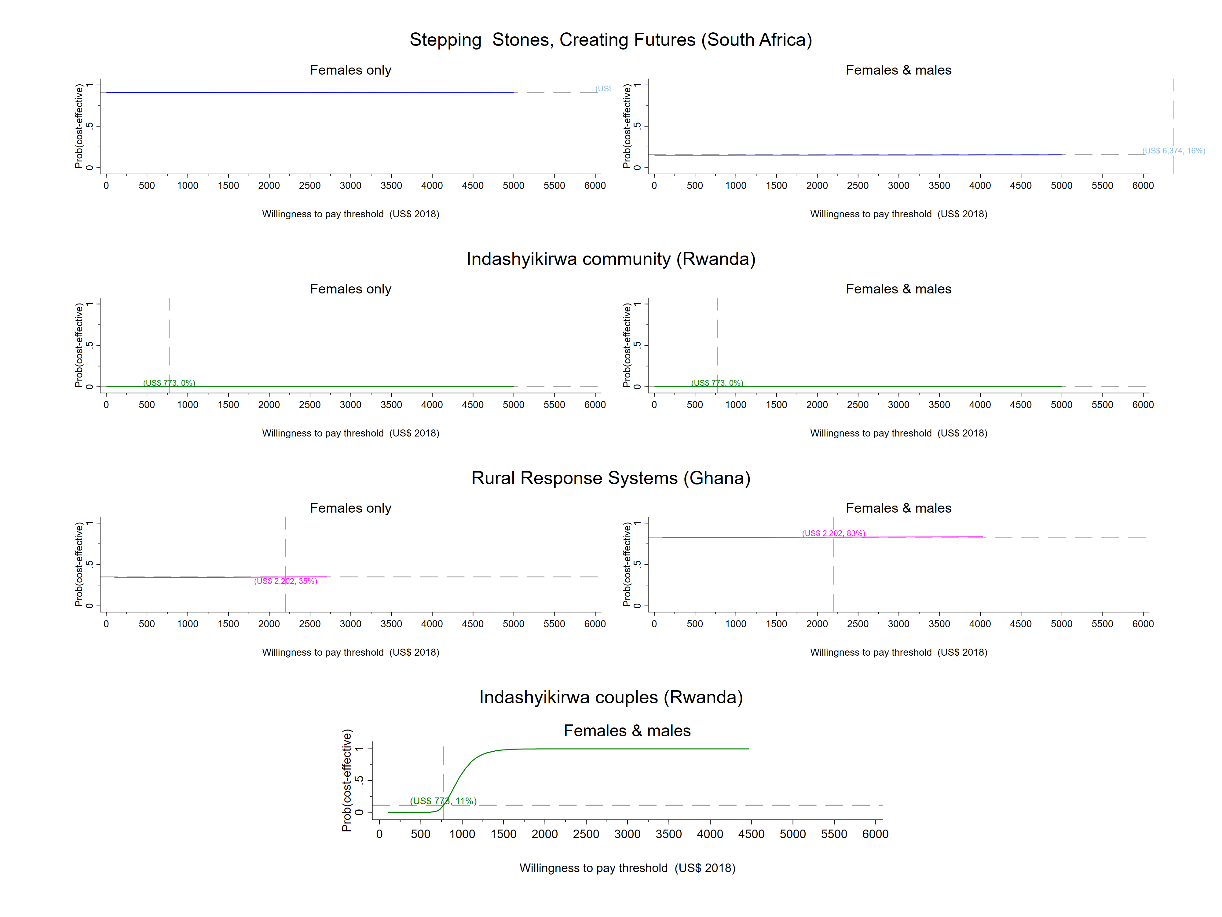


*Fig B: Cost-effectiveness Acceptability Curves based on the IMAGE-benchmark DALY, research setting, societal perspective. Dashed vertical line: country-specific opportunity cost threshold; dashed horizontal line: probability that the intervention is cost-effective at the country-specific threshold, given the cost per DALY averted by the intervention.
IPV DALY data from GBD Results tool. Source: Global Burden of Disease Collaborative Network. Global Burden of Disease Study 2017 (GBD 2017) Results. Seattle, United States: Institute for Health Metrics and Evaluation (IHME), 2018. Available from http://ghdx.healthdata.org/gbd-results-tool.*

Probabilities of cost-effectiveness are as follows: South Africa, females: 91%; South Africa, females and males: 16%. Rwanda community, females: 0%; Rwanda community, females and males: 0%. Ghana, females: 35%; Ghana, females and males: 83%. Rwanda, couples only: 11%.

Results are not sensitive to IHME DALY specification, as Figs C and D illustrate.


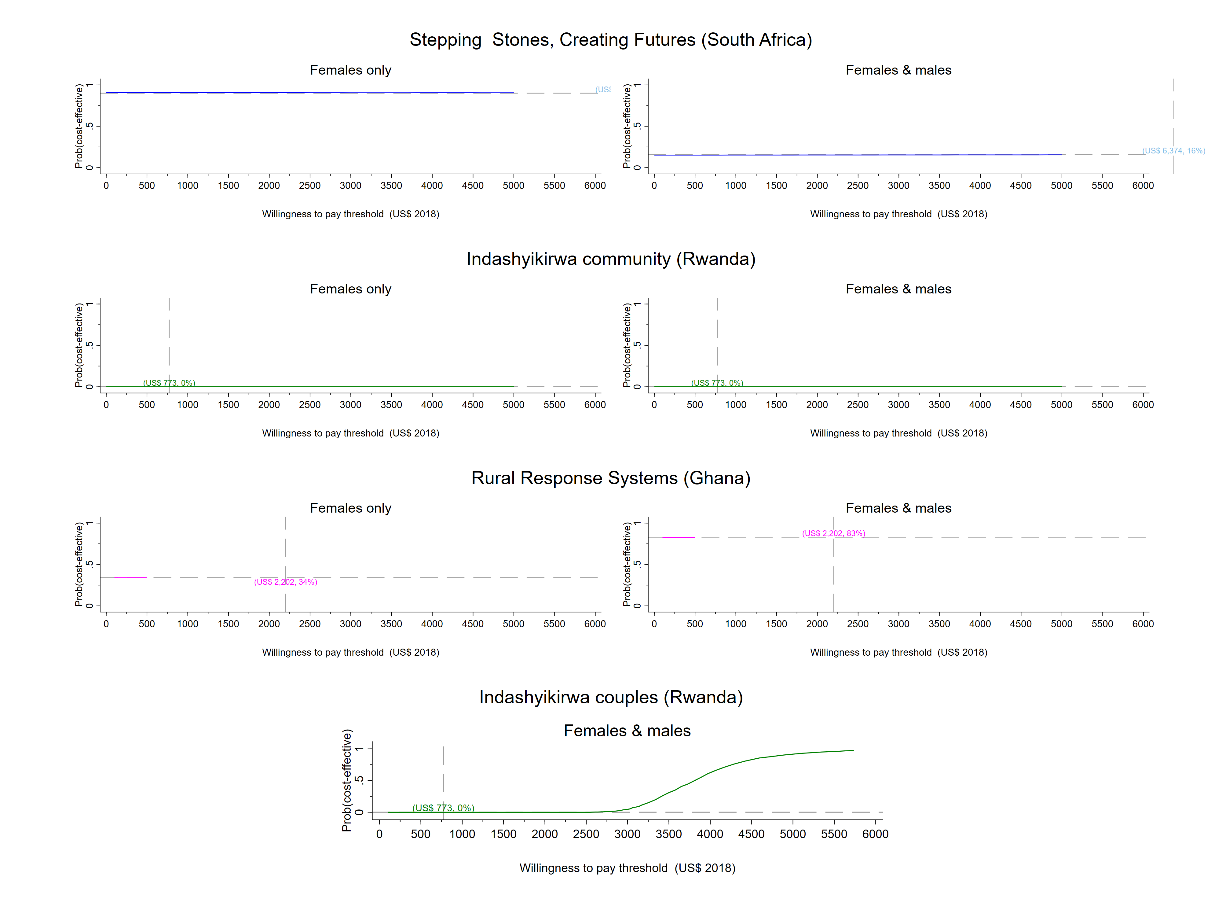


*Fig C: Cost-effectiveness Acceptability Curves based on the IHME-DALY for ever-exposure to IPV in respondent's age bracket and country, research setting, societal perspective. Dashed vertical line: country-specific opportunity cost threshold; dashed horizontal line: probability that the intervention is cost-effective at the country-specific threshold, given the cost per DALY averted by the intervention.
IPV DALY data from GBD Results tool. Source: Global Burden of Disease Collaborative Network. Global Burden of Disease Study 2017 (GBD 2017) Results. Seattle, United States: Institute for Health Metrics and Evaluation (IHME), 2018. Available from http://ghdx.healthdata.org/gbd-results-tool. IPV prevalence from DHS datasets.*

Probabilities of cost-effectiveness are as follows: South Africa, females: 90%; South Africa, females and males: 16%. Rwanda community, females: 0%; Rwanda community, females and males: 0%. Ghana, females: 34%; Ghana, females and males: 83%. Rwanda, couples only: 0%.


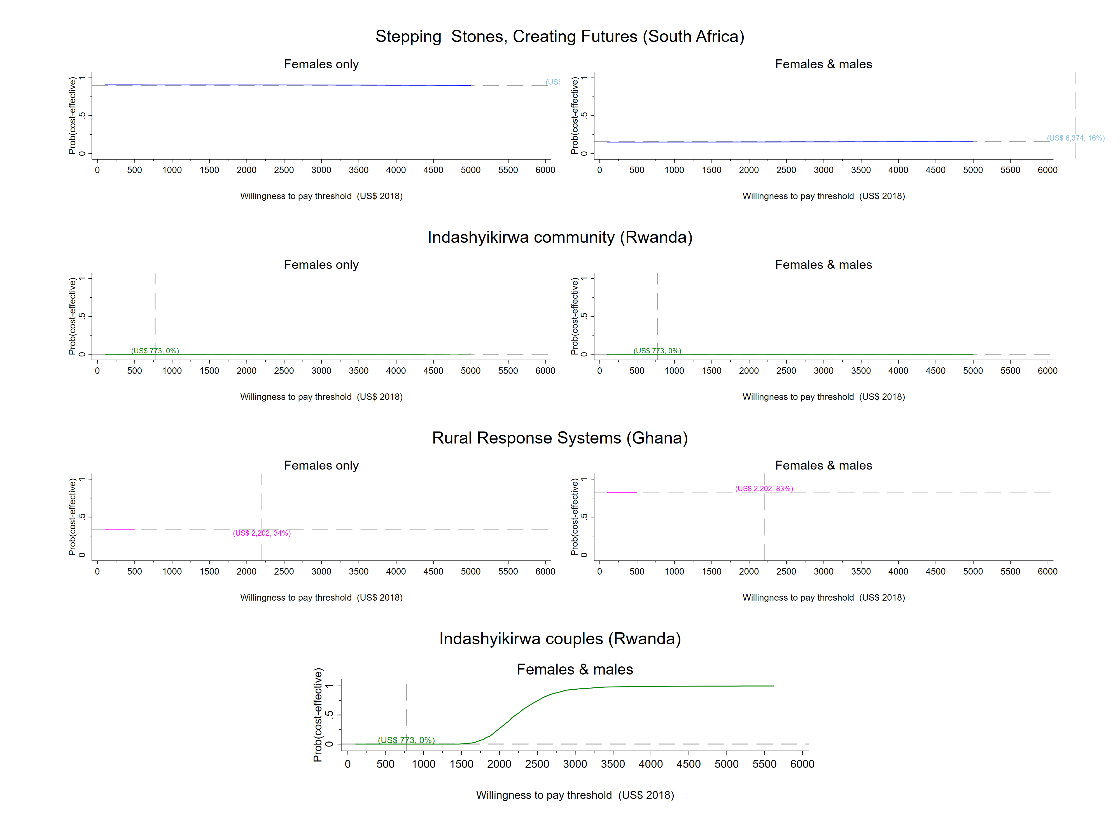


*Fig D: Cost-effectiveness Acceptability Curves based on the quota of DALYs from past-year exposure to IPV in respondent's age bracket and country that can be attributed to past-year exposure, research setting, societal perspective. Dashed vertical line: country-specific opportunity cost threshold; dashed horizontal line: probability that the intervention is cost-effective at the country-specific threshold, given the cost per DALY averted by the intervention.
IPV DALY data from GBD Results tool. Source: Global Burden of Disease Collaborative Network. Global Burden of Disease Study 2017 (GBD 2017) Results. Seattle, United States: Institute for Health Metrics and Evaluation (IHME), 2018. Available from http://ghdx.healthdata.org/gbd-results-tool. IPV prevalence from DHS datasets.*

Probabilities of cost-effectiveness are as follows: South Africa, females: 90%; South Africa, females and males: 16%. Rwanda community, females: 0%; Rwanda community, females and males: 0%. Ghana, females: 34%; Ghana, females and males: 83%. Rwanda, couples only: 0%.

Overall, these results are consistent with the patterns observed in the main analysis. Interventions with larger impact on IPV exposure show higher likelihood of cost-effectiveness than others, if they are comparatively cheaper. However, interventions with positive economic impact can be cost-saving and have a high likelihood of achieving cost-effectiveness in the short-run, even in the face of modest reductions in IPV risk. For example, the IMAGE intervention is more cost-effective than the SSCF intervention from a provider perspective in South Africa, when both are assessed against the IMAGE-benchmark DALY. However, the SSCF intervention is likely to be cost-effective from a societal perspective for both men and women, when its economic impacts are also taken in to account and as the threshold expands.
In future research, it will be important to investigate whether an economic improvement in the short run may lead to reduced IPV exposure in the medium to long term and, conversely, if a reduction in exposure to IPV may in turn improve recipients' economic outcomes in the medium to long term.
It is important to note that, although patterns are similar, the likelihood of cost-effectiveness using IPV-related DALYs is smaller than the likelihood of cost-effectiveness for all-cause morbidity in our main analysis. These patterns support the hypothesis that the interventions impact health in various ways, and not only via a reduction in IPV exposure or perpetration, as also suggested by the IMpower results overall.

## Sub-group analyses: Rwanda Couples’ intervention in research and scale-up settings

In Rwanda, couples in which one of the spouses was a Village Savings and Loans Associations (VSLAs) member, were offered the opportunity to be trained in the Indashyikirwa curriculum if their VSLA was located in a sector randomised to the treatment arm. Eligible volunteers in the intervention and control arms were randomly selected to become study participants through a public random lottery. The Indashyikirwa couples' curriculum focuses on raising awareness of positive and negative forms of power, personal reflection and aspirations for relationships and on non-violent skills for the solution of disagreements.
Sectors were the primary sampling unit because the full Indashyikirwa programme targeted communities (27), with some of the VSLA couples eventually training as community mobilisers. Sampling was stratified by districts. The analysis is an intention to treat (ITT) at the individual level. It reports differences in means (continuous outcomes) or odds ratios (binary outcomes) between intervention and control arm at 24 months, accounting for baseline imbalances and time. It uses generalized linear mixed models that account for the complex sampling structure with district-level fixed effects (strata) and sector and couple random effects to acknowledge the nested nature of the sample. The model uses a logit link for binary outcomes, and a gaussian link for continuous outcomes. For the economic evaluation, DALYs are considered continuous, and we use a Gaussian link function.
The Indashyikirwa gender-transformative training offered to VSLAs members in Rwanda reduced exposure and perpetration of IPV among the treated, compared to controls, and is 50% likely to be cost-effective from a provider perspective. The likelihood of cost-effectiveness increases to more than 70% once the intervention is scaled up to the population of potential VSLA clients.


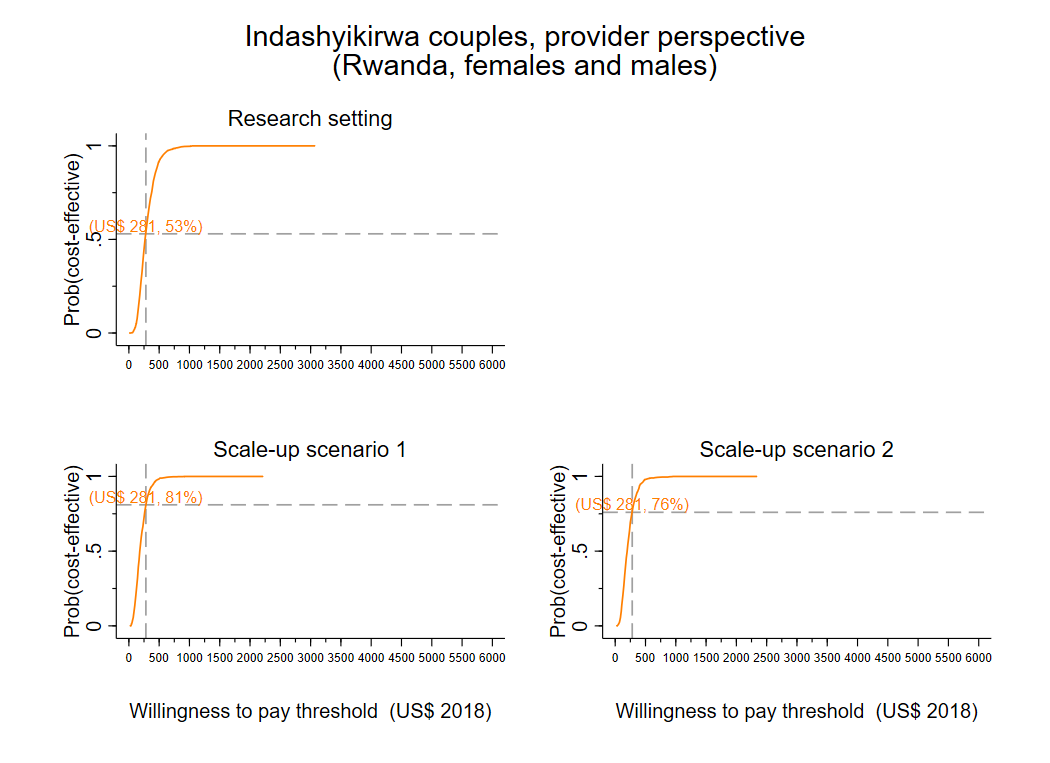


*Fig E: Cost-effectiveness Acceptability Curves for the Indashyikirwa VSLA+ intervention delivered to couples in Rwanda, provider perspective. Dashed vertical line: country-specific opportunity cost threshold; dashed horizontal line: probability that the intervention is cost-effective at the country-specific threshold, given the cost per DALY averted by the intervention.*

From a societal perspective the likelihood of cost effectiveness increases to 100% even in the face of no apparent economic impact. The reason for the increase in the probability of cost-effectiveness is that the GDP per capita threshold is almost treble the opportunity cost threshold, relaxing the budget constraint.


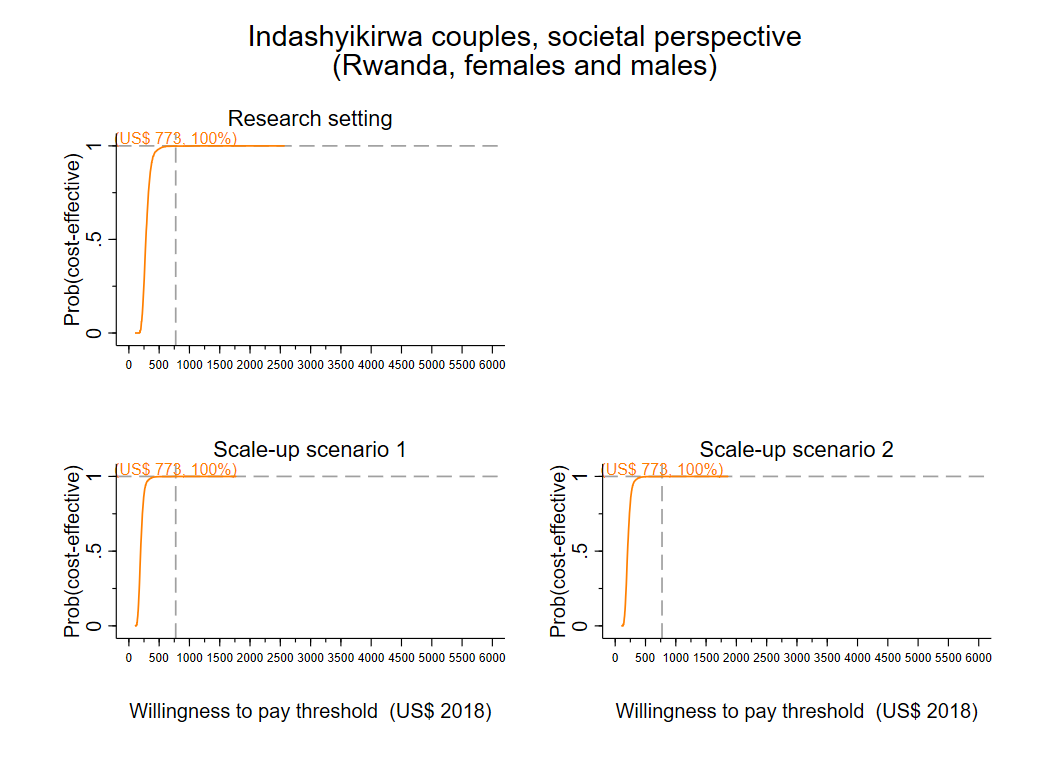


*Fig F: Cost-effectiveness Acceptability Curves for the Indashyikirwa VSLA+ intervention delivered to couples in Rwanda, societal perspective. Dashed vertical line: country-specific opportunity cost threshold; dashed horizontal line: probability that the intervention is cost-effective at the country-specific threshold, given the cost per DALY averted by the intervention.*

**Table E: Outcomes at 24 months by intervention arm (24 months post baseline)**

|  | **All** | | |
| --- | --- | --- | --- |
|  | **Intimate partner violence** | **Depression** | **Hazardous alcohol use** |
| **Intervention percentage (N=1618)** | 0.33 | 0.13 | 0.04 |
| **Control percentage (N=1613)** | 0.38 | 0.18 | 0.04 |
| **adjusted Odds Ratio (N=3231)** | 0.57 | 0.53 | 1.09 |
| **95% CI** | (0.47 to 0.70) | (0.41 to 0.68) | (0.67 to 1.77) |

**Table F: Cost-effectiveness females (IPV experience) and males (IPV perpetration)**

|  | **Females (IPV experience) and males (IPV perpetration)*** | |
| --- | --- | --- |
|  | **Indashyikirwa VSLA+ for couples** | |
|  | **Rwanda** | |
|  | **Estimate** | **95% CI** |
| **Provider perspective** | | |
| 1 IPV-free years per capita | .55 | (.36 to .74) |
| 2 IPV-free person-years gained (1 x N in table E) | 889.9 | (582.48 to 1197.32) |
| 3 DALYs averted per participant | .02 | (.01 to .03) |
| 4 DALYs averted during the study period (3 x N in table E) | 32.36 | (16.18 to 48.54) |
| 5 DALYs averted per thousand participants (3 x 1,000) | 20 | (10 to 30) |
| 6 Provider cost per capita | $ 6.34 |  |
| 7 Cost per year free from IPV gained (6/1) | $ 11.42 |  |
| **8 Cost per DALY averted (6/3)** | **$ 280.19** |  |
| **Opportunity cost threshold** | **$ 281** |  |
| **Societal perspective** | | |
| 9 Economic impact per capita | $ .002 | (.001 to .003) |
| 10 Net cost (9+6) | $ 6.34 |  |
| **11 Cost per DALY averted (10/3)** | **$ 280.09** |  |
| **GDP per capita threshold** | **$ 773** |  |
| *Intention to treat estimates: values are calculated with reference to all females enrolled at baseline | | |

# Cost-effectiveness planes

Fig G below show all 10,000 bootstrapped cost and effect pairs for each intervention from the provider perspective. These graphs highlight, for example, that a high dispersion of the effect estimates on both sides of the origin coupled with relatively high costs seem to explain the low likelihood of cost-effectiveness for South Africa and Rwanda (community). The sharp increase in the likelihood of cost-effectiveness in Pakistan's CEAC is explained by the high density of effectiveness points at high values of the x-axis. However, an opportunity cost threshold at US$ 177 and a provider cost of US$ 22 per female student imply that even the relatively small uncertainty in effect, as signalled by the cluster of points around the origin, yields a 0% likelihood of cost-effectiveness in Pakistan. In contrast, the IMpower intervention in Kenya achieves a 30% likelihood of cost-effectiveness despite higher uncertainty in effects, at US$ 11 per student and an opportunity cost threshold of US$ 656. Finally, the two interventions in Rwanda show substantial differences in costs, with the couples' intervention costing one third of the community-level intervention per capita. The couples' intervention is also substantially more effective, even if it still records some uncertainty in the estimate. This shifts the bootstrap cloud much further away from the origin in the first quadrant and yields a probability of cost effectiveness of 53%.


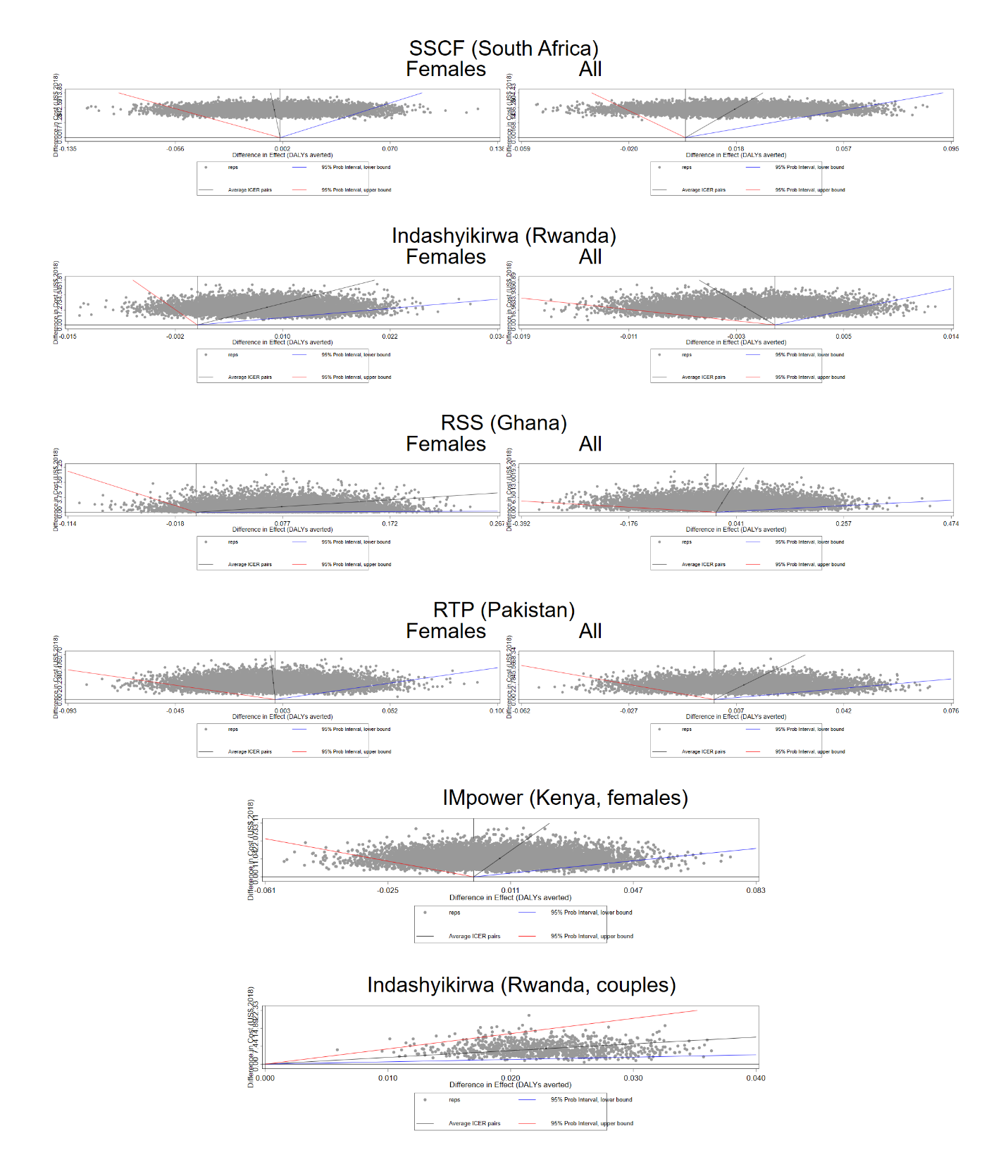


*Fig G: Cost-effectiveness planes, all interventions, provider perspective*


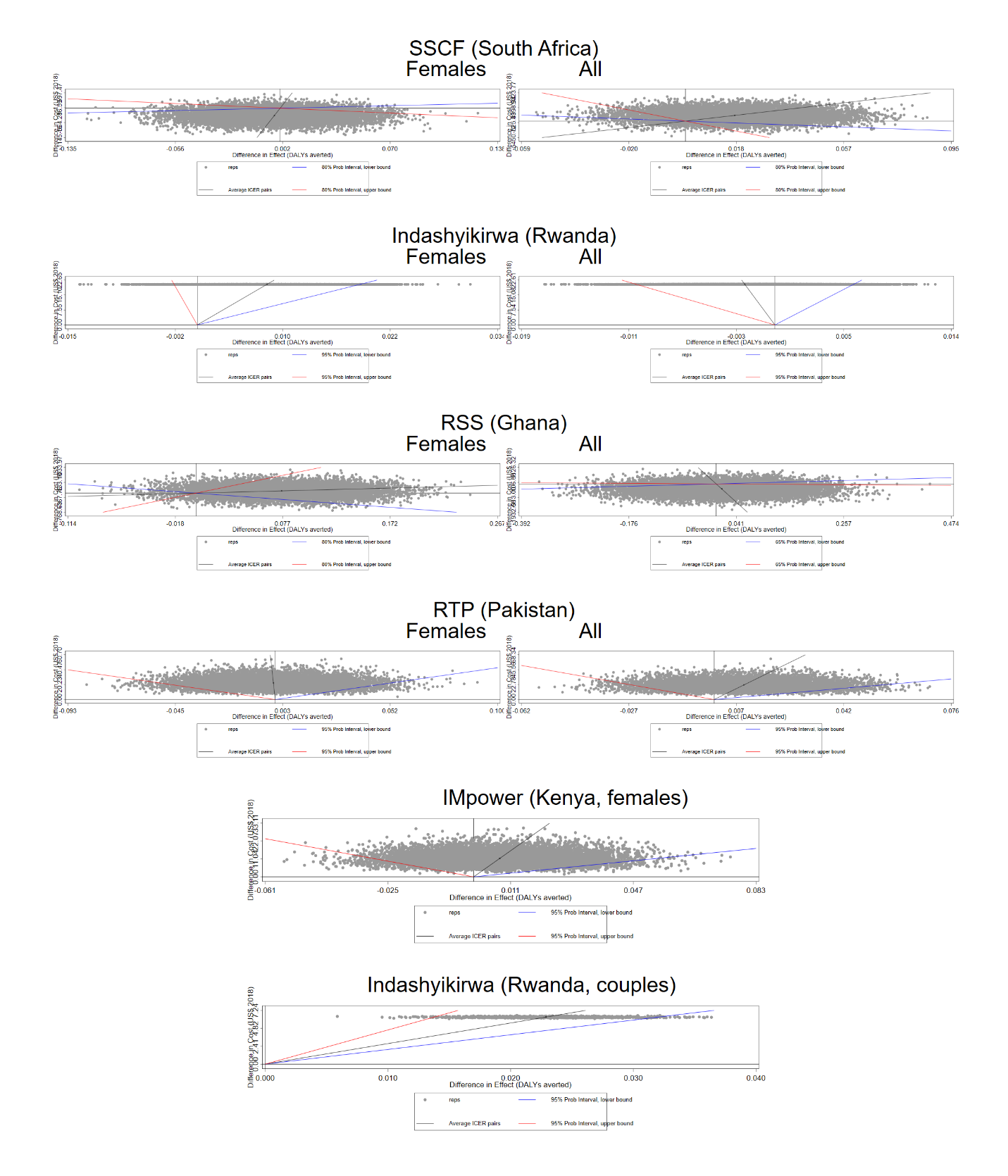


*Fig H: Cost-effectiveness planes, all interventions, societal perspective*

# Cost per year free from violence

The table below reports cost per year free from intimate partner violence or peer-to-peer violence, for comparison with other IPV or PPV interventions. Among the community level interventions, RSS's US$ 123 (2018 prices) per case of past-year IPV averted among female participants in Ghana compares favourably to SASA!'s US$ 460 (2011 prices) reported by Michaels-Igbokwe et al (28) and to UBL’s US$ 194 (2015 prices) (29). The community-level intervention in Rwanda records a negative point estimate, though this is not statistically different from zero, and cost per past-year cases of IPV averted is not reported. Right To Play in Pakistan compares favourably with the Good School Toolkit in Uganda, at US$ 56 per case of corporal punishment averted (2018 prices), versus Uganda's US$ 96 (2015 prices) (30)

**Table G: Cost per IPV- or corporal punishment-free year**

|  |  | **Provider perspective** | | **Societal perspective** | |
| --- | --- | --- | --- | --- | --- |
|  |  | **Costs (US$ 2018)** | | **Costs (US$ 2018)** | |
| **Location** | **IPV- or corporal punishment-free person-years per capita** | **Cost per capita** | **Cost per IPV- or corporal punishment-free year gained** | **Cost per capita** | **Cost per IPV- or corporal punishment-free year gained** |
| **Females only** | | | | | |
| **Ghana** | 0.032 | $ 3.9 | $ 123 | $ 87 | $ 2,722 |
| **Kenya &** | -0.009 | $ 11 | .. | $ 11 | .. |
| **Pakistan corporal punishment §** | 0.75 | $ 22 | $ 30 | $ 22 | $ 30 |
| **Rwanda community** | -0.214 | $ 20 | .. | $ 21 | .. |
| **South Africa** | 0.017 | $ 319 | $ 18,789 | $ -295 | cost-saving and effective |
| **Zambia^** | 0.165 | $ 1,324 | $ 8,022 | ** | ** |
| **Females and males*** | | | | | |
| **Ghana** | 0.033 | $ 3.9 | $ 120 | $ -356 | cost-saving and effective |
| **Kenya &** | .. | .. | .. | .. | .. |
| **Pakistan corporal punishment §** | 0.40 | $ 22 | $ 56 | $ 22 | $ 56 |
| **Rwanda community** | -0.150 | $ 20 | .. | $ 20 | .. |
| **South Africa** | 0.046 | $ 319 | $ 6,944 | $ 1,809 | $ 39,324 |
| **Zambia^** | 1.074 | $ 1,324 | $ 1,232 | ** | ** |
| § This is an alternative unit cost, for comparison with the Good School toolkit in Uganda, which cost US$96 per case of corporal punishment averted during the trial at 2015 prices ^ We collected the economic data for this analysis and at the time of writing we are waiting for the data to be shared with us.  * IPV-free years here are the sum of IPV exposure for females and IPV perpetration for males & Kenya reports only impact on physical IPV on females | | | | | |

# Impact models – goodness of fit considerations

We defined the models based on each intervention’s theory of change and conceptual model as defined by implementers and impact evaluation teams. This choice enables us to test interventions’ impact on VAWG and health reporting comparable estimates to those in trial the papers. Further details on model selection can be found there.

For completeness, Table H also reports goodness of fit tests for the impact models reported in Tables 2 and E. We report the Wald statistic to assess model fitness in all generalised linear models. The Wald statistic has a limiting Chi-squared distribution, and the joint hypothesis of no effect can be rejected at the 99.9% level if the probability of finding a value of the Wald statistic greater than the one reported is less than 0.001 under the joint null hypothesis. Where available, we also report the log-likelihood that models are maximising. Estimates for the Rural Response Systems intervention in Ghana are computed using a difference-in-differences estimator (Table 1), and we report the models’ R-squared, to capture the fraction of the outcomes’ sample variation explained by the model.

**Table H: Impact models goodness of fit**

|  |  |  | **Women and girls only** | | | | | **All (exposure and perpetration)§** | | | | |
| --- | --- | --- | --- | --- | --- | --- | --- | --- | --- | --- | --- | --- |
|  | **Model type** | **Goodness of fit statistics** | **Intimate partner violence** | **Peer to peer victimisation** | **Depression** | **Hazardous alcohol use** | **Anxiety** | **Intimate partner violence** | **Peer to peer victimisation** | **Depression** | **Hazardous alcohol use** | **Anxiety** |
| **RRS‡** | Difference-in-differences | R-squared | 0.20 | .. | 0.34 | 0.02 | .. | 0.04 | .. | 0.22 | 0.33 | .. |
| **IMpower** | Generalized linear mixed model for change | Log pseudolikelihood | -1083.18 |  | -1248.10 |  | -993.26 |  |  |  |  |  |
|  |  | Wald test of joint significance | 53.15 | .. | 2.72 | .. | 5.08 | .. | .. | .. | .. | .. |
|  |  | Prob > chi2 | 0.0000 | .. | 0.4375 | .. | 0.1663 | .. | .. | .. | .. | .. |
| **RTP** | Generalized linear mixed model for change | Log likelihood |  | -1328.88 | -682.94 |  |  |  | -1957.22 | -1432.34 |  |  |
|  |  | Wald test of joint significance | .. | 120.91 | 51.74 | .. | .. | .. | 132.33 | 63.70 | .. | .. |
|  |  | Prob > chi2 | .. | 0.0000 | 0.0000 | .. | .. | .. | 0.0000 | 0.0000 | .. | .. |
| **Indashyikirwa**  **Community** | Generalized linear mixed model for change | Log likelihood | -1764.29 |  |  | -533.63 |  | -3702.81 |  |  | -1604.98 |  |
|  |  | Wald test of joint significance | 125.60 | .. | .. | 37.65 | .. | 187.50 | .. | .. | 111.03 | .. |
|  |  | Prob > chi2 | 0.0000 | .. | .. | 0.0003 | .. | 0.0000 | .. | .. | 0.0000 | .. |
| **Indashyikirwa**  **Couples** | Generalized linear mixed model for change | Log likelihood |  |  |  |  |  | -4616.00 |  | -3019.87 | -915.37 |  |
|  |  | Wald test of joint significance | .. | .. | .. | .. | .. | 1998.25 |  | 1356.23 | 284.97 |  |
|  |  | Prob > chi2 | .. | .. | .. | .. | .. | 0.0000 |  | 0.0000 | 0.0000 |  |
| **SSCF** | Generalized linear model first difference | Wald test of joint significance | 31.49 | .. | 16.53 | 16.41 | .. | 31.49 | .. | 16.53 | 16.41 | .. |
|  |  | Prob > chi2 | 0.0000 | .. | 0.0003 | 0.0003 | .. | 0.0000 | .. | 0.0003 | 0.0003 | .. |
| **VATU*** | Generalized linear mixed model for change | Log pseudolikelihood | -344.06 |  | -368.89 | -361.01 |  | -675.73 |  | -736.82 | -745.96 |  |
|  |  | Wald test of joint significance | 1152.11 | .. | 992.57 | 345.45 | .. | 738.86 | .. | 447.91 | 3307.33 | .. |
|  |  | Prob > chi2 | 0.0000 | .. | 0.0000 | 0.0000 | .. | 0.0000 | .. | 0.0000 | 0.0000 | .. |
| RSS: Rural Response Systems; SSCF: Stepping Stones Creating Futures; RTP: Right To Play; VATU: Violence and Alcohol Treatment | | | | | | | | | | | | |

Wald tests reject the joint null hypothesis, that is that the independent variables do not jointly explain the outcome. The only exception is the Kenya intervention for anxiety and depression. These models are cross-sectional, and relied on fewer observations than the VAWG model for the IMpower intervention, which may explain the failure to reject that the model as a whole is non-explanatory. Investigating models’ predictions shows that the VAWG model correctly predicts 64% of the original VAWG responses, the depression model 86%, and the anxiety model 27%. The R-squared drops for the alcohol model in Ghana as fewer observations were available, compared to the VAWG and depression models. In both cases, the lack of fit may be determined by the small number of observations.

Moreover, when using the point estimates from the regression in Table 2 for the cost-effectiveness analysis, we characterise the uncertainty in these parameters with probabilistic sensitivity analysis. We first assign distributions to all estimates based on theory and available evidence on each parameter’s moment generating function. We then draw random samples from these distributions using bootstrapping techniques to derive the cost-effectiveness estimates presented in Table 4 with the respective probability of cost-effectiveness (see Methods section in the manuscript for more details, and Table 4 for results). This additional characterisation of uncertainty mitigates any potential sub-optimal fit and lack of precision in the estimates from the models applied to the primary data.

Testing how well the models fit the data is useful. However, the key criterion for model selection is the interventions’ theory of change, i.e. the conceptual mechanism of effect that links interventions to outcomes which the RCTs and our models were designed to test.

# Impact inventories

In line with the recommendations from the second panel on cost-effectiveness, we also report impact inventories for each intervention. (see Figs F-I). The impact inventories show that a large portion of the costs and benefits associated with VAWG prevention interventions fall in non-health sectors, such as labour market, social protection, education and legal or criminal justice. Some of these were measured in the What Works studies (see black boxes in the impact inventories, but are hard to attribute a value to, such as changes in gender attitudes. Other non-health domains were not measured (see legal or criminal justice costs). In addition, a number of health domains were either only partially measured or not measured at all. Future efforts in this field should aim to collect more detailed data on both health and non-health costs and benefits associated with consequences and responses to VAWG, in addition to the short-terms costs and benefits from prevention, to arrive at more comprehensive estimates of cost-effectiveness.


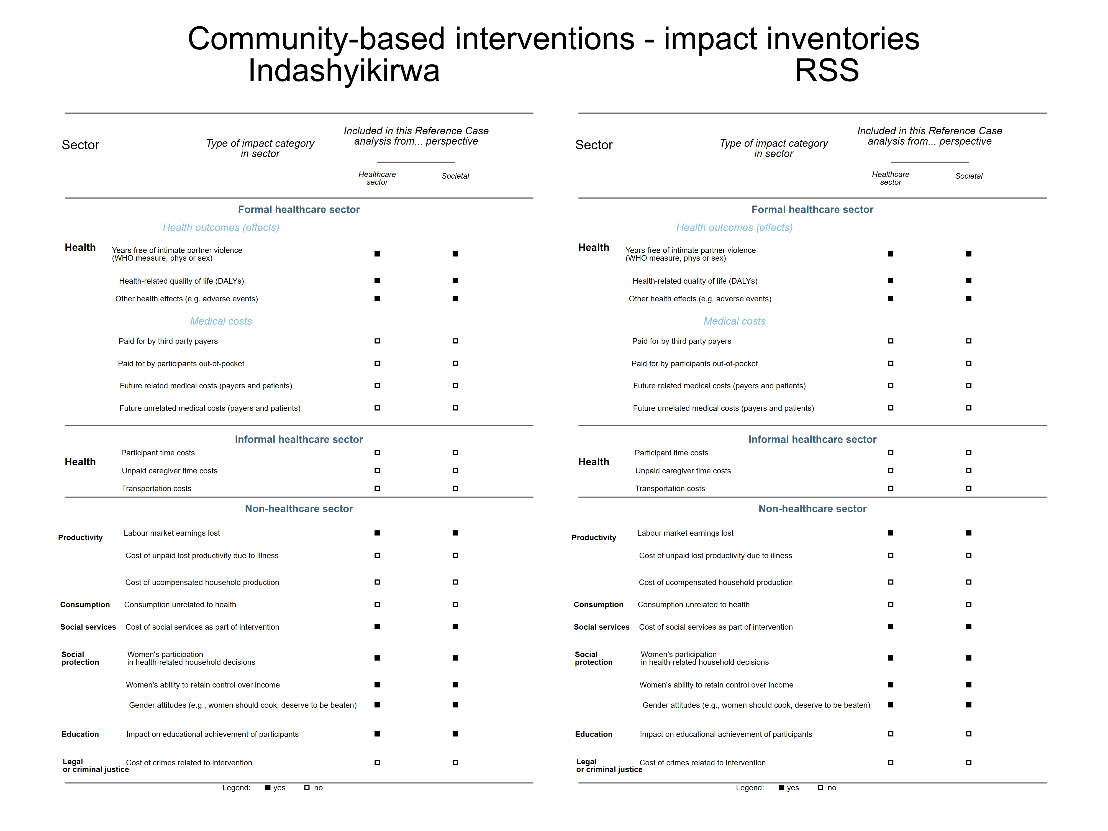


*Fig J: Impact inventories of community-based interventions*


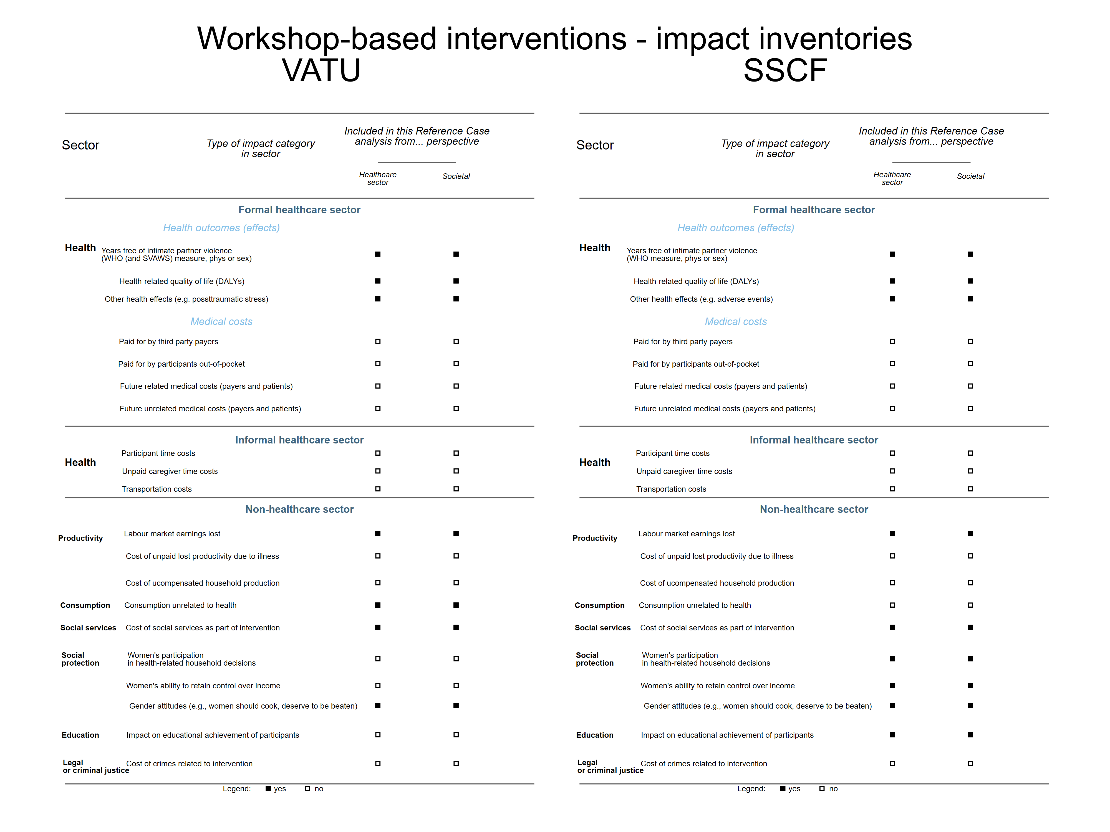


*Fig K: Impact inventories of workshop-based and one-to-one interventions*


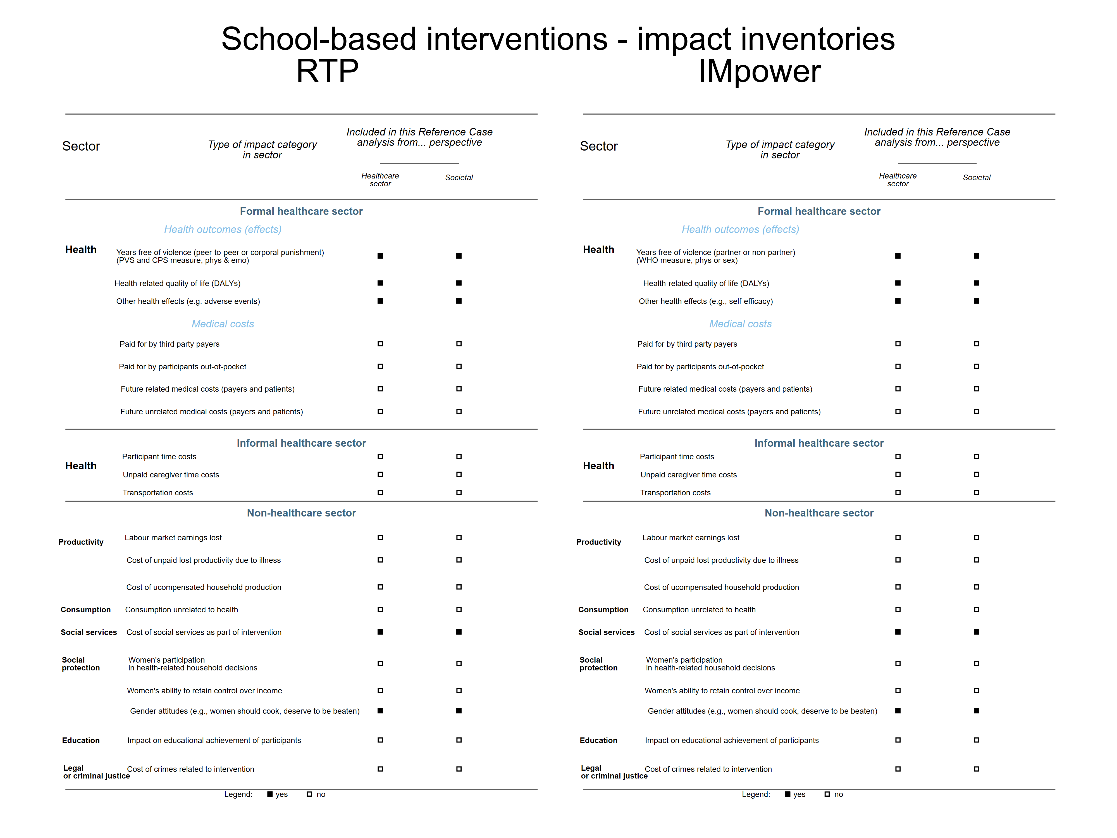


*Fig L: Impact inventories of classroom-based interventions in schools*

# References

1. Torres-Rueda S, Ferrari G, Orangi S, Hitimana R, Daviaud E, Tawiah T, et al. What will it cost to prevent violence against women and girls in low- and middle-income countries? Evidence from Ghana, Kenya, Pakistan, Rwanda, South Africa and Zambia. Health Policy Plan. 2020 Jun 18;35(7):855–66.

2. Ferrari G, Torres-Rueda S, Michaels-Igbokwe C, Watts C, Jewkes R, Vassall A. Economic Evaluation of Public Health Interventions: An Application to Interventions for the Prevention of Violence Against Women and Girls Implemented by the “What Works to Prevent Violence Against Women and Girls?” Global Program. J Interpers Violence. 2019;

3. Ferrari G, Torres-Rueda S, Michaels-Igbokwe C, Watts C, Vassall A. Guidelines for Conducting Cost Analyses of Interventions Aimed at Preventing Violence against Women and Girls in Low- and Middle-Income Settings [Internet]. London, UK; 2018 [cited 2021 Apr 19]. Available from: https://www.whatworks.co.za/resources/project-resources/item/557-guidelines-for-conducting-cost-analyses-of-interventions-to-prevent-violence-against-women-and-girls-in-low-and-middle-income-settings

4. Vassall A, Sweeney S, Kahn JG, Gomez G, Bollinger L, Marseille E, et al. Reference Case for Estimating the Costs of Global Health Services and Interventions. London, UK; 2017.

5. Husereau D, Drummond M, Petrou S, Carswell C, Moher D, Greenberg D, et al. Consolidated health economic evaluation reporting standards (CHEERS)-explanation and elaboration: A report of the ISPOR health economic evaluation publication guidelines good reporting practices task force. Value Heal. 2013 Mar;16(2):231–50.

6. DfID. DFID’s Approach to Value for Money (VfM) Department for International Development [Internet]. London, UK; 2011 [cited 2021 Oct 27]. Available from: http://www.dfid.gov.uk/Documents/publications1/mar/multilateral_aid_review.pdf

7. Kasteng F, Settumba S, Källander K, Vassall A. Valuing the work of unpaid community health workers and exploring the incentives to volunteering in rural Africa. Health Policy Plan [Internet]. 2016 Mar 1 [cited 2021 Oct 27];31(2):205–16. Available from: https://pubmed.ncbi.nlm.nih.gov/26001813/

8. OANDA. Currency Converter. 2018.

9. World Health Organization. Scaling up health services: Challenges and choices. Geneva; 2008.

10. National Institute of Statistics of Rwanda. Rwanda Population and Housing Census. 2012.

11. Ghana Statistical Service. National Census. 2012;

12. Kenya National Bureau of Statistics. Economic Survey. 2018.

13. Ministry of Federal Education and Professional Training. Pakistan Education Statistics 2016-17.

14. Statistics South Africa. Community Survey. 2016;

15. Agency HD. South Africa: Informal settlement status. 2012.

16. Statistics South Africa. The labour market Q1: 2018. 2018;

17. Steer RA, Kumar G, Beck AT, Beck JS. Dimensionality of the Beck youth inventories with child psychiatric outpatients. J Psychopathol Behav Assess. 2005;27(2):123–31.

18. Kovacs M. The Child Depression Inventory 2 - Professional Manual. San Antonio, TX: Psychological Corporation; 2010.

19. The Center for Epidemiological Studies Depression Scale Revised [Internet]. Available from: https://cesd-r.com

20. Kohout F, Berkman L, Evans D, Cornoni-Huntley J. Two shorter forms of the CES-D depression symptoms index. J Aging Health. 1993;5(2):179–93.

21. Frank D, DeBenedetti A, Volk R, Williams E, Kivlahan D, Bradley K. Effectiveness of the AUDIT-C as a Screening Test for Alcohol Misuse in Three Race/Ethnic Groups. J Gen Intern Med. 2008;23(6):781–7.

22. Campbell JC. Health consequences of intimate partner violence. Lancet. 2002 Apr 13;359(9314):1331–6.

23. Global Burden of Disease Collaborative Network. Global Burden of Disease Study 2017 (GBD 2017) Results [Internet]. Seattle, United States; 2018. Available from: http://ghdx.healthdata.org/gbd-results-tool

24. Norman R, Bradshaw D, Schneider M, Jewkes R, Mathews S, Abrahams N, et al. Estimating the burden of disease attributable to interpersonal violence in South Africa in 2000. South African Med J. 2007;97(8):653–6.

25. Jan S, Ferrari G, Watts CH, Hargreaves JR, Kim JC, Phetla G, et al. Economic evaluation of a combined microfinance and gender training intervention for the prevention of intimate partner violence in rural South Africa. Health Policy Plan [Internet]. 2011 Sep [cited 2013 Feb 25];26(5):366–72. Available from: http://www.ncbi.nlm.nih.gov/pubmed/20974751

26. Ochalek J, Lomas J, Claxton K. Estimating health opportunity costs in low-income and middle-income countries: A novel approach and evidence from cross-country data. BMJ Glob Heal. 2018 Jan 1;3(6).

27. Chatterji S, Stern E, Dunkle K, Heise L. Community activism as a strategy to reduce intimate partner violence (IPV) in rural Rwanda: Results of a community randomised trial. J Glob Health [Internet]. 2020 Jun 1 [cited 2021 May 24];10(1). Available from: https://pubmed.ncbi.nlm.nih.gov/32257154/

28. Michaels-Igbokwe C, Abramsky T, Devries K, Michau L, Musuya T, Watts C. Cost and cost-effectiveness analysis of a community mobilisation intervention to reduce intimate partner violence in Kampala, Uganda. 2016;

29. Leight J, Deyessa N, Sharma V. Cost-effectiveness analysis of an intimate partner violence prevention intervention targeting men, women and couples in rural Ethiopia: Evidence from the Unite for a Better Life randomised controlled trial. BMJ Open [Internet]. 2021 Mar 29 [cited 2021 Apr 19];11(3):42365. Available from: http://bmjopen.bmj.com/

30. Greco G, Knight L, Ssekadde W, Namy S, Naker D, Devries K. Economic evaluation of the Good School Toolkit: an intervention for reducing violence in primary schools in Uganda. BMJ Glob Heal [Internet]. 2018 Apr 1 [cited 2021 Oct 28];3(2):e000526. Available from: https://gh.bmj.com/content/3/2/e000526
