# Supplementary material for: Prevention of violence against women and girls: A cost-effectiveness study across 6 low- and middle-income countries
Source: PLoS Med. 2022 Mar 24;19(3):e1003827. doi: 10.1371/journal.pmed.1003827 (PMC8946747; doi:10.1371/journal.pmed.1003827)
Supplement: S1 Checklist — (DOCX) [file pmed.1003827.s002.docx]

## Reporting Checklist for Cost-effectiveness Analyses (Sanders et al., 2016)

| Element | Journal Article | Technical Appendix |
| --- | --- | --- |
| Introduction |  |  |
| Background of the problem | “Thirty percent (Uncertainty Interval 26-34%) of women” (Introduction, paragraphs 1-4) |  |
| Study Design and Scope |  |  |
| Objectives | “This paper presents the first standardised multi-country cost-effectiveness analysis, to our knowledge, of interventions for the prevention of violence against women and girls” (Introduction, par. 5) |  |
| Audience | “These findings will be of interest to” (Introduction, par. 6) |  |
| Type of analysis | “using a trial-based analysis.” (Methods, par. 1) |  |
| Target populations | “Target population” (Table 1); and “The interventions address the needs of different population groups experiencing violence (Table 1).” (Methods, par. 6) |  |
| Description of interventions and comparators (including no intervention, if applicable) | “Control Group”, “Approach” and “Platform of delivery” (Table 1); “The interventions targeted different impact mechanisms” (Methods par. 5) |  |
| Other intervention descriptors (eg, care setting, model of delivery, intensity and timing of intervention) | “Platform of delivery”, “Number of implementation sessions or duration “, “Implementation Phase” in Table 1; “Selected interventions employed three types of delivery platforms” (methods par. 5) |  |
| Boundaries of the analysis; defining the scope or comprehensiveness of the study (eg, for a screening program, whether only a subset of many possible strategies are Included; for a transmissible condition, the extent to which disease transmission is captured; for interventions with many possible delivery settings, whether only one or more settings are modeled) | “Settings” “Location” and “Intervention Sites” in Table 1; “[…] prevention interventions in Ghana, Kenya, Pakistan, Rwanda, South Africa, and Zambia” and “We also selected them to be representative of […] geographies (Southern Africa, South Asia)” (Methods, par.1); “were delivered in urban (Kenya, Pakistan, South Africa, and Zambia), and rural settings (Ghana and Rwanda).” (Methods par. 5) |  |
| Time horizon | “Time horizon” Table 1 |  |
| Analytic perspectives (eg, reference case perspectives [health care sector, societal]; other perspectives such as employer or payer) | “We report cost per DALY averted from both a provider and societal perspective,” (Methods par. 3) |  |
| Whether this analysis meets the requirements of the reference case | “this analysis meets the requirement of the reference case set out by the Second Panel on Cost-Effectiveness in Health and Medicine” (Methods par. 2) |  |
| Analysis plan | “We assessed cost-effectiveness using a standardised methodology we developed for VAWG prevention and women’s empowerment interventions” (Methods par. 2). See also Ferrari et al. (2019), cited therein. |  |
| Methods and Data |  |  |
| Trial-based analysis or model-based analysis. If model-based: | “using a trial-based analysis.” (Methods, par. 1) |  |
| Description of event pathway or model (describe condition or disease and the health states included) | N/A |  |
| Diagram of event pathway or model (depicting the sequencing and possible transitions among the health states included) | N/A |  |
| Description of model used (eg, decision tree, state transition, microsimulation) | N/A |  |
| Modeling assumptions | N/A |  |
| Software used | “We conducted the analysis in Stata 15.1” (Methods, Analysis section par 8) |  |
| Identification of key outcomes | “To estimate incremental DALYS averted by each intervention compared to the status quo, we estimated the DALYs attributable to the health sequelae” (Methods, Outcomes section par. 1) |  |
| Complete information on sources of effectiveness data, cost data, and preference weights | “Outcomes” in Table 1; “To estimate incremental DALYS averted by each intervention compared to the status quo, we estimated the DALYs attributable to the health sequelae, i.e. health consequences, found for each intervention using trial data” (Methods, Outcomes section par 1); “We measured providers’ resource use and economic costs with a bottom-up micro-costing approach” (Methods, Cost estimation section par. 1, and see Torres-Rueda et al., 2020 therein for more detail on cost data sources) |  |
| Methods for obtaining estimates of effectiveness (including approaches used for evidence synthesis) | “Study-level statistical analysis for intervention effect estimates” in Table 1; “We estimate impact using the statistical model used to generate the RCT primary and secondary results,” (Methods, Analysis section par 1) | “Our main analysis reports interventions' effect on all-cause morbidity DALYs, using all measured health sequelae in the trial datasets. To arrive at these estimates, ..” (Appendix, Disability Adjusted Life Years (DALYs) section, par 1) |
| Methods for obtaining estimates of costs and preference weights | “We measured providers’ resource use and economic costs with a bottom-up micro-costing approach” (Methods, Cost estimation section, par 1) | “The paragraphs below under the heading 'Costing' are an excerpt from our cost-synthesis paper [1]. They illustrate the methods we followed to determine the incremental costs of delivery for the main analysis” (Methods par 1) |
| Critique of data quality | “economic impact data from Zambia were unavailable and no economic impact data …” (Discussion par. 8); “our effectiveness data was obtained from RCTs and may be an upper bound estimate of intervention efficacy” (Discussion par 9) | “There were discrepancies in perceptions of time use” ” (Methods, Uncertainty Analysis section par 2) |
| Statement of costing year (ie, the year to which all costs have been adjusted for the analysis; eg, 2016) | “All analyses were conducted in USD 2018 prices” (Methods, Cost-effectiveness estimates section par 1) |  |
| Statement of method used to adjust costs for inflation |  | “using the World Bank GDP deflator” (Methods, Costing section par 7) |
| Statement of type of currency | “All analyses were conducted in USD 2018 prices” (Methods, Cost-effectiveness estimates section par 1) | “Converted …to US Dollars using average annual exchange rates” (Methods, Costing section par 7) |
| Source and methods for obtaining expert judgment if applicable |  | “Moreover, intervention modifications may be necessary when interventions are scaled up from pilot to national levels. Consequently, we consulted with senior members of each implementation team” (Methods, Scale Up analysis section par3) |
| Statement of discount rates | “costs were discounted using a rate of 3%,” (Methods, Cost-effectiveness estimates section par 1) |  |
| Impact Inventory |  |  |
| Full accounting of consequences within and outside the health care sector | “The impact inventories report on outcomes relevant to” (Results par 8) | “Impact inventories” section |
| Results |  |  |
| Results of model validation |  | “We first generate 10,000 independent random draws from each distribution and test that the resulting realisations are distributed as expected” (Probabilistic sensitivity analysis - distributional assumptions and parameters par 2) |
| Reference case results (discounted and undiscounted): total costs and effectiveness, incremental costs and effectiveness, incremental cost-effectiveness ratios, measures of uncertainty | “From a societal perspective, per participant results” (Results par 1-4); Tables 3 and 4 |  |
| Disaggregated results for important categories of costs, outcomes, or both | “Table 3 presents the summary costs for each intervention. Provider costs per participant range from” (Results par 1) Tables 2, 3 | Cost per year free from violence section, par 1 and Table 7 |
| Results of sensitivity analysis | line 520-524 | Additional analyses section |
| Other estimates of uncertainty | N/A | N/A |
| Graphical representation of cost-effectiveness results | N/A |  |
| Graphical representation of uncertainty analyses | Figures 1-6 | Figures A2-A4 and Figures A7-A8 |
| Aggregate cost and effectiveness information | Table 4 |  |
| Secondary analyses | “The sub-population analysis for the Rwanda Couples intervention finds” (Results par 8) | Rwanda – couples’ cost-effectiveness section |
| Disclosures |  |  |
| Statement of any potential conflicts of interest due to funding source, collaborations, or outside interests | “Charlotte Watts is the Chief Scientific Adviser at the Foreign and Commonwealth Development Office (FCDO).” Declaration of interests section. |  |
| Discussion |  |  |
| Summary of reference case results | “We find that nearly all the interventions evaluated demonstrate a positive impact on health and economic well-being (and other outcomes).” (Discussion par 1-2) |  |
| Summary of sensitivity of results to assumptions and uncertainties in the analysis | “Results from our sensitivity analyses do not alter our general conclusions” (Discussion par 8) | “These results present similar patterns to the cost-effectiveness analysis based on all-cause morbidity for South Africa and Ghana … ” (IHME DALY from IPV section par 1) |
| Discussion of the study results in the context of results of related cost-effective analyses | “Comparisons with the cost-effectiveness of similar interventions are limited” (Discussion par 5) | “Ghana compares favourably to SASA!'s US$ 460 (2011 prices) reported by Michales-Igbokwe et al [12] and to UBL’s US$ 194 (2015 prices” (Cost per year free from violence section) |
| Discussion of ethical implications (eg, distributive implications relating to age, disability, or other characteristics of the population) | “We did not explore further sub-group analysis by age group, because it is unlikely that implementation would target only specific age groups” (Cost effectiveness estimates section, par 6) |  |
| Limitations of the study | “However, our study has several limitations. …” (Discussion par 8) |  |
| Relevance of study results to specific policy questions or decisions | “Findings suggest that investment in VAWG can improve population health even in low resource settings … ” (Conclusion par 1) |  |

## Structured abstract

| **Element** | **Suggested Content** | **Excerpts** |
| --- | --- | --- |
| Objective | Succinctly state the research question specific to the analysis. | We report on the costs and health impact of VAWG prevention in six countries |
| Interventions | List all interventions included in the analysis, including the comparators. Identify the time frame of the interventions. | … VAWG prevention interventions using primary data from five randomised controlled trials in sub-Saharan Africa and one in South Asia.  All interventions were delivered between 2015 and 2018 and were compared to a do-nothing scenario, except for one of the school-based interventions (Government-mandated programme) and for the combined intervention (access to financial services in small groups). |
| Target population | Identify the age ranges, clinical characteristics, and other characteristics for all subgroups evaluated in the analysis. | Target populations: adolescents (11-14), and men and women (18+ years old) |
| Perspectives | Identify whether the analysis uses the reference case perspective and any alternative perspective presented. | “From a health sector perspective” and “Taking a societal perspective” |
| Time horizon | Specify the time horizon for the analysis. This may differ from the time frames of the interventions and the comparators. | “one-year time horizon” |
| Discount rate | Specify the discount rate used in the analysis. | “We use a 3% discount rate” |
| Costing year | Specify the costing year used in the analysis. | US$ […] (2018) |
| Study design | Describe whether this is a trial-based or model-based analysis. If it is a model-based analysis, briefly describe the model type (eg, decision tree, state transition, microsimulation, discrete event) and the size and characteristics of the simulated population. Indicate whether the analysis meets the reference case requirements. | “We conducted a trial-based cost-effectiveness analysis” |
| Data sources | Describe the types of data used to derive inputs for the analysis (eg, primary data, secondary data from the published literature, administrative data, unpublished trial data). | “… using primary data from five randomised controlled trials” |
| Outcome measures | List primary and secondary outcome measures (eg, incremental cost-effectiveness ratio in dollars per quality-adjusted life-year, dollars per life-year, or dollars per clinical end point; total costs; total quality-adjusted life-years for a specified cohort; or population-level outcomes). | “We computed the health burden from VAWG with disability-adjusted life-year (DALY).” “We report cost-effectiveness as cost per DALY averted,” |
| Results of analysis |  |  |
| Base case | Briefly describe the results for the primary outcome measures, as well as the notable results for intermediate outcomes and disaggregated results (eg, deaths averted, hospitalizations averted, specific subcategories of costs). Identify any substantial changes in non–health-care–sector consequences. | “From a health sector perspective, the cost per DALY averted varies between US$ 222 (2018),…” |
| Uncertainty | Briefly describe whether the results are robust to changes explored in the uncertainty analyses. | “Results are robust to sensitivity analyses.” |
| Limitations | Describe important limitations of the analysis such as controversial assumptions. | “Main limitations:” |
| Conclusions | Summarize the key clinical or policy conclusions | “We demonstrate that investment in established community based VAWG prevention interventions can improve population health in LMICs, even within highly constrained health budgets” |
