# Supplementary material for: Prevention of violence against women and girls: A cost-effectiveness study across 6 low- and middle-income countries
Source: PLoS Med. 2022 Mar 24;19(3):e1003827. doi: 10.1371/journal.pmed.1003827 (PMC8946747; doi:10.1371/journal.pmed.1003827)
Supplement: S1 Translations — (PDF) [file pmed.1003827.s003.pdf]

Translations

We aim to make our findings accessible to a wide audience, so some of us volunteered to translate abstract and lay summary into some of the languages of the communities we worked with. We also provide a Spanish translation, because Spanish is one of the most widely spoken languages globally. We had no access to translators into Chinese or Hindi. We made every effort to eliminate errors, including back-translating into English to check the soundness of the initial translation, where possible. We acknowledge these translations may contain inaccuracies. We recommend readers refer to the original English abstract and author summary as the official version of these documents in case of conflicts with, or lack of clarity in, any of the translations.

Akan Twi

Translators: Theresa Tawiah and Delia Bandoh. We thank Deda Ogum Alangea for her review of the translation.

Ɛkwan a yɛbɛ fa so asi ayayadeɛ a yɛyɛ ɛtia mmaa ho nkontabuo dwumadie nsem a efiri aman nsia wɔnsikasem wɔ fɔm

Ɛdin tiawa: Nkontabuo a ɛfa ɛkwan a yɛbɛ fa so asi ayayadieɛ a yɛyɛ de tia mmaa

Dwumadie no tɔfa

NNYINASOƆ (BACKGROUND): Ayayadeɛ a yɛ ye de tia mmaa (VAWG) yɛ adeɛ a ɛtia nnipa fahodie so na ɛsan nya asetena mu ne mpɔtam, sikasɛm, ne apomuden nsunsuansoɔ wɔ wɔna ɔfa saa ɔhaw yi mu ne wɔn a ɔdi saa abenefo sem yin so. Adanseɛ a ɛmu yɛ duru a ɛkyerɛ ɔhaw a enya wɔ sikasɛm, asetena ne apomuden ɛna eka wɔ bɛbɔ di asi VAWG ano no yɛ adeɛ a ɛho hia paa wɔ anammontu nhyehyɛeɛ a ɛfa sɛ yɛ de sika bɛhyɛ mu, titire wɔ aman wɔn sikasem wɔ fem a wɔ nni apomuden ho akadeɛ kɛse. Yɛde ɛka a yɛbɛbɔ ne apomuden nsunsuasoɔ a sɛ yɛdi si VAWG hu kwan hu nsem a ɛfiri aman nsia so na yɛde retodwa.

ƆKWAN YƐ FAASO NE DEƐ EFIRI MU BAEƐ (METHODS AND FINDINGS): Yɛ yɛɛ nhwɛhwɛmu dwumadie mpensempensemu a ɛgyina ankontabuo fa dwumadie yɛde si VAWG hu kwan, yɛde nsemmisa a edika koraa efiri nhwɛhwɛmu dumadie efa kwan a y'ɛde si VAWG hu kwan a efiri aman nsia wɔ yii wɔn wɔ "Sub-Saharan Africa" a ɛmu baako nso wɔ "South Asia". Yɛbu nkonta faa nhwehwɛmu dwumadie a mmienu ekɔɔ so wɔ sukuu mu a yɛ de sukuu nkwadaa a w'asi wɔn panin fie kwan (11-14 years) so na ɛdi dwuma no, ene adwumayɛfoɔ kuo mmienu wɔ anamɔ ntuo dwumadie no mu, a ena nsrano amamɔntuo kuo ketoa baako, ena yɛ kaa kuo ketoa baako, ne mpɔtam kuo a ɛmu nnipa "mmarima ne mmaa" a wɔn ani afi (18+ years old) bɔɔ nnu yɛ nsiano dwumadie.

Saa nsiano (anamɔntuo) dwumadie yi nyinaa si wɔ afe 2015 ne 2018 ntam. Na yɛ de totoo deɛ na ɛwɔ hɔ dada no ho gye sukuu kuo bako a ɛka nsia no dwumadie na nsia no anamɔtuo kuo a yɛka bom no nyaa sikafam mmoa. Yɛde disability adjusted life year (DALY) ɛseseɛ ɔhaw a VAWG wɔ apomuden so. Yɛde nkontabuo nhwesoɔ a ɛkyerɛɛ nhwehwemu dwumadie no mu biara nhehyeeɛ ana ensisi so ahyɛaseɛ no na ɛsusuu DALY maa ankorɛkorɛ biara. Yɛbuu nkonta seseɛ ɛkuo a na ɛka akuoo nketoa no ho wɔ ansiano anamɔtuo dwumadie no mu a na ɛhyɛ akuo na y'aka abom no hu, ena yɛ seseɛ akuo foforɔ binom a na enka ho. Yɛ san buu nkonta de kyerekyerɛɛ anamɔntuo no nsunsuasoɔ a enyaa wɔ asetena mu sika nsem so a na ɛboro apomuden so. Yɛde tee ɔha mu nkyekyemu miensa (3%) firi sika a yɛde hyɛɛ mu wɔ afe ntam susu sɛ ɔhaw biara mma wɔ anamɔntuo no akyi wɔ apomuden fa mu no, eka wɔ DALY biara ho sesa firi US$222 wɔ afe 2018 na ɛboa de nsesaeɛ baa mmaa ne mmerima su ɛne aserena mu nnerɛ bone wɔ apɔtam anamɔntuo dwumadie a ɛkɔ so wɔ Ghana kɔ duruu US$17,548 wɔ 2018 wɔ asetena mu dwumadie a ɛkɔ so wɔ South Africa. Sɛ yɛfa nno asafo fa mu na yɛ de sika fa mu nsunsuansoɔ nso ka ho a, sika yɛ de hyɛɛ mu no so baa mfasoɔ wɔ anamɔntuo dwumadie no bi mu na mom etee ɛbi nso deɛ so. Ɛbi tesɛ annamɔntuo dwumadie ene sikasɛm ho ntotoeɛ pa a menpii no wɔn a wɔ wɔ sika sem mu botaeɛ a ɛmu dahɔ, yɛtee dwumadie no ka so na ema yɛ nyaa nsunsuanso pa firi sika yɛ de hyɛɛ anamɔntuo dwumadie no mu. Deɛ efiri mu baeɛ no bɛ hia nkyerɛkyerɛ a ɛmu dɔ.

NEƐ A ƐSI KWAN AKASA (MAIN LIMITATIONS)

yɛn DALYs nsusuyɛ no mu no, yɛde akuo a ɛfiri apomuden ho nsusuanso a na ɛyɛ wɔn a wɔn wɔbɛtumi akɔ VAWG haw mu eka ho; yɛ susu sɛ ɔhaw a ɛbɛba no mu biara nni hɔ a ɛbɛ wie owuo wɔ akontabuo no mu y'anfa ɔwuo anka DALYs. Na yɛ bɛtumi ate apomuden mu haw biara wɔ saa nsem mmienu no mu. Afei nso y'ammu akonta anfa nipa biara na ɔka dwumadie no ho aponuden ho ka.

AWIEYƐ (CONCLUSION)

Yɛre kyereɛ sɛ, sika a ye de hyɛ nsiano anamɔtuo wɔ VAWG ho wɔ mpɔtam bɛ tumi aboa ama amansan apomuden atumpon wɔn aman a wɔn sikasem wo fem mu. Mpo sɛ ɔman no apomuden akohoma sika sua a. Ne ɛmom, VAWG nsiano anamɔntuo bebree bɛhia nsakraeɛ bi a ɛbɛ boa ate ka so na ama sika ɛkɔ mu no aso aba pa. Wɔ ne fa kɛseɛm mu no wɔ asetena mu, apomuden mu ɛne sikasɛm nsusuansoɔ mu sɛ yɛde sika bɛhyɛ nhwehwɛmu mu na aso

aba pa no yɛ ade hia ɛsɛ sɛ wɔyɛ de kyerɛ sɛ VAWG nsiano ho hia animanimu yi ara wɔ amansan nyinaa mu.

Tɔfa a nwoma twerefoɔ no de to dwa

Deɛn nti na yɛ dii dwuma yie (Why was this study done)

1. Aban ahodoɔ rekɔ so de sika hyɛ mu sɛ ɔbɛ yi ayayadeɛ a yɛ de tia mmaa paninfoɔ ne mmasiafoɔ afiri wɔ afe 2030 mu sɛ deɛ sustainable development goal 5 nhyehyerɛ teɛ no.
2. Adanseɛ a ɛbɛboa ama yɛ de sika ahyɛ mu no sua koraa, mpo wɔ aman a wɔn sikasem wɔ fem mu. saa akwanside3 yi aasi nhyehyɛɛɛ a wɔretrɛ mu de ako atia ayayadeɛ a yɛ yɛ de tia mmaa panifoɔ dwumadie ano kwan
3. Mpensenpensemu a yɛde rehwehwɛ nhyehyɛyɛ a ɛbɛ ma y'anya sika a wode kyɛ mu ho mfaso pa wɔ apomuden fa mu ne deɛ enyɛ apomuden fa mu, saa dwumadie yi bɛ boa ama wɔn a wɔbɔ wɔ ho mmɔden sɛ wɔ bsi ayayadeɛ a yɛ de tia mmaa ano no bɛ nya nyinasoɔ a ɔde bɛnya sika afiri apomuden asoeɛ ne asoeɛ foforɔ a wɔdwen mmaa apomuden nkankɔɔ ne mmaa yiedie ho.

Deɛn na nhwehwɛmufoɔ no yɔeɛ ene deɛ ɔmo hunueɛ (What did the researchers do and find?)

1. Yɛdi nsɔhwɛ dwumadie rekyerɛ sika a yɛbɛ tumi de ayɛ nkyekyɛyɛ pa de asi ayayadeɛ a yɛ de tia mmaa panifoɔ ne mmasiafoɔ wɔ aman nsia mu: Ghana, Kenya, Pakistan, Rwanda, South Africa ne Zambia.
2. Yɛ huu sɛ anamɔntuo no mu bi wɔ hɔ a ɛbɛtumi aboa ama amansan apomuden nsɛm atu mpon, mpo ɛwɔ mprenpren yi a aman no mu biara mfaa ɛhu sika nkaa wɔn apomoden sikasem ho. Anamɔntuo no bɛtumi asi mmaa a ɔmo kɔ saa ɔhaw no mu no ano asene mmarima a wɔyɛ atitrasɛm. Ankorɛ korɛ afutuo ɛboa ma nsiano anamontuo a ɛtɔ so mmienu ayɛ mfasodeɛ na asane ayɛ fo sen sɛ ye de y'ani bɛsi ɔhaw no nkoa so apɛ nsusuansoɔ pa wɔ mmiamia a sikasem wɔ fem. Wɔ apomuden fam no, mpɔtam ne sukuu nsiano anamɔntuo no nyinaa bɛtumi aboa bebree ama yɛnya sika ɔde behyɛ mu no so aba pa.
3. Sɛ yɛ hwɛ nsusuansoɔ no nyinaa, anamɔntuo a ɛboa wɔn a wɔde wɔ ho hyɛɛ dwumadie no mu wɔ asetena mu nimdeɛ te sɛ sikasɛɛm ntotoeɛ, ɛtumi tee ka so, na ɛsan te

mmarima a wɔdi saa benefosɛm no so, mpo sɛɛ antumi ante nyansasuaa mmaa paninfoɔ ne mmasiafoɔ wɔɔ wɔ ayayadeɔ ho wɔ mmere tia mu.

Nea wohunu yɛ no asekyereɛ sen? (WHAT DO THESE FINDINGS MEAN)

1. Adanseɛ no susu sɛ mpatamumpatumu nhyehyɛyɛ a yɛ de si ayayadeɛ yɛyɛ tia mmaa no hu hia sɛ yedwneneho sɛ y'ɛbetremu
2. Na ɛmom, sɛ yɛpɛ sɛ amansan nyinaa nya nhyehyɛyɛ pa no a, ahia s3 akadeɛ aa ɛbɛ boa ama w'akɔ so ayɛ nsiano nhyehyɛɛɛ ne ntotoeɛ ahodoɔ a ɛbɛ nya nsusuanso pa, na ɛnfa ka mma abrɛ wɔ gu so retete adwumayɛfoɔ a ɔnimde wɔ aman wɔn sikasem wɔ fem.
3. Nhwehwɛmu dwumadie sika bɛ hia ama w'atumi a nya nsunsuanso pa, ɔkwan ne sikasɛm ho akonta a yayadeɛ a yɛyɔ de tia mma paninfoɔ ne nkoraa nsiaano na enyɛ maa a ɔkɔ saa ɔhaw yi mu ne wɔn a wɔ yɛ mmaa ayayadeɛ no nkoaa, na ne fa kaseɛ mu wɔ apomuden mu nsunsuansoɔ ne asunsuansoɔ afoforɔ ɛbɛ boa ama nyinasoɔ kɛseɛ a ɛbɛ boa ama yɛ de sika ahyɛ ayayadeɛ a yɛ de tia maa nsiano mu wɔ amansan nyinaa mu

Nyanja (Chichewa)

Translator: Elenah Kapapa

Kupewa nkhanza kwa amayi ndi atsikana: Kafukufuku wothandiza kwambiri m'maiko asanu ndi limodzi otsika ndi apakati

Mutu wachidule: Kutsika mtengo popewa nkhanza kwa amayi ndi atsikana

Ndemanga

Kubwerera nthaka: Nkhanza kwa amayi ndi atsikana (VAWG) ndi kuphwanya ufulu wa anthu ndi chikhalidwe, zachuma, chikhalidwe ndi thanzi Zotsatira za opulumuka, ochita zoipa ndi anthu. Umboni wamphamvu pa zachuma, chikhalidwe ndi thanzi zimakhudza kuphatikiza mtengo woperekera Kupewa kwa VAWG ndikofunikira kwambiri kuti tipeze ndalama, makamaka m'maiko omwe ali ndi ndalama zochepa komanso zapakati (LMICs) ku gawo la zaumoyo kwamene chuma nicho kakamizidwa kwambiri. Timapereka lipoti za mtengo ndi zotsatira zaumoyo za kupewa kwa VAWG m'maiko asanu ndi limodzi.

Njira ndi Zomwe Zapeza: Tinachita kafukufuku wokhudzana ndi mtengo wa VAWG wogwiritsa ntchito deta yoyambirira kuchokera ku mayesero asanu olamulidwa mwachisawawa ku sub-Saharan Africa ndi ku South Asia. Tinayesa sikulu awiri zochokera kulowererapo cholinga pa achinyamata (zaka 11-14); ndi ziwiri zokambirana zochokera (kagulu kakang'ono kapena m'modzi-kwa-m'modzi) kulowererapo, kulowererapo kumodzi kwa anthu ammudzi ndi chimodzi kuphatikiza gulu laling'ono ndi dera zochokera kulowererapo (wazaka 18+). Zonse njira kulowererapo anaplierekedwapakati pa 2015 ndi 2018 ndi anali poyerekeza ku a osachita chilichonse, kupatula mmodzi wa sukulu zochokera kulowererapo (Pulogalamu yolamulidwa ndi boma) ndi kulowererapo kophatikizana (kupeza ntchito zachuma m'magulu ang'onoang'ono). Tinawerengera kulemedwa kwaumoyo kuchokera ku VAWG ndi kulumala kusinthidwa chaka cha moyo (DALY). Ife tinayerekeza pa munthu DALYs kupewedwa pogwiritsa ntchito ziwerengero zomwe zikuwonetsa chilichonse kapangidwe ka mayeso ndi chiyambi chilichonse kusalinganika. Timapereka lipoti mtengo wogwira mphamvu monga mtengo pa DALY apewedwa, ndi khalidwe kusatsimikizika mu kuyerekezera ndi chotheka kumva kusanthula ndi mtengo wogwira kuvomerezeka zokhota (CEACs), zomwe zikuwonetsa kuthekera kwa mtengo mphamvu pazigawo zosiyanasiyana. Timapereka lipoti kusanthula kwamagulu wa gulu laling'ono gawo cha kuphatikiza kulowererapo, ndipo palibe kusanthula kwina kwamagulu. Timaperekanso lipoti kukhudza kufufuza kufotokoza kuthandizira' chikhalidwe-chuma zotsatira kupitirira thanzi. Timagwiritsa ntchito kuchotsera kwa 3% mtengo wa ndalama mtengo ndi nthawi ya chaka chimodzi, poganiza ayi zotsatira positi nthawi yolowererapo.

Malinga ndi gawo la zaumoyo, mtengo pa DALY kupewedwa zimasiyanasiyana pakati pa US $ 222 (2018), kwa chikhalidwe chokhazikika cha jenda ndi zovulaza chikhalidwe machitidwe kusintha zokhala mdera kulowererapo Ghana, ku US $ 17,548 (2018) za a zopezera ndalama kulowererapo ku South Africa. Kutenga chikhalidwe cha anthu ndi kuphatikiza zambiri zotsatira zachuma bwino ndi mtengo wogwira mwa ena kulowererapo koma amachepetsa ena. Mwachitsanzo, kulowererapo ndi zabwino zotsatira zachuma, nthawi zambiri omwe ali nawo poyera zolinga zachuma, kuchepetsa kukhazikitsa ndalama ndi kukwaniritsa zabwino kwambiri mtengo wogwira chiwerengero. Zotsatira ndi wamphamvu ku kumva kusanthula.

Zoletsa zazikulu: ma DALY athu akuphatikizapo kagawo kakang'ono ka zotsatira za thanzi la VAWG; timaganiza kuti palibe imfa kukhudza chilichonse cha zotsatira za thanzi kuphatikizidwa mu zowerengera za DALY. Muzochitika zonsezi, titha kukhala ochepera kukhudza thanzi lonse. Komanso sitipereka lipoti otenga nawo mbali' ndalama zaumoyo.

Mapeto: Tikuwonetsa kuti ndalama mu kukhazikitsidwa kupewa kwa VAWG m'madera kulowererapo zitha kupititsa patsogolo thanzi la anthu mu LMICs, ngakhale m'munsi kwambiri ndalama zochepetsera zaumoyo. Komabe, kupewa VAWG zingapo amafuna zina kusinthidwa kukwaniritsa kukwanitsa ndi zotsika mtengo pa sikelo. Kukulitsa osiyanasiyana chikhalidwe, zotsatira zaumoyo ndi zachuma adzalandidwa mtsogolo mtengo wogwira kuwunika imakhalabe yofunika ku kulungamitsa ndalamazo zofunika kuchita mwachangu kuletsa VAWG padziko lonse lapansi.

Chiwerengero cha mawu: 6,360

Chidule cha Wolemba

N'chifukwa Chiyani Phunziroli Linachitika?

- Maboma ali kuwonjezera mtengo kwa kuchotsedwa kwa nkhanza kwa amayi ndi atsikana pofika 2030 ngati gawo la cholinga cha chitukuko chokhazikika chachisanu

- Umboni kuti dziwitsani ndalama m'dera lino ndi ochepa kwambiri, kuphatikiza ochokera kumayiko omwe ali ndi ndalama zochepa komanso zapakati, kusonyeza chachikulu chopinga pakukulitsa nkhanza kwa amayi kupewa kupanga mapulogalamu.

- Kufufuza za kuthekera zotsika mtengo, thanzi ndi osakhala thanzi zotsatira za kupewa amathandiza omwe amagwira ntchito nkhanza kwa amayi kupewa fotokozani ndalama kuchokera ku nthambi yazaumoyo kapena zigawo zina chidwi ndi thanzi ubwino ndi ubwino wa mkazi.

Kodi Ofufuza Anachita Chiyani Ndipo Anapeza Chiyani?

- Timapereka malipoti mayesero potengerakusungitsa ndalama kuyerekezera njira zisanu ndi imodzi opangidwa kuti apewe nkhanza kwa amayi ndi atsikana m'mayiko asanu ndi limodzi: Ghana, Kenya, Pakistan, Rwanda, South Africa ndi Zambia.

- Ife tikupeza kuti ena kulowererapo zikutheka kupititsa patsogolo thanzi la anthu, ngakhale pakali pano bajeti zaumoyo m'dziko lililonse. Kulowererapo zotheka kukhala zotsika mtengo pa kupewa kuwonekera kwa amayi ku nkhanza, m'malo moti zochita za amuna. Mmodzi-kwa-mmodzi chithandizo chamaganizo ndi chikhalidwe kulowererapo chitetezo chachiwiri, pomwe amakhudza, zitha kukhala zotsika mtengo kuposa kupewa koyamba kulowererapo m'makonzedwe otsika kwambiri. Kumudzi komanso kusukulu kulowererapo zotheka kutero kukhala otsika mtengo kuchokera ku thanzi kaonedwe.

- Poganizira zotsatira zonse, njira zomwe zimathandizira otenga nawo mbali ' luso la moyo, kuphatikizapo awo luso loyang'anira zachuma, akhoza kupulumutsa mtengo, komanso kuchepetsa kuchititsa ziwawa kuchokera kwa amuna, ngakhale osachepetsa zochitika zachiwawa pakati pa amayi ndi atsikana mu nthawi yochepa.

Kodi Zotsatirazi Zikutanthauza Chiyani?

- Umboni ukusonyeza kuti anakhazikitsa njira zoyendetsera anthu pofuna kupewa nkhanza kwa amayi kuganiziridwa kwa chilolezo kuti muwonjezere msanga.

- Komabe, kufikira anthu onse pakufunika ndi zoyenera kulowererapo, ndalama zambiri ndi zofunika kuwonjezera kulitsa ndi kuyenga osiyanasiyana kupewa zoperekera zitsanzo kuti zolimbikitsa ndi ali ndi mtengo, pamene akukula gwero la anthu ukatswiri m'maiko otsika ndi apakati.

- Ndalama zofufuzira ndizofunikira kupitiriza kuyatsa mphamvu yamphamvu, ndondomeko ndi zachuma kuwunika kwa nkhanza kwa amayi ndi atsikana kupewa zomwe zimatengera mphamvu osati pa chiwawa chokha kukhumudwa ndi kukhumudwa, , komanso thanzi labwino ndi zotsatira zosakhudzana ndi thanzi kuonetsetsa zamphamvu mlandu wa ndalama popewa nkhanza kwa amayi ndi atsikana ikupitilira kupangidwa padziko lonse lapansi.

Spanish

Translator: Sergio Torres-Rueda

Prevención de la violencia contra las mujeres y las niñas: un estudio de costo-efectividad en seis países de ingresos bajos y medios

Título corto: Costo-efectividad de la prevención de la violencia contra las mujeres y las niñas

Resumen

Antecedentes: La violencia contra las mujeres y las niñas (VCMN) es una violación de los derechos humanos con consecuencias sociales, económicas y de salud para las sobrevivientes, los perpetradores y la sociedad. La evidencia robusta sobre el impacto económico, social y sanitario, más el costo de la prevención de la VCMN, es fundamental para informar la inversión de recursos, en particular en los países de ingresos bajos y medios (PIBM) donde los recursos del sector de la salud son muy limitados. Reportamos los costos y el impacto en la salud de la prevención de la VCMN en seis países.

Métodos y hallazgos: Realizamos un análisis de costo-efectividad basado en ensayos de las intervenciones de prevención de la VCMN utilizando datos primarios de cinco ensayos controlados aleatorizados en África subsahariana y uno en el sur de Asia. Evaluamos dos intervenciones escolares dirigidas a adolescentes (11-14 años); y dos intervenciones basadas en talleres (en grupos pequeños o individuales), una intervención en la comunidad y un programa combinado en grupos pequeños y en la comunidad, todos dirigidos a hombres y mujeres adultos (mayores de 18 años). Todas las intervenciones se realizaron entre 2015 y 2018 y se compararon con un grupo de control sin tratamiento, excepto una de las intervenciones basadas en la escuela (programa ordenado por el gobierno) y la intervención combinada (acceso a servicios financieros en grupos pequeños). Calculamos la carga de salud de la VCMN usando el año de vida ajustado por discapacidad (AVAD). Estimamos los AVAD per cápita evitados utilizando modelos estadísticos que reflejan el diseño de cada ensayo y cualquier desequilibrio inicial. Reportamos la costo-efectividad, medida como costo por AVAD evitado, y caracterizamos la incertidumbre en las estimaciones con un análisis de sensibilidad probabilístico y curvas de aceptabilidad de la costo-efectividad, que muestran la probabilidad de costo-efectividad bajo diferentes umbrales. Presentamos un análisis de subgrupos del componente de grupos pequeños de la intervención combinada y ningún otro análisis de subgrupos. También reportamos un inventario de impacto para ilustrar el impacto socioeconómico de las intervenciones más allá de la salud. Usamos una tasa de descuento del 3% para los costos de inversión y un horizonte temporal de un año, asumiendo que no hay efectos posteriores al período de intervención.

Desde la perspectiva del sector de la salud, el costo por AVAD evitado varía entre US $ 222 (2018), en la intervención comunitaria en Ghana que tiene como objetivo el cambio de actitudes de género establecidas y normas sociales dañinas, a US $ 17,548 (2018) en una intervención enfocada en el sustento económico en Suráfrica. Adoptar una perspectiva social e incluir un impacto económico más amplio mejora la costo-efectividad de algunas intervenciones, pero reduce otras. Por ejemplo, las intervenciones con impactos económicos positivos, a menudo aquellas con objetivos económicos explícitos, compensan los costos de implementación y logran relaciones de costo-efectividad más favorables. Los resultados son robustos a los análisis de sensibilidad.

Limitaciones principales: nuestros AVAD incluyen un subconjunto de las consecuencias para la salud de la exposición a la VCMN; asumimos que no hay impacto en la mortalidad de ninguna de las consecuencias para la salud incluidas en los cálculos de AVAD. En ambos casos, es posible que estemos subestimando el impacto general en la salud. Tampoco reportamos los costos de salud de los participantes.

Conclusiones: Demostramos que la inversión en intervenciones establecidas de prevención de la VCMN basadas en la comunidad podrían mejorar la salud de la población en los países de ingresos bajos y medianos, incluso dentro de presupuestos de salud muy restringidos. Sin embargo, varias intervenciones de prevención de la VCMN requieren modificaciones adicionales para lograr asequibilidad y costo-efectividad a gran escala. Ampliar la gama de resultados sociales, sanitarios y económicos capturados en futuras evaluaciones de la costo-efectividad sigue siendo fundamental para justificar la inversión que se requiere urgentemente para prevenir la VCMN a nivel mundial.

Numero de palabras: 6,360

Resumen del autor

¿Por qué se realizó este estudio?

• Los gobiernos están aumentando la financiación para la eliminación de la violencia contra las mujeres y las niñas para el año 2030 como parte del quinto objetivo de desarrollo sostenible.

• La evidencia para informar la inversión en esta área es extremadamente limitada, incluso en países de ingresos bajos y medianos, lo que representa un obstáculo importante para ampliar los programas de prevención de la violencia contra las mujeres.

• La investigación sobre la posible costo-efectividad, en la salud y en otros aspectos de la prevención, ayuda a quienes trabajan en la prevención de la violencia contra la mujer a justificar la financiación del sector de la salud u otros sectores interesados en la mejora de la salud y el bienestar de la mujer.

¿Qué hicieron y encontraron los investigadores?

• Presentamos estimaciones de costo-efectividad basadas en ensayos para seis intervenciones diseñadas para prevenir la violencia contra mujeres y niñas en seis países: Ghana, Kenia, Pakistán, Ruanda, Sudáfrica y Zambia.

• Descubrimos que es probable que algunas intervenciones mejoren la salud de la población, incluso dentro de los presupuestos sanitarios actuales de cada país. Es más probable que las intervenciones sean más costo-efectivas en prevenir la exposición de las mujeres a la violencia que en prevenir la perpetración por parte de los hombres. Las intervenciones de apoyo psicosocial individual para la prevención secundaria, si bien tienen un impacto, tienen menor probabilidad de ser costo-efectivas que las intervenciones de prevención primaria en entornos de bajos recursos. Las intervenciones basadas en la comunidad y la escuela tienen más probabilidad de ser costo-efectivas desde la perspectiva del sector de la salud.

• Considerando todos los efectos, las intervenciones que mejoran las habilidades de sustento económico de los participantes, incluidas sus habilidades de gestión financiera, pueden ahorrar costos, al mismo tiempo que reducen la perpetración de violencia por parte de los hombres, incluso si no reducen la experiencia de violencia entre mujeres y niñas a corto plazo.

¿Qué significan estos hallazgos?

• La evidencia sugiere que las intervenciones basadas en la comunidad establecidas para prevenir la violencia contra la mujer merecen consideración para su ampliación inmediata.

• Sin embargo, para llegar a todas las poblaciones necesitadas con intervenciones apropiadas, se requiere más inversión para desarrollar y perfeccionar una gama de modelos de prestación de servicios de prevención que tengan impacto y contengan costos, mientras se desarrolla la experiencia en recursos humanos en países de ingresos bajos y medios.

• Se requiere financiación en la investigación para continuar permitiendo una evaluación rigurosa del impacto, el proceso y la economía de la prevención de la violencia contra las mujeres y las niñas que

capte el impacto no solo en la exposición y la perpetración de la violencia, sino también de manera más amplia en la salud, y en áreas más allá de la salud, para garantizar que el caso más fuerte para la inversión en la prevención de la violencia contra las mujeres y las niñas se siga haciendo a nivel mundial.

Urdu

Translator: Hussain Maqbool

خواتین اور لڑکیوں کے خلاف تشدد کی روک تھام: کم اور درمیانی آمدنی والے چھ ممالک میں لاگت کا مطالعہ

مختصر عنوان: خواتین اور لڑکیوں کے خلاف تشدد کی روک تھام کی لاگت کی تاثیر

پس منظر: عورتوں اور لڑکیوں کے خلاف تشدد انسانی حقوق کی خلاف ورزی ہے جس میں زندہ بچ جانے والوں ، مجرموں اور معاشرے کے لیے سماجی ، معاشی اور صحت کے نتائج ہیں۔ معاشی ، سماجی اور صحت کے اثرات پر مضبوط ثبوت ، نیز تشدد کی روک تھام کی ترسیل کی لاگت سرمایہ کاری کے لیے کیس بنانے کے لیے اہم ہے ، خاص طور پر کم اور درمیانی آمدنی والے ممالک میں جہاں صحت کے شعبے کے وسائل انتہائی محدود ہیں۔ ہم چھ ممالک میں تشدد کی روک تھام کے اخراجات اور صحت کے اثرات کے بارے میں رپورٹ کرتے ہیں۔

طریقے اور نتائج: ہم نے سب صحارا افریقہ میں پانچ بے ترتیب کنٹرول ٹرائلز اور جنوبی ایشیا میں سے ایک پرائمری ڈیٹا کا استعمال کرتے ہوئے تشدد کی روک تھام کی مداخلتوں کی آزمائش پر مبنی لاگت تاثیر کا تجزیہ کیا۔ ہم نے اسکول پر مبنی دو مداخلتوں کا جائزہ لیا جن کا مقصد نوعمروں (11-14 سال کی عمر) ہے۔ اور دو ورکشاپ پر مبنی (چھوٹے گروپ یا ایک سے ایک) مداخلتیں ، ایک کمیونٹی پر مبنی مداخلت ، اور ایک مشترکہ چھوٹے گروپ اور کمیونٹی پر مبنی پروگرام سب کا مقصد بالغ مرد اور خواتین (18+ سال کی عمر) ہیں۔ تمام مداخلتیں 2015 اور 2018 کے درمیان پیش کی گئیں اور ان کا موازنہ کچھ نہ کرنے کے منظر نامے سے کیا گیا ، سوائے اسکول پر مبنی مداخلتوں کے ایک (حکومت کی طرف سے لازمی پروگرام) اور مشترکہ مداخلت کے لیے (چھوٹے گروپوں میں مالی خدمات تک رسائی)۔ ہم نے صحت کے بوجھ کو تشدد سے معذوری ایڈجسٹ لائف ایئر (DALY) کے ساتھ شمار کیا۔ ہم نے اندازہ لگایا کہ فی کس ڈی اے ایل وائی کو اعدادوشمار کے ماڈل استعمال کرتے ہوئے روکا گیا ہے جو ہر ٹرائل کے ڈیزائن اور کسی بھی بیس لائن کے عدم توازن کو ظاہر کرتا ہے۔ ہم لاگت کی تاثیر کو فی DALY سے بچاتے ہوئے رپورٹ کرتے ہیں ، اور امکانی حساسیت کے تجزیے اور لاگت کی تاثیر کے قابل قبول وکر (CEACs) کے ساتھ تخمینوں میں غیر یقینی صورتحال کو نمایاں کرتے ہیں ، جو مختلف دہلیز پر لاگت کی تاثیر کے امکان کو ظاہر کرتے ہیں۔ ہم مشترکہ مداخلت کے چھوٹے گروپ جزو کے ذیلی گروپ تجزیہ کی اطلاع دیتے ہیں ، اور کوئی دوسرا ذیلی گروپ تجزیہ نہیں۔ ہم صحت سے باہر مداخلت کے سماجی و معاشی اثرات کو واضح کرنے کے لیے ایک اثر انوینٹری کی بھی اطلاع دیتے ہیں۔ ہم سرمایہ کاری کے اخراجات کے لیے 3 ڈسکاؤنٹ ریٹ اور ایک سال کے افق کا استعمال کرتے ہیں ، یہ سمجھتے ہوئے کہ مداخلت کے بعد کوئی اثر نہیں پڑے گا۔

اختتامی بیان: ہم یہ ظاہر کرتے ہیں کہ قائم کمیونٹی پر مبنی تشدد روک تھام کی مداخلت LMICs میں آبادی کی صحت کو بہتر بنا سکتی ہے ، یہاں تک کہ صحت کے انتہائی محدود بجٹ میں بھی۔ تاہم ، کئی تشدد کی روک تھام کے اقدامات کو پیمانے پر سستی اور لاگت کی تاثیر کے حصول کے لیے مزید ترمیم کی ضرورت ہے۔
مستقبل میں لاگت کی تاثیر کے جائزوں میں حاصل کردہ سماجی ، صحت اور معاشی نتائج کی حد کو وسیع کرنا عالمی سطح پر تشدد کو روکنے کے لیے فوری طور پر درکار سرمایہ کاری کو جواز فراہم کرنے کے لیے اہم ہے۔

مصنف کا خلاصہ

یہ مطالعہ کیوں کیا گیا؟

- حکومتیں پائیدار ترقی کے ہدف نمبر پانچ کے حصے کے طور پر 2030 تک خواتین اور لڑکیوں کے خلاف تشدد کے خاتمے کے لیے فنڈز میں اضافہ کر رہی ہیں۔
- اس علاقے میں سرمایہ کاری کو مطلع کرنے کے ثبوت انتہائی محدود ہیں ، بشمول کم اور درمیانی آمدنی والے ممالک ، خواتین کی روک تھام کے پروگرامنگ کے خلاف تشدد کو بڑھانے میں ایک بڑی رکاوٹ پیش کرتے ہیں۔
- ممکنہ لاگت کی تاثیر ، صحت اور روک تھام کے غیر صحت پر پڑنے والے اثرات کی تحقیقات خواتین کے خلاف تشدد میں کام کرنے والوں کو صحت کے شعبے یا صحت کی بہتری اور خواتین کی فلاح و بہبود میں دلچسپی رکھنے والے دیگر شعبوں سے فنڈ فراہم کرنے میں مدد دیتی ہے۔

محققین نے کیا کیا اور کیا پایا؟

- ہم چھ ممالک میں گھنا ، کینیا ، پاکستان ، روانڈا ، جنوبی افریقہ اور زیمبیا میں خواتین اور لڑکیوں کے خلاف تشدد کو روکنے کے لیے بنائے گئے چھ مداخلتوں کے لیے آزمائشی بنیاد پر لاگت کی تاثیر کے تخمینوں کی اطلاع دیتے ہیں۔
- ہمیں معلوم ہوا ہے کہ کچھ مداخلتوں سے آبادی کی صحت بہتر ہونے کا امکان ہے ، یہاں تک کہ ہر ملک کے موجودہ صحت کے بجٹ میں بھی۔ خواتین کے تشدد کی روک تھام کے لیے مردوں کے ارتکاب کی بجائے مداخلتیں زیادہ مؤثر ثابت ہوتی ہیں۔ ثانوی روک تھام کے لیے ایک سے ایک نفسیاتی سماجی معاونت کی مداخلت ، جبکہ مؤثر ، کم وسائل کی ترتیبات میں بنیادی روک تھام کی مداخلتوں کے مقابلے میں کم لاگت مؤثر ہونے کا امکان ہے۔ کمیونٹی اور اسکول پر مبنی مداخلتیں صحت کے شعبے کے نقطہ نظر سے لاگت سے زیادہ مؤثر ثابت ہوتی ہیں۔
- تمام اثرات کو مدنظر رکھتے ہوئے ، مداخلتیں جو شرکاء کی معاش کی مہارت کو بہتر بناتی ہیں ، بشمول ان کی مالی انتظامی مہارت ، لاگت کی بچت ہو سکتی ہے ، جبکہ مردوں سے تشدد کے ارتکاب کو بھی

کم کر سکتی ہے ، چاہے وہ مختصر مدت میں خواتین اور لڑکیوں کے درمیان تشدد کے تجربے کو کم نہ کریں۔

ان نتائج کا کیا مطلب ہے؟

- شواہد بتاتے ہیں کہ خواتین کے خلاف تشدد کو روکنے کے لیے کمیونٹی پر مبنی مداخلتیں فوری طور پر بڑھانے کے لیے غور کی ضرورت ہے۔
- تاہم ، مناسب مداخلت کے ساتھ ضرورت مند تمام آبادیوں تک پہنچنے کے لیے ، کم اور درمیانی آمدنی والے ممالک میں انسانی وسائل کی مہارت کو فروغ دیتے ہوئے ، روک تھام کی ترسیل کے ماڈلز کو مزید ترقی دینے اور بہتر بنانے کے لیے زیادہ سرمایہ کاری درکار ہے۔
- خواتین اور لڑکیوں کے خلاف تشدد کے سخت اثرات ، عمل اور معاشی تشخیص کو فعال بنانے کے لیے ریسرچ فنڈنگ کی ضرورت ہوتی ہے جو کہ نہ صرف تشدد کی نمائش اور جرم پر اثر ڈالتی ہے بلکہ صحت اور غیر صحت کے وسیع اثرات کو بھی یقینی بناتی ہے تاکہ سرمایہ کاری کے لیے مضبوط ترین کیس کو یقینی بنایا جا سکے۔ خواتین اور لڑکیوں پر تشدد کی روک تھام عالمی سطح پر جاری ہے۔

Zulu

Translators: Nwabisa Jama Shai, Smanga Mkhwanazi and Phumla Dineo

Ukuvikela kokuhlukunyezwa kwabantu besifazane namantombazane: Ucwaningo olonga izindleko emazweni ayisithupha asathuthuka nangakathuthuki ngokuphelele.

Isihloko esifushane: ukusebenza kahle kwezindleko ukuvikela ukuhlukunyezwa kwabantu besifazane namantombazane.

Okufinqiwe

Ingemumva: ukuhlukunyezwa kwabantu besifazane namantombazane, kuwukuhlukunyezwa kwamalungelo abantu ezenhlalo, ezomnotho kanye nezempilo kwabahlukunyeziwe, abahlukumezi kanye nomphakathi. Ubufakazi obunamandla mayelana nomthelela wezomnotho, ezenhlalo, nezempilo, kanye nezindleko zokuletha indlela yokuvikela ukuhlukunyezwa kwabantu besifazane namantombazane kwenza kubenzima ukuthola utshalomali, kakhulukazi emazweni asathuthuka nangakathuthuki ngokuphelele, lapho umnyango wezempilo uswele izinsiza kusebenza. Sibika ngomthelela wezindleko kanye nezempilo mayelana nokuvikela ukuhlukunyezwa kwabantu besifazane nama ntombazane emazweni ayisithupha.

Izindlela ezisetshenzisiwe nokutholakele: Senze ucwaningo olungabizi ukuhlaziya ungenelelo lokuvikela ukuhlukunyezwa olubhekiswe kwabantu besifazane namantombazane ngokusebenzisa ulwazi lokuqala esiluthole ezindaweni eziyisihlanu zase sub-Saharan Africa kanye nenye yase South Asia. Kuhlolwe ungenelelo olubili olwenziwe esikoleni olubhekiswe kumantombazane asakhula aneminyaka (11-14 ubudala); nolunye ungenelelo olubili olwenziwe endaweni yokusebenza (ngamaqembu amancane noma ngamunye ngamunye), olulodwa ungenelelo olwenziwe emphakathini, kanye nolulodwa oluhlanganise iqembu elincane kanye nohlelo olwenziwa emphakathini olubhekiswe kwabesilisa kanye nabezifazane abakhulile (18+ ubudala). Lonke ungenelelo lwenziwa phakathi kuka-2015 kuya ku-2018 lwabe seliqhathaniswa nesimo sokungenzi lutho, ngaphandle kolulodwa ungenelelo olwalenziwa esikoleni (uhlelo olugunyazwe uhulumeni) kanye nongenelelo oluhlanganise (ukufinyelela kwizinsiza zemali kumaqembu amancane).

Sibale umthwalo kwezempilo osuka ekuhlukunyezweni kwabantu besifazane namantombazane kanye nonyaka wokuphila olungiselelwe ukukhubazeka. Silinganise ukugwema unyaka wokuphila olunganiselwe ukukhubazeka womuntu ngamunye sisebenzisa izibalo ukubonisa konke okuzanyiwe kanye nokusekela okungalingani. Sibika ukusebenza kahle kwezindleko ukugwema unyaka wokuphila olungiselelwe ukukhubazeka, kanye nophawu lokungaqinisekisi ukulinganisa uhlaziyo olunozwelo kanye namajika okwamukeleka kokusebenza kahle kwezindleko, okuveza amathuba okusebena kahle kwezindleko kwimkhawulo eyahlukene. Sibika uhlaziyo ngengxenye yeqembu elincane elikhishwe kwiqembu elincane nengxenye yongenelelo oluhlanganisiwe, futhi alukho olunye uhlaziyo oluzokwenziwa lwengxenye yeqembu elincane. Siphinde sibike imthelela yokubonisa ukusungula ungenelelo kwezomnotho nezenhlalo okungaphezulu kwezempilo. Sisebenzisa isephulo esilinganiselwa ku-3% kwezosthalomali kanye nonyaka owodwa wesikhathi somkhathizwe, ngokucabanga ukuthi angeke kubekhona mphumela ngesikhathi sekuphele ungenelelo.

Ngokombono womnyango wezempilo, izindleko ukugwema unyaka wokuphila olungiselelwe ukukhubazeka zilinganiselwa ku US$ 222 (2018), ngenxa yokusungulwa kwezimo zobulili kanye nokushintsa kwezinkambiso zomphakathi eziyingozi. Ungenelelo lenhlangano yomphakathi e-Ghana lona lilinganiselwe ku US$ 17, 548 (2018) ngenxa yongenelelo lokuziphilisa eNingizimu Africa. Ukuthatha umbono womphakathi kanye nomthelela obanzi kwezomnotho kwenzangcono ukusebenza kahle kwezindleko zolunye ungenelelo kodwa kuphinde kwehlise kokunye. Isibonelo, ungenelelo olunomthelela omuhle kwezomnotho, kakhulukazi lolu olunemigomo ecacile yezomnotho, izindleko zokuqalisa uphinde uzuze okuningi okuhle kwezindleko zokusebenza ngokulinganayo. Imiphumela inamandla kuhlaziyo olunozwelo.

Ukulinganiselwa okusemqoka: ukugwema unyaka wokuphila olungiselelwe ukukhubazeka isethi engaphansi yemiphumela kwezempilo yokuchayeka kokuhlukunyezwa kwabantu besifazane amantombazane; sicabanga ukuthi awukho umthelela wokufa kwimiphumela yezempilo efaka phakathi izibalo zokugwema unyaka wokuphila olungiselelwe ukukhubazeka. Kuzo zombili lezizimo, kungenzeka ukuthi siwubukela phansi umthelela wezempilo jikelele. Futhi asibiki mayelana nezindleko zezempilo zalabo ababambe iqhaza.

Isiphetho: sikhombisa ukuthi utshalomali olusungulwe kwizinhlelo zomphakathi zokuvikela ukuhlukunyezwa kwabantu besifazane namantombazane lungenzangcono impilo yabantu emazweni asathuthuka nalawo angakathuthuki ngokuphelele, ngisho nalapho isabelomali sezempilo silinganiselwe phansi kakhulu. Noma kunjalo, izindlela eziningi zokungenelela ukuvikela ukuhlukunyezwa kwabantu bezifazane namantombazane zidinga ukuguqulwa okuqhubeka ukuzuza ukufinyelela kanye nezindleko ezingabizi kanye nokusebenza kahle kwezindleko ngesilinganiso. Ukwandisa uhla lwemiphumela yezenhlalakahle, ezempilo kaye nezomnotho efakwe ekuhlolweni kokusebenza kahle kwezindleko kusalokhu kubalulekile ekuqinisekiseni utshalomali oludingeka ngokushesha ukuvikela ukuhlukunyezwa kwabantu besifazane kanye namantombazane emhlabeni jikelele.

Ukubalwa kwamagama: 6,360

Isifinyezo sombhali

Kungani lwenziwe lolucwaningo?

- Uhulumeni wandise imali yokuqeda ukuhlukunyezwa kwabantu besifazane namantombazane ngonyaka ka-2030 njengengxenye yenhloso yentuthuko yomgomo wesihlanu.
- Ubufakazi bokwazisa utshalomali kulendawo obunqunyelwe ngokweqile, kufaka phakathi amazwe asathuthuka kanye nangathuthukile ngokuphelele, okwenza kubenzima kakhulu ukwandisa izinhlelo ezibhekiswe ekuvikeleni ukuhlukunyezwa kwabantu besifazane.
- Ukuphenya ukusebenza okungabizi kwezindleko, imithelela yokuvikela ezempilo kanye nengeyona ezempilo isizalabo abasebenza ngokuvikela kokuhlukuyezwa kwabantu besifazane ukuqinisekisa ukuxhaswa okuvela kumnyango wezempilo noma eminye imnyango enentshisekelo ekuthuthukiseni ezempilo nokuphila kahle kwabesifazane.

Yini eyenziwe yaphinde yatholwa abacwaningi?

- Sibika ukulinganiselwa kokubiza kwezindleko okususelwa kwizinhlelo zongenelelo eziyisithupha olwenzelwe ukuvikela kokuhlukunyezwa kwabantu besifazane kanye namantombazane emazweni ayisithupha: Ghana, Kenya, Pakistan, Rwanda, South Africa kanye nase Zambia.
- Sithole ukuthi ezinye izindlela zokungenelela kungenzeka zithuthukise impilo yabantu, noma ngabe kusabelomali samanje sezempilo ezweni ngalinye. Ukungenelela kungenzeka kube nezindleko ekuvikeleni kwabesifazane emathubeni odlame, kunokunhenhethekisa ukuhlukumeza kwabesilisa. Ukwelulekwa ngokomqondo komuntu ngamunye njengendlela yongenelelo lwesibili lokuvikela, ngenkathi kunomthelela, kungenzeka kungabizi kakhulu kunongenelelo lokuqala lokuvikela olunezinsiza zokusebenza ezincane. Ungenelelo lwasemphakathini nolwasesikoleni kungezeka ukuthi lubize izindleko ngokombono womnyango wezempilo.
- Ukucabanga ngayo yonke imiphumela, ukungelela okuthuthukisa amakhono kwababambe iqhaza, kufaka amakhono abo okuphatha ezezimali, kungonga izindleko, ngenkathi futhi kunciphisa ukuhlukumeza okwenziwa abantu besilisa, nomangabe akunciphisi ulwazi lokuhlukumzeka kubantu besifazane esikhathini esifishane.

Ichazani lemiphumela?

- Ubufakazi buveza ukuthi ukungenelela okusungulwe emphakathini ukuvikela ukuhlukunyezwa kwabezifazane kugunyaza ukucatshangelwa kokukhuphuka ngokushesha.
- Kodwa-ke, ukufinyelela kubo bonke abantu abadinga ungenelelo olufanelekile, kudingeka utshalomali oluningi ukuze kuqhutshekwe kuthuthukiswe futhi kwenziwe ngcono uhla lwamamodeli okuvikela anomthelela futhi aqukethe izindleko, ngenkathi kuthuthukiswa ulwazi lwabasebenzi emazweni asathuthuka nangakathuthuki ngokuphelele.
- Imali yocwaningo iyadingeka ukuze iqhubeke nokunika umthelela oqinile, inqubo kanye nokuhlolwa kwezomnotho zokuvikela ukuhlukunyezwa kwabantu besifazane namantombazane okuthinta umthelela hhayi kuphela ekuchayekeni kodlame nasekwenzeni ubugebengu, kepha futhi nemithelela ebanzi yezempilo kanye nokungeyona yezempilo ukuqinisekisa icala elinamandla kakhulu lokutshalwa kwezimali ukuvimbela ukuhlukunyezwa kwabantu besifazane namantombazane kuyaqhubeka ukwenziwa emhlabeni jikelele.
